# Does birthweight matter to quality of life? A comparison between Japan, the U.S., and India

**DOI:** 10.1186/s13561-022-00393-9

**Published:** 2022-09-20

**Authors:** Chisako Yamane, Yoshiro Tsutsui

**Affiliations:** 1 Faculty of Economics, Hiroshima University of Economics, 5-37-1, Gion, Asaminami, Hiroshima-shi, Hiroshima 731-0192 Japan; 2 Faculty of Social Relations, Kyoto Bunkyo University, Senzoku-80 Makishimacho, Uji, Kyoto 611-0041 Japan

**Keywords:** Low birthweight, Hight birthweight, Long-term associations of birthweight, Japan, the U.S., India

## Abstract

**Background:**

Birthweight is a widely accepted indicator of infant health and has significant and lasting associations. Several studies have found that low and high birthweight have significant negative associations with adult health. A new study in the field of social sciences has established that birthweight has significant negative associations with not only adult health but also social attributes, such as income and occupation; however, no studies have evaluated the associations between birthweight and quality-of-life (QOL) attributes such as happiness.

**Methods:**

In this study, we use data from Japan, the U.S., and India, collected in 2011, in which the respondents were asked about their own birthweights to examine the long-term associations between low and high birthweight and eight outcome variables related to the QOL: adolescent academic performance, height, education, marital status, body mass index, income, health, and happiness. We regressed each of the eight outcome variables on low and high birthweight and the interaction terms of the old age and the birthweight dummies for each country. We estimated both the reduced and the recursive-structural forms. While the former estimates the total, that is, the sum of direct and indirect associations between birthweight and each outcome, the latter reports the direct association between birthweight and each outcome.

**Results:**

In Japan, while low birthweight is negatively associated with all outcomes, the associations of high birthweight were limited. In the U.S., low birthweight was not associated with any outcomes, but high birthweight had significantly negative associations with health and happiness. In contrast, in India, high birthweight was significantly and positively associated with income, health, and happiness, while low birthweight was associated with several outcomes negatively, similar to Japan. These associations were stronger in youth than in old age.

**Conclusion:**

Our study demonstrated that the associations of birthweight with QOL are widely diversified across countries: low birthweight, rather than high birthweight, is a problem in Japan and India. However, the opposite is true for the U.S., indicating that policymakers in developed countries must pay closer attention to the problems caused by high birthweight, whereas those in developing countries are better to focus on low birthweight.

**Supplementary Information:**

The online version contains supplementary material available at 10.1186/s13561-022-00393-9.

## Introduction

This study investigates whether birthweight matters to quality of life (QOL). Studies of birthweight are classified into those in the domains of medicine and social science, each of which is classified into those investigating the causes and consequences of birthweight. In the field of social sciences, researchers are interested in whether inequality—which is measured by social attributes, such as income, education, and occupation—is passed from generation to generation, and therefore they focused on low birthweight, which might be a link in the transmission of poverty [1]. For example, with regard to the causes, it is known that parents’ education level, income level, and employment types are critical factors [2], while considering the consequences, low birthweight was found to have a negative association with grades of mathematics, as well as with health and employment in adulthood [3].

The present study falls in the social science domain, and investigates the associations of birthweight with QOL in adulthood. Compared with previous studies, this study has a merit of investigating various outcomes including not only academic performance, height, and education in adolescence, but also marital status, body mass index (BMI), income, health, and happiness in adulthood. Among these, the associations with happiness have not been reported in literature. Furthermore, this study is unique as it estimates not only reduced-form for each outcome separately, as done in most previous studies, but also the recursive and structural forms considering the simultaneity of the outcomes. While the reduced form estimates the total association, i.e., the sum of direct and indirect associations, between birthweight and each outcome, the latter reports the direct association between birthweight and each outcome.^1^

The second contribution of this study is that it analyzes data from three distinct countries—Japan, the U.S., and India. Though there have been many studies investigating QOL’s link with birthweight in the U.S., in Japan, there have been no substantial studies except [4, 5]. Furthermore, in India, there have been no studies, to our knowledge, which investigated the associations between birthweight and the long-term socio-economic consequences in adulthood [refer to Additional file 1: Supplementary material A for a detailed review]. As shown in Table 1, the distributions of birthweight in our data are quite different among these countries.^2^ India is one of the countries where the percentage of low-birthweight births is the highest in the world.^3^ In contrast, the share of the high-birthweight births was 15% in the U.S., which was much higher than that of Japan (1.3%) and India (0%). These facts suggest that QOL’s link with low birthweight may differ across countries, and that not only low birthweight, but also high birthweight might be related to QOL. The latter suggestion is consistent with the knowledge in obstetrics that high birthweight is associated with various dysfunctions, such as an increase in frequency of labor dystocia and consequently an increase in Caesarean section rates [8, 9]. High birthweight is caused by maternal obesity and gestational diabetes, which exposes the fetus to elevated levels of fuels such as glucose and fatty acids throughout gestation [10, 11]. Considering that overweight is a major health-related risk factor in adulthood,^4^ high birthweight has a risk of reducing QOL. In this context, the third contribution of this study is to investigate how high birthweight, in addition to low birthweight, influences QOL in adulthood, which has been, to our knowledge, seldom investigated, except for the associations with health.

**Table 1:** Distribution of birthweights

Birthweight (kg)		Japan				U.S.				India	
		Number of observations	Rate			Number of observations	Rate			Number of observations	Rate
			(%)				(%)				(%)
*LBW*	< 2.5	302	6.23	*LBW*	< 2.5	309	6.17	*LBW*	< 2.5	128	12.3
*SBW*	2.5–2.999	1364	28.1	*SBW*	2.5–2.999	1371	27.4	*SBW*	2.5–2.999	266	25.7
	3.0–3.499	1629	33.6		3.0–3.999	1474	29.4		3.0–3.499	209	20.2
	3.5–3.999	307	6.33	*HBW*	4.0–4.499	632	12.6	*Q_HBW*	3.5–3.999	17	1.64
*HBW*	4.0–4.499	58	1.2						4.0–4.499	0	0
	4.5 or more	3	0.06	*V_HBW*	4.5 or more	145	2.89		4.5 or more	0	0
	Do not know	1187	24.5		Do not know	1079	21.5		Do not know	417	40.2
	Total	4850	100			5010	100		1037		100

*SBW* represents standard birthweight

The rest of this study is organized as follows: In the methods section, expositions of outline of survey, definitions of variables, hypotheses of this study, and estimation methods are given. In the results section, the estimation results of the reduced form, those of recursive and structural forms, and those by full information maximum likelihood (FIML) method are presented. In the discussion section, we discuss the innovation of our results and link them to those from previous studies. In the conclusion section, we argue for the uniqueness of this study, mention its limitations, and review possible future work.

## Methods

### Outline of the survey

This study uses the data of Japan, the U.S., and India for 2011 from the Household Panel Survey on Consumer Preferences and Satisfaction (JHPS-CPS) conducted by Osaka University.^5^ Respondents were not asked whether they were singletons or twins in the survey. In Japan, the survey was executed by Central Research Services Inc., a company with extensive experience in academic research and government contracts. Double stratified random sampling from the entire population was used to create a representative sample with respect to sex, age, and living locations. Enumerators visited the homes of the selected respondents to administer the questionnaires. The completed questionnaires were collected after several days. Meanwhile, in the U.S., the survey was conducted by a large survey company, TNS Custom Research, which mailed an English translation of the same questionnaire used in Japan to residents, randomly selected from its pool of registered respondents using the census divisions of the U.S., except for Alaska and Hawaii. In India, Nikkei Research Inc. implemented the survey in six major cities (Delhi, Mumbai, Bangalore, Chennai, Kolkata, and Hyderabad). Each city is separated into four areas; in each area, 15 points were chosen from which five respondents were selected considering their sex, age, and social class (Socio-Economic-Classification; SEC),^6^ and were interviewed because many people with no education were involved. Accordingly, respondents from the poorest to the richest strata are included in the survey. The questions asked in the three countries are nearly the same. Most of the questions in Japanese and English were checked by a bilingual researcher to ensure consistency. In India, the questionnaires in five local languages, Bengali, Hindi, Kannada, Tamil, and Telugu, were used at the interviews in addition to the questionnaire in English.

The number of surveys distributed and collected (response rate), and the observations used for analysis were as follows: Japan, 5316, 4934 (92.8%), 4850; U.S., 7046, 5313 (75.4%), 5075; India, 1280, 1037 (81.0%), 1037.^7^

## Variable definitions^8^

### Birthweight dummies

Respondents were asked how much they weighed when they were born, allowing them to choose from one of the following: “less than 2.5 kg,” “2.5–2.999 kg,” “3.0–3.499 kg,” “3.5–3.999 kg,” “4.0–4.499 kg,” “4.5 kg or more,” and “don’t know”.^9^ The distribution of these responses is shown in Table 1. Compared to 6.2% of the respondents in Japan and the U.S., 12.3% answered “less than 2.5 kg” in India.^10^

We defined low-birthweight dummy (*LBW*), which takes the value of 1 for those who chose “less than 2.5 kg” and 0, otherwise. High-birthweight dummy (*HBW*) in Japan was defined as 1 if birthweight is equal to or more than 4 kg, and 0 otherwise because only three respondents answered “4.5 kg or more” in Japan. In the U.S., 632 respondents between 4 kg and 4.5 kg were defined as *HBW*, and 145 respondents who weighed 4.5 kg or more are defined as very high birthweight (*V_HBW*). In India, since there are no respondents who weighed 4 kg or more, we defined the 17 respondents who weighed 3.5 kg or more as having a “quasi-high birthweight” (*Q_HBW*).^11^ Furthermore, we defined the “do not know dummy” (*DONTKNOW*), which takes 1 for those who chose “don’t know” and 0 otherwise, for all the estimations in the three countries.

### Outcome variables^12^

Academic performance, height, education, and health: Academic performance (*ACADEMIC*) is based on a self-assessment of “grade ranks of all subjects” at the age of 15 years on a five-point scale. *HEIGHT* (in meters) is the height at the time of the survey. *EDUCATION* was defined as the highest level of education completed.^13^

#### Marital status

*MARRIAGE* is a binary dummy variable, which takes 1 for “currently married” and 0 otherwise.

#### BMI

*BMI* was defined as weight (kg) / [height (m)]^2^ at the time of the survey.

#### Personal income

For Japan and the U.S., the respondents were asked to select their personal income (before taxes and including bonuses) in 2010 from a list of 10 options ranging from 1 = “None” to 10= “JPY 14 million (USD 140 thousand) or more.” We estimated the value (unit = JPY 1 million; USD 10 thousand) of each classification by applying lognormal distribution to the frequency distribution of these responses. For India, respondents were asked directly for their monthly personal income (before taxes and including bonuses; unit = INR 10 thousand) in 2010, from which annual personal income was calculated by multiplying with 12. We defined this as “personal income” (*INCOME*). Though personal income of housewives/househusbands, students, and the retired is often extremely low, it is not appropriate to evaluate that their QOL is low. Therefore, this study excluded them from the sample for the estimation of personal income.^14^

*HEALTH* was defined as the responses to the question “How would you describe your current health status?” based on a five-point Likert scale.

#### Happiness

Respondents were asked the question “Overall, how happy would you say you are currently?” and were requested to choose on a scale of 0 to 10, with 0 being “very unhappy” and 10 being “very happy”. We defined the variable *HAPPINESS* with the responses.

### Control variables

**Fig. 1:**
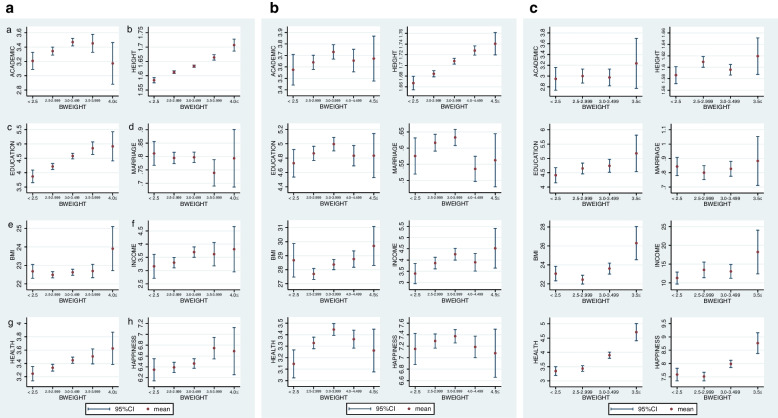
**A** Japan: Each mean outcome variable by birthweight categories with 95% CIs. Notes: This figure implies associations between birthweight and each outcome variable in the case of Japan. *LBW* is lower for all the categories except *MARRIAGE* and *BMI*, while *HBW* is higher for *HEIGHT*, *EDUCATION, BMI, HEALTH,* and *HAPPINESS* in Japan. **B** USA: Each mean outcome variable by birthweight categories with 95% CIs. Notes: This figure implies associations between birthweight and each outcome variable in the case of the U.S. *LBW* is lower for *ACADEMIC*, *HEIGHT*, *EDUCATION*, *INCOME*, *HEALTH* and *HAPPINESS*, while *HBW* is higher for *HEIGHT*, *BMI*, *INCOME* and lower for *MARRIAGE*, and *HAPPINESS* in the U.S. **C** India: Each mean outcome variable by birthweight categories with 95% CIs. Notes: This figure implies associations between birthweight and each outcome variable in the case of India. *LBW* is lower for *HEIGHT*, *EDUCATION*, *INCOME*, *HEALTH* and *HAPPINESS*, while *HBW* is higher for *ACADEMIC*, *HEIGHT*, *EDUCATION*, *BMI*, *INCOME*, *HEALTH*, and *HAPPINESS*

The control variables include parents’ education (*F_EDUCATION*: father’s education, *M_EDUCATION*: mother’s education), parents’ age at birth (*F_AGE_BIRTH*: father’s age at birth, *M_AGE_BIRTH*: mother’s age at birth), standard of living at 15 years old (*S_LIVING*), no siblings at age 15 (*ONLYCHILD*), and mother’s employment status at age 15 (*M_FULLTIME*: full-time work dummy, *M_PARTTIME*: part-time work dummy, and *M_HOUSEWIFE*: housewife dummy).^15^ Other control variables include male dummy (*MALE*), age (*AGE*), age squared (*AGESQ*), depth of religious faith (*RELIGION*), and prefectures and state at the age of 15 years in Japan and the U.S., respectively, and current residence in the six cities in India (region dummies).^16^ The definitions of these variables are summarized in Table 2. Also, Table 3 presents the descriptive statistics of the variables used in the estimation. In Fig. 1, we present the mean and 95% confidence interval (95%CI) of each outcome variables by birthweight categories by country. Looking at the means, Fig. 1A (Japan) reveals that *LBW* is lower for all the categories except *MARRIAGE* and *BMI*, while *HBW* is higher for *HEIGHT*, *EDUCATION*, *BMI*, *HEALTH*, and *HAPPINESS*. Figure 1B (U.S.) reveals that *LBW* is lower for *ACADEMIC*, *HEIGHT*, *EDUCATION*, *INCOME*, *HEALTH*, and *HAPPINESS*, while *HBW* is higher for *HEIGHT*, *BMI*, *INCOME*, and lower for *MARRIAGE* and *HAPPINESS*. Figure 1C (India) reveals that *LBW* is lower for *HEIGHT*, *EDUCATION*, *INCOME*, *HEALTH* and *HAPPINESS*, while *HBW* is higher for *ACADEMIC*, *HEIGHT*, *EDUCATION*, *BMI*, *INCOME*, *HEALT*, and *HAPPINESS*. In sum, the tendency of lower outcomes for *LBW* is recognized in three countries, while *HBW* is associated with lower outcome in the U.S. but higher outcomes in India and Japan. Since 95% CI is large from several cases, however, regression analysis is required to get more reliable results.

**Table 2:** Definition of the variables

Name of the variables	Definition of the variables
Academic performance (*ACADEMIC*)	Self-assessment of “Average of all Subjects” at age 15 on a five-point Likert scale, with 1 being the lowest and 5 being the highest performance.
Height (*HEIGHT*)	Height in meters at the time of the survey.
Education (*EDUCATION*)	Highest level of education completed. For Japan, from 1 = “Grade School” to 11 = “Doctoral Degree.” For the U.S., from 1 = “Grade School” to 9 = “Doctoral Degree.” For India, from 1 = “Illiterate” to 8 = “Graduate/ Post-Graduate-Professional.”
Marital status (*MARRIAGE*)	1 = married, 0 = otherwise
BMI (*BMI*)	Weight in kilograms divided by the square of height in meters (kg/m^2^).
Personal Income (*INCOME*)	Annual personal income (before taxes and including bonuses). JPY 1 million for Japan, USD 10 thousand for the U.S. and INR 10 thousand for India. Housewives/househusbands, students, and retired individuals are excluded. For Japan and the U.S., respondents chose from 1 = “No income” to 10 = to “14 million JPY (140 thousand USD) or more.” The lognormal distribution was applied to the frequency distribution to estimate the class values. For India, monthly income (10 thousand rupees) multiplied by 12.
Health (*HEALTH*)	Self-assessment of health status at the time of response on a five-point Likert scale: from 1 = “not good” to 5 = “good.”
Happiness (*HAPPINESS*)	Self-assessment of happiness at the time of response, from 0 = “very unhappy” to 10 = “very happy.”
Low birthweight (*LBW*)	1 = weighing less than 2.5 kg, 0 = otherwise
High birthweight (*HBW*)	For Japan, 1 = weighing more than 4 kg, 0 = otherwise. For the U.S., 1 = weighing 4–4.499 kg, 0 = otherwise.
Quasi-high birthweight (*Q_HBW*)	For India, 1 = weighing more than 3.5 kg, 0 = otherwise.
Very high birthweight (*V_HBW*)	For the U.S., 1 = weighing more than 4.5 kg, 0 = otherwise.
Do not know (*DONTKNOW*)	1 = do not know the birthweight, 0 = otherwise
Age (*AGE*)	Respondent’s age
Age-squared (*AGESQ*)	Squared term of the respondent’s age
Old age dummy (*OLD*)	1 = the respondent’s age is higher than or equal to 50 years old, 0 = otherwise.
Gender (*MALE*)	1 = male, 0 = female
Father’s education (*F_EDUCATION*)	Education level of the respondent’s father
Mother’s education (*M_EDUCATION*)	Education level of the respondent’s mother
Father’s age at birth (*F_AGE_BIRTH*)	Father’s age when the respondent was born.
Mother’s age at birth (*M_AGE_BIRTH*)	Mother’s age when the respondent was born.
Mothers’ employment status dummy	Mother’s employment status when the respondent was 15 years old. Full-time worker (M_FULLTIME), part-time worker (M_PARTTIME), housewife (M_HOUSEWIFE)
Standard of living at age 15 (*S_LIVING*)	Self-assessment of “Standard of living” at the age of 15 years, on a 11-point Likert scale, from 0 = “lowest” to 10 = “highest.”
Only child dummy (*ONLYCHILD*)	1 = no siblings at age 15, 0 = otherwise
Religious beliefs (*RELIGION*)	Self-assessment of degree of religious beliefs at the time of response, with 1 = “doesn’t hold true at all” to 5 = “particularly true” to the statement “I am deeply religious.”

**Table 3:** Descriptive statistics of the variables

	Japan				U.S.				India			
	Obs.	mean	median	IQR	Obs.	mean	median	IQR	Obs.	mean	median	IQR
*ACADEMIC*	4717	3.39	3.0	1.0	2772	3.69	4.0	2.0	926	3.06	3.0	2.0
*EDUCATION*	4795	4.27	3.0	4.0	3680	5.02	5.0	2.0	1037	4.57	5.0	1.0
*HEIGHT* (m)	4797	1.62	1.62	0.14	3283	1.70	1.70	0.15	1037	1.59	1.6	0.1
*WEIGHT* (kg)	4716	59.9	59.0	15.0	3586	82.2	79.4	24.9	1036	58.5	58	15
*BMI*	4714	22.6	22.3	3.98	3203	28.2	26.8	7.70	1036	23.1	22.8	5.4
*INCOME*	2989	3.54	2.86	3.40	2774	4.16	2.88	3.41	484	11.8	9.6	8.4
*HEALTH*	4802	3.35	3.0	1.0	3678	3.37	3.0	1.0	1037	3.46	3.0	1.0
*HAPPINESS*	4738	6.39	7.0	3.0	3563	7.29	8.0	3.0	1037	7.39	8.0	1.0
*AGE*	4850	52.25	53.0	21.0	3703	52.59	53.0	23.0	1037	45.72	45.0	23.0
*F_EDUCATION*	4589	2.64	2.0	2.0	3624	3.58	3.0	3.0	1037	3.86	4.0	3.0
*M_EDUCATION*	4586	2.26	2.0	2.0	3632	3.56	3.0	1.0	1037	3.24	3.0	3.0
*F_AGE_BIRTH*	4234	31.3	31.0	6.0	3703	30.1	29.0	9.0	–	–	–	–
*M_AGE_BIRTH*	4274	27.7	27.0	6.0	3703	27.0	26.0	9.0	–	–	–	–
*S_LIVING*	4800	4.78	5.0	2.0	2777	4.47	5.0	3.0	1037	5.80	6.0	2.0
*RELIGION*	4841	1.66	1.0	1.0	3610	2.95	3.0	2.0	1037	3.95	4.0	2.0
		**Freq.**	**ratio (%)**	**Cum.**		**Freq.**	**ratio (%)**	**Cum.**		**Freq.**	**ratio (%)**	**Cum.**
*Gender*	*MALE*	2591	46.6	46.6		2257	45	55.4		469	45.2	45.2
	*FEMALE*	2259	53.4	100		2798	55.4	100		568	54.8	100
*OLD age dummy*	*OLD*	2033	41.9	41.9		3047	60.0	60.0		407	39.3	39.3
	*NOT OLD*	2817	58.1	100		2028	40.0	100		630	60.8	100
*Martial status*	*MARRIAGE*	3881	80.2	80.2		3010	60.0	60.0		837	80.7	80.7
	*UNMARRIAGE*	957	19.8	100		2006	40.0	100		200	19.3	100
*Mother’s employment status*	*M_FULLTIME*	1861	39.3	39.3		1957	46.9	46.9		64	6.2	6.17
	*M_PARTTIME*	1171	24.7	64.1		873	20.9	67.9		31	3.0	9.16
	*M_HOUSEWIFE*	1550	32.8	96.8		1177	28.2	96.1		930	89.7	98.8
	*Otherwise*	151	3.19	100		162	3.89	100		12	1.2	100
*Siblings*	*ONLYCHILD*	256	5.45	5.45		283	7.46	7.46		60	5.79	5.79
	*Otherwise*	4442	94.55	100		3512	92.54	100		977	94	100

Obs. is number of observations. Calculations are based on the sample of valid responses to the birth weight question. Birthweight categories are presented in Table 1

### Hypotheses

As mentioned in the Introduction, studies in developed countries have found that low birthweight has a negative association with health and education [also refer to Additional file 1: Supplementary material A]. Therefore, we speculate that *LBW* has an unfavorable association with QOL and examine:

- Hypothesis 1: *LBW* has a negative association with the outcomes concerning QOL, specifically, academic performance, height, highest educational attainment, personal income, health, and happiness.

While several studies that have reported associations between *LBW* and health in young adulthood, relatively few have reported associations of *LBW* with outcomes in adulthood and beyond. Therefore, we posit the following hypothesis.

- Hypothesis 2: The association between *LBW* and outcomes concerning QOL is stronger in youth, and weaker in old age.

Although there have been only a few studies, some indicated that *HBW* causes overweight later in life, resulting in various health problems [12, 14, 15]. Moreover, as pointed out in the Introduction, a high BMI prevails in the U.S. (a developed country), whereas underweight prevails in India, which leads us to the following:

- Hypothesis 3: *HBW* has an association with outcomes concerning QOL. Specifically, it has a negative association in the U.S. and a positive association in India.

### Method of estimation

To test Hypotheses 1 through 3, we regressed each of the eight outcome variables on *LBW* and *HBW* as well as the interaction terms of the old age dummy and the birthweight dummies for each country.^17^ We estimated both reduced form and recursive-structural form.

### Reduced form

We estimated the reduced form based on the literature in this field, regressing each outcome variable over exogenous variables including birthweight dummies. All exogenous variables were used in all the equations without venturing into the issue of identification. The estimates of the reduced form represent the total association of each regressor with each outcome variable as the outcomes of QOL. The formula of the reduced form is given by Eq. (1).

1 $${OUTCOME}_{ik}={\alpha}_k+{\beta}_{k,m}\sum \limits_m{BW}_{im}+{\upgamma}_{k,m}\sum \limits_m{BW}_{im}{OLD}_i+{\delta}_k{OLD}_i+{\varepsilon}_k{X}_i+{e}_{ik},\kern0.36em k=1,\dots, 8$$

Here, *OUTCOME*_*ik*_ is the *k*th outcome of the *i* th individual, *BW*_*im*_ is the *m*th birthweight dummy variable (*LBW* and *HBW*, etc.), *X*_*i*_ is the set of control variables, and *e*_*ik*_ is the disturbance term. If the *m*th birthweight dummy lowers the *k*th outcome, then *β*_*k*, *m*_ will be negative. We also expect that the association of birthweight dummy is stronger for younger people and will gradually disappear as they get older (Hypothesis 2). To measure this change, we created an old age dummy (*OLD*_*i*_), which takes 1 if the respondent’s age is higher than or equal to 50 years old, and 0 otherwise. We made its interaction terms with birthweight dummies. Therefore, the coefficient of birthweight dummies, *β*_*k*, *m*_, represents its association for young respondents, while the coefficient of the interaction terms, *γ*_*k*, *m*_, represents the difference in the associations between the old and the young. For *BMI*, *INCOME*, *HEALTH*, and *HAPPINESS*, *γ*_*k*, *m*_ is expected to take the opposite sign of *β*_*k*, *m*_, because the influence on these outcomes will be smaller for the older respondent. On the other hand, because *ACADEMIC*, *HEIGHT*, *EDUCATION*, and *MARRIAGE* are outcomes corresponding to a younger age for most respondents, we expect that they are independent of their age when the survey was conducted, so that *γ*_*k*, *m*_ is expected to be zero.

As control variables, we used sex and age as basic attributes, parents’ education and parents’ age at birth (as confounding factors), standard of living at the age of 15 years, presence or absence of siblings at the age of 15 years, mother’s employment status at the age of 15 years, region of residence at the age of 15 years, and the depth of religious faith, which we regarded as exogenous to the outcome variables.

We estimate Eq. (1) with ordinary least squares (OLS) for *HEIGHT* and *BMI*, with logit for *MARRIAGE* and with ordered logit for *ACADEMIC*, *EDUCATION*, *INCOME*, *HEALTH*, and *HAPPINESS*.^18^

### Recursive and structural form

**Fig. 2:**
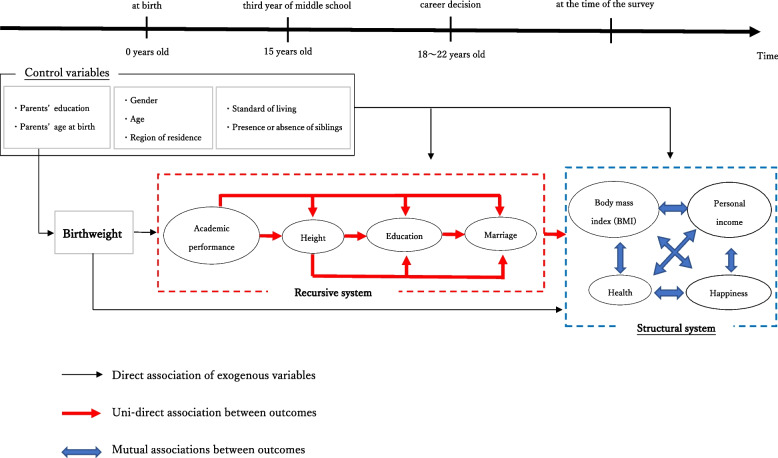
Path diagram: Associations of birthweight on long-term outcomes. Note: This figure shows the timeline path from birth until the survey, meaning that birthweight is associated with each outcome related to quality-of-life (QOL)

Since the eight outcome variables are endogenous, the problem of identification arises. Therefore, in this study, in addition to the reduced form, we estimate the recursive and structural form.^19^ Figure 2 depicts the timeline, or possible paths, from birth until the survey, through which birthweight has a connection with each outcome concerning QOL. As Fig. 2 shows, birthweight is connected with the adult outcomes directly and indirectly.

We assume the recursive system of the outcomes to be occurring in the order of *ACADEMIC*, *HEIGHT*, *EDUCATION*, and *MARRIAGE*.^20^ Therefore, the specification for *ACADEMIC* is the same as in Eq. (1). As for *HEIGHT*, the specification becomes the one which added *ACADEMIC* to Eq. (1). For *EDUCATION*, *HEIGHT* is added to this equation, and for *MARRIAGE*, *EDUCATION* is further added to this equation. Each equation is estimated separately by OLS.

Note that the time-ordering simply implies that predetermined variables are not affected by later events. However, the ordering is not precise, so there should exist some who do not follow it: For example, some people graduate from university after they marry. Therefore, this timeline includes advantages and drawbacks. We should consider the tradeoff between assuming slightly unsound time-ordering and solving the difficult endogeneity problem among eight instead of four outcome variables. This paper chose the former approach in consideration of the fact that the burden of the endogeneity problem is enormous.

Among the assumed orderings, many people may question if higher academic performance will lead to higher height. However, we do not argue this causation, or any causation, in Fig. 2. We just assume that height, which is determined around 18 years of age for many people, does not affect academic performance in 15-year-olds. Many people may also question if height affects performance in later life. Though we again do not assume this causation, there have been studies that report that height is positively associated with wage level and so there is a height premium in the labor market (e.g., [16–18]).

However, simultaneity cannot be denied for the variables, *BMI*, *INCOME*, *HEALTH*, and *HAPPINESS* because the respondents were asked about their current state at the time of the survey. To overcome the simultaneity bias, we estimated the structural form of these four endogenous variables with two-stage least squares (2SLS) using instrument variables. For the robustness check, we also conducted full-information maximum likelihood (FIML) estimation of the whole system of eight outcomes, using the same instrument variables and the same structure as the recursive and structural forms.

We should be aware that the coefficients of the birthweight dummies of the recursive-structural form represent the “direct associations.” Therefore, the estimates should differ from the estimates of the reduced form, which represent the total association including the “indirect association.”

## Results

### Estimation results of the reduced form

In Tables 4, 5 and 6, we report the odds ratio for categorical variables and the OLS estimates for all the outcome variables. We report the OLS estimates even for categorical variables because the marginal effects in ordered regressions are difficult to interpret, and the estimates are better than odds ratios for comparison with those in other Tables.^21^

**Table 4:** The estimates of the reduced form: Japan

	*ACADEMIC*		*HEIGHT*	*EDUCATION*		*MARRIAGE*		*BMI*	*INCOME*		*HEALTH*		*HAPPINESS*	
	*OLS*	*Ordered logit*	*OLS*	*OLS*	*Ordered logit*	*OLS*	*Logit*	*OLS*	*OLS*	*Ordered logit*	*OLS*	*Ordered logit*	*OLS*	*Ordered logit*
	*Coef.*	*Odds Ratio*	*Coef.*	*Coef.*	*Odds Ratio*	*Coef.*	*Odds Ratio*	*Coef.*	*Coef.*	*Odds Ratio*	*Coef.*	*Odds Ratio*	*Coef.*	*Odds Ratio*
*LBW*	−0.197*	0.661**	−0.0280***	− 0.456***	0.599***	0.0569*	1.534*	0.784**	−0.532**	0.699*	−0.251***	0.614***	−0.424**	0.608***
	(0.102)	(0.127)	(0.006)	(0.147)	(0.098)	(0.0334)	(0.397)	(0.320)	(0.219)	(0.143)	(0.0932)	(0.115)	(0.168)	(0.100)
*LBW×OLD*	0.0265	1.126	−0.00053	0.18	1.085	− 0.109**	0.436**	− 0.910**	0.966**	1.854**	0.102	1.171	0.492**	1.686**
	(0.137)	(0.282)	(0.007)	(0.214)	(0.277)	(0.0483)	(0.157)	(0.428)	(0.452)	(0.552)	(0.125)	(0.294)	(0.230)	(0.394)
*HBW*	−0.226	0.674	0.0392***	− 0.211	0.794	0.00927	1.046	1.163**	−0.083	0.869	0.0263	0.973	−0.048	0.944
	(0.164)	(0.198)	(0.010)	(0.324)	(0.263)	(0.0653)	(0.393)	(0.474)	(0.444)	(0.245)	(0.140)	(0.266)	(0.292)	(0.266)
*HBW×OLD*	0.104	1.773	−0.0261	0.489	2.229	0.0928	2.189	−2.043**	−0.774	1.250	−0.323	0.616	0.0546	0.971
	(0.434)	(1.27)	(0.022)	(0.714)	(1.927)	(0.117)	(2.596)	(0.992)	(0.716)	(0.667)	(0.284)	(0.366)	(0.423)	(0.422)
*DONTKNOW*	−0.268***	0.641**	−0.00548	−0.339**	0.687**	−0.0952**	0.559***	−0.181	− 0.162	0.841	− 0.157*	0.711*	− 0.413**	0.650**
	(0.103)	(0.121)	(0.005)	(0.165)	(0.125)	(0.0386)	(0.117)	(0.241)	(0.259)	(0.175)	(0.0916)	(0.132)	(0.163)	(0.111)
*DONTKNOW×OLD*	0.194*	1.418*	−0.00291	0.174	1.169	0.105**	1.978***	0.0837	0.162	1.180	0.0722	1.205	0.352*	1.434*
	(0.114)	(0.295)	(0.005)	(0.185)	(0.243)	(0.0419)	(0.508)	(0.278)	(0.319)	(0.282)	(0.101)	(0.247)	(0.183)	(0.273)
*OLD*	0.0278	1.024	−0.00531	0.293***	1.422***	−0.0970***	0.693**	0.247	0.261	1.144	−0.112*	0.780**	−0.277**	0.766**
	(0.063)	(0.117)	(0.003)	(0.104)	(0.162)	(0.022)	(0.128)	(0.191)	(0.210)	(0.150)	(0.0584)	(0.091)	(0.109)	(0.085)
*MALE*	0.0222	1.062	0.132***	0.749***	2.063***	0.0198	1.170*	1.568***	2.945***	10.908***	−0.0198	0.966	−0.184***	0.832***
	(0.034)	0.065	(0.002)	(0.058)	(0.139)	(0.0122)	(0.108)	(0.102)	(0.107)	(0.943)	(0.0307)	(0.059)	(0.0580)	(0.049)
*AGE*	0.709	1.014	0.288***	5.618***	1.095***	7.188***	1.461***	13.43***	36.42***	1.271***	−3.107***	0.938***	−0.713	1.004
	(1.057)	(0.019)	(0.056)	(1.738)	(0.022)	(0.372)	(0.036)	(3.109)	(3.039)	(0.031)	(0.973)	(0.018)	(1.745)	(0.018)
*AGESQ*	1.140	1.000	−0.386***	−7.595***	0.999***	−6.003***	0.997***	−11.42***	−35.77***	0.998***	2.764***	1.001***	1.977	1.000
	(1.000)	(0.0002)	(0.052)	(1.654)	(0.0002)	(0.350)	(0.0002)	(2.941)	(3.232)	(0.0002)	(0.928)	(0.0002)	(1.660)	(0.0002)
*F_EDUCATION*	0.0581***	1.118***	0.000971*	0.233***	1.279***	−0.00243	0.982	−0.0801***	0.110***	1.078***	0.0204**	1.048**	0.0163	1.035*
	(0.011)	(0.022)	(0.001)	(0.017)	(0.024)	(0.00377)	(0.025)	(0.0300)	(0.0351)	(0.024)	(0.00969)	(0.020)	(0.0186)	(0.019)
*M_EDUCATION*	0.0722***	1.147***	0.00118	0.204***	1.253***	0.00056	1.005	0.0322	0.0879	1.057*	−0.00315	0.992	0.00562	1.001
	(0.017)	(0.033)	(0.001)	(0.027)	(0.038)	(0.00587)	(0.041)	(0.0483)	(0.0544)	(0.034)	(0.0147)	(0.029)	(0.0270)	(0.027)
*F_AGE_BIRTH*	0.334	1.008	0.0164	2.216***	1.026***	−0.315*	0.791*	0.0659	0.173	0.994	0.0685	0.999	−1.169	0.988
	(0.503)	(0.099)	(0.0237)	(0.789)	(0.009)	(0.178)	(0.103)	(1.480)	(1.559)	(0.011)	(0.437)	(0.087)	(0.894)	(0.009)
*M_AGE_BIRTH*	−0.680	0.985	−0.0279	−1.229	0.989	0.0608	1.049	−1.071	0.282	1.005	−0.100	0.993	0.628	1.006
	(0.569)	(0.010)	(0.0277)	(0.906)	(0.010)	(0.200)	(0.155)	(1.727)	(1.829)	(0.012)	(0.505)	(0.100)	(1.023)	(0.011)
*M_FULLTIME*	0.385***	2.039***	0.0119**	0.963***	3.743***	0.0152	1.162	−0.382	0.593	1.754**	−0.0694	0.867	0.302	1.355
	(0.118)	(0.434)	(0.005)	(0.152)	(0.870)	(0.0421)	(0.367)	(0.355)	(0.367)	(0.420)	(0.0978)	(0.172)	(0.211)	(0.288)
*M_PARTTIME*	0.372***	2.005***	0.0116**	0.988***	3.963***	−0.0117	0.947	−0.648*	0.693*	1.703**	−0.0955	0.827	0.341	1.413
	(0.121)	(0.436)	(0.005)	(0.157)	(0.935)	(0.0433)	(0.304)	(0.362)	(0.376)	(0.414)	(0.100)	(0.169)	(0.217)	(0.308)
*M_HOUSEWIFE*	0.453***	2.274***	0.0128**	0.996***	3.957***	−0.0226	0.871	−0.795**	0.709*	1.756	−0.0574	0.891	0.296	1.329
	(0.119)	(0.490)	(0.005)	(0.154)	(0.926)	(0.0424)	(0.275)	(0.356)	(0.378)	(0.427)	(0.0983)	(0.178)	(0.212)	(0.283)
*S_LIVING*	0.0472***	1.080***	0.00102**	0.133***	1.169***	0.00292	1.019	−0.00148	0.0755**	1.023	0.0358***	1.076***	0.156***	1.183***
	(0.011)	(0.021)	(0.001)	(0.0164)	(0.022)	(0.00363)	(0.027)	(0.0311)	(0.0353)	(0.022)	(0.00955)	(0.021)	(0.0186)	(0.023)
*ONLYCHILD*	−0.0136	0.944	−0.00504	0.181	1.266	−0.0497*	0.696	0.373	0.00768	1.023	0.00091	1.008	0.0788	1.050
	(0.072)	(0.123)	(0.004)	(0.127)	(0.185)	(0.0288)	(0.130)	(0.237)	(0.258)	(0.150)	(0.0689)	(0.141)	(0.121)	(0.136)
*RELIGION*	−0.0441***	0.929**	−0.0012	− 0.0523*	0.951	0.00027	1.005	0.042	−0.109**	0.953	−0.0464***	0.913***	0.0384	1.048
	(0.017)	(0.028)	(0.001)	(0.028)	(0.031)	(0.00566)	(0.045)	(0.0479)	(0.0511)	(0.034)	(0.0160)	(0.030)	(0.0302)	(0.034)
Cons	2.054***		1.504***	0.101		−1.093***		19.45***	−8.732***		4.151***		5.529***	
	(0.328)		(0.0172)	(0.531)		(0.118)		(0.978)	(0.958)		(0.297)		(0.549)	
Obs.	3814	3814	3839	3845	3845	3860	3860	3776	2742	2742	3837	3837	3791	3791
R^2^	0.091		0.642	0.269		0.164		0.104	0.27		0.056		0.053	

The coefficients are estimated by OLS and odds rations by orderd logit. The definition and unit of each variable are presented in Table 2. Regression coefficients are presented in the upper rows. The region dummies representing the prefecture where respondents lived at the age of 15 years are included in the estimation, but are not shown here to save space. Robust standard errors are in parentheses. Because the estimated coefficients are very small, the coefficients and standard errors for *AGE*, *F_AGE_BIRTH*, and *M_AGE_BIRTH* are multiplied by 100 and *AGESQ* by 1000. In the case of linear regression, if the units of explanatory variables are changed, such as when dividing by 100, the estimated coefficients and standard errors are multiplied by 100 while the t-values remain the same. *** *p* < 0.01, ** *p* < 0.05, * *p* < 0.1

**Table 5:** The estimates of the reduced form: U.S.

	*ACADEMIC*		*HEIGHT*	*EDUCATION*		*MARRIAGE*		*BMI*	*INCOME*		*HEALTH*		*HAPPINESS*	
	*OLS*	*Ordered logit*	*OLS*	*OLS*	*Ordered logit*	*OLS*	*Logit*	*OLS*	*OLS*	*Ordered logit*	*OLS*	*Ordered logit*	*OLS*	*Ordered logit*
	*Coef.*	*Odds Ratio*	*Coef.*	*Coef.*	*Odds Ratio*	*Coef.*	*Odds Ratio*	*Coef.*	*Coef.*	*Odds Ratio*	*Coef.*	*Odds Ratio*	*Coef.*	*Odds Ratio*
*LBW*	−0.0273	0.847	− 0.00955	− 0.127	1.054	− 0.038	0.835	−1.418	− 0.299	0.888	0.126	1.210	0.406	1.379
	(0.158)	(0.271)	(0.0115)	(0.208)	(0.221)	(0.0720)	(0.301)	(1.213)	(0.478)	(0.238)	(0.126)	(0.333)	(0.316)	(0.390)
*LBW×OLD*	−0.149	0.837	0.0148	0.0862	0.904	0.0576	1.327	1.933	−0.574	0.732	− 0.281	0.691	−0.384	0.745
	(0.194)	(0.317)	(0.0154)	(0.293)	(0.279)	(0.0896)	(0.610)	(1.507)	(0.668)	(0.286)	(0.174)	(0.251)	(0.422)	(0.272)
*HBW*	0.0398	1.075	0.0187***	0.0408	1.028	−0.105***	0.593***	1.260*	−0.165	0.911	−0.0638	0.866	−0.151	0.860
	(0.0911)	(0.189)	(0.00657)	(0.144)	(0.154)	(0.0392)	(0.119)	(0.708)	(0.357)	(0.161)	(0.0808)	(0.144)	(0.201)	(0.139)
*HBW×OLD*	−0.142	0.811	0.000225	−0.138	0.837	0.0595	1.344	−0.293	−0.743	0.707	0.0259	1.110	−0.141	0.823
	(0.131)	(0.198)	(0.00975)	(0.222)	(0.202)	(0.0595)	(0.400)	(0.980)	(0.527)	(0.197)	(0.120)	(0.268)	(0.295)	(0.196)
*V_HBW*	−0.00792	1.012	0.0658***	−0.22	0.762	0.028	1.163	3.072**	−0.54	0.956	−0.419***	0.391***	−0.894**	0.534**
	(0.190)	(0.333)	(0.0179)	(0.310)	(0.261)	(0.0798)	(0.464)	(1.507)	(0.536)	(0.262)	(0.158)	(0.116)	(0.455)	(0.166)
*V_HBW×OLD*	0.098	1.145	−0.0485	0.588	2.127	−0.139	0.508	−1.757	1.402	1.398	0.505*	3.474**	1.162**	2.051*
	(0.275)	(0.581)	(0.0296)	(0.456)	(1.075)	(0.123)	(0.288)	(2.261)	(1.073)	(0.700)	(0.260)	(1.904)	(0.573)	(0.853)
*DONTKNOW*	0.0637	1.164	−0.00629	0.149	1.235	−0.0114	0.949	−0.267	0.717*	1.314	−0.186**	0.631***	−0.179	0.794
	(0.106)	(0.217)	(0.00772)	(0.160)	(0.210)	(0.0446)	(0.213)	(0.696)	(0.432)	(0.233)	(0.0861)	(0.110)	(0.202)	(0.123)
*DONTKNOW×OLD*	−0.0319	0.946	0.00319	0.017	0.955	−0.0557	0.760	0.646	−1.115**	0.652*	0.17	1.542**	0.113	1.088
	(0.131)	(0.223)	(0.00924)	(0.203)	(0.208)	(0.0563)	(0.209)	(0.867)	(0.553)	(0.159)	(0.109)	(0.340)	(0.256)	(0.224)
*OLD*	0.0385	1.055	−0.00135	0.0399	1.109	−0.0965**	0.614**	−0.102	− 0.0396	1.002	0.00384	1.024	−0.0023	1.029
	(0.0889)	(0.174)	(0.00624)	(0.136)	(0.161)	(0.0375)	(0.123)	(0.597)	(0.358)	(0.172)	(0.0779)	(0.160)	(0.195)	(0.160)
*MALE*	−0.196***	0.745***	0.138***	0.0976	1.060	0.0321	1.173	0.353	1.931***	2.508***	0.0487	1.112	0.178*	1.110
	(0.0461)	(0.064)	(0.00338)	(0.0751)	(0.087)	(0.0202)	(0.119)	(0.310)	(0.186)	(0.232)	(0.0412)	(0.093)	(0.0979)	(0.090)
*AGE*	−0.244	0.993	0.0479	5.554***	1.061***	5.798***	1.315***	34.23***	40.27***	1.254***	−2.497***	0.945***	−4.375**	0.971**
	(0.894)	(0.017)	(0.0652)	(1.365)	(0.016)	(0.363)	(0.027)	(6.185)	(2.960)	(0.021)	(0.774)	(0.015)	(1.807)	(0.015)
*AGESQ*	0.528	1.000	−0.0659	−4.104***	1.000***	−4.949***	0.998***	−34.04***	−36.00***	0.998***	1.313*	1.000**	5.137***	1.000***
	(0.780)	(0.0001)	(0.0555)	(1.244)	(0.0001)	(0.332)	(0.0002)	(5.068)	(2.718)	(0.0002)	(0.675)	(0.0001)	(1.551)	(0.0001)
*F_EDUCATION*	0.0448***	1.084***	0.000901	0.217***	1.269***	0.00546	1.030	−0.348***	0.315***	1.126***	0.0417***	1.082***	0.0395	1.036
	(0.0146)	(0.030)	(0.00108)	(0.0231)	(0.032)	(0.00627)	(0.033)	(0.0983)	(0.0659)	(0.033)	(0.0124)	(0.027)	(0.0326)	(0.027)
*M_EDUCATION*	0.0785***	1.171***	0.00337***	0.181***	1.219***	−0.0184**	0.909***	−0.167	0.0353	1.022	0.0277*	1.056*	−0.0104	0.973
	(0.0169)	(0.038)	(0.00123)	(0.0276)	(0.036)	(0.00728)	(0.033)	(0.110)	(0.0693)	(0.032)	(0.0149)	(0.033)	(0.0361)	(0.028)
*F_AGE_BIRTH*	−0.371	0.991	−0.0264	−0.100	0.999	−0.439*	0.978*	1.742	1.299	0.999	−0.446	0.990	0.261	0.997
	(0.525)	(0.010)	(0.0387)	(0.859)	(0.009)	(0.241)	(0.011)	(3.699)	(2.054)	(0.011)	(0.457)	(0.009)	(1.093)	(0.009)
*M_AGE_BIRTH*	0.695	1.015	0.0193	2.815***	1.030***	0.131	1.007	−3.578	0.843	1.011	1.087**	1.024**	1.227	1.014
	(0.614)	(0.012)	(0.0460)	(0.987)	(0.011)	(0.272)	(0.013)	(4.290)	(2.275)	(0.012)	(0.541)	(0.011)	(1.316)	(0.011)
*M_FULLTIME*	0.177	1.433	−0.0154	0.222	1.277	0.0774	1.474	0.023	1.230***	1.701*	0.195	1.587*	−0.335	0.664
	(0.166)	(0.465)	(0.0111)	(0.244)	(0.347)	(0.0660)	(0.462)	(1.333)	(0.442)	(0.479)	(0.135)	(0.399)	(0.359)	(0.193)
*M_PARTTIME*	0.304*	1.820*	−0.00642	0.475*	1.671*	0.0512	1.275	−0.709	1.616***	1.884**	0.265*	1.793**	−0.198	0.723
	(0.169)	(0.601)	(0.0113)	(0.250)	(0.466)	(0.0675)	(0.409)	(1.349)	(0.472)	(0.547)	(0.138)	(0.461)	(0.367)	(0.214)
*M_HOUSEWIFE*	0.253	1.664	−0.0138	0.348	1.469	0.0762	1.462	−0.375	1.261***	1.614*	0.21	1.599*	−0.17	0.719
	(0.168)	(0.544)	(0.0113)	(0.248)	(0.404)	(0.0669)	(0.464)	(1.358)	(0.457)	(0.467)	(0.138)	(0.410)	(0.364)	(0.211)
*S_LIVING*	0.0306**	1.042*	0.00176**	0.0464**	1.055**	0.00062	1.003	−0.228**	0.0217	1.038	0.0333***	1.070***	0.0680**	1.062**
	(0.0126)	(0.024)	#########	(0.0197)	(0.023)	(0.00559)	(0.028)	(0.0892)	(0.0463)	(0.026)	(0.0117)	(0.026)	(0.0288)	(0.026)
*ONLYCHILD*	0.0918	1.185	0.00128	0.215	1.208	−0.0775*	0.688*	0.942	−0.285	0.908	−0.114	0.777	−0.281	0.847
	(0.0833)	(0.182)	(0.00616)	(0.145)	(0.190)	(0.0408)	(0.135)	(0.638)	(0.369)	(0.166)	(0.0779)	(0.124)	(0.201)	(0.139)
*RELIGION*	0.0293*	1.055	0.00196	0.0603**	1.062*	0.0375***	1.207	0.0382	−0.0666	0.961	0.0400***	1.080**	0.191***	1.192***
	(0.0177)	(0.035)	(0.00125)	(0.0281)	(0.033)	(0.00754)	(0.046)	(0.120)	(0.0706)	(0.035)	(0.0154)	(0.034)	(0.0375)	(0.038)
Cons	2.784***		1.624***	0.457		−0.914***		24.15***	−9.883***		3.521***		7.015***	
	(0.341)		(0.0238)	(0.517)		(0.140)		(2.406)	(1.125)		(0.299)		(0.706)	
Obs.	2109	2109	1938	2156	2156	2144	2144	1892	1676	1676	2166	2166	2090	2090
R^2^	0.075		0.513	0.176		0.17		0.078	0.198		0.117		0.055	

The coefficients are estimated by OLS and odds rations by orderd logit. The definition and unit of each variable are presented in Table 2. Regression coefficients are presented in the upper rows. The region dummies representing the prefecture where respondents lived at the age of 15 years are included in the estimation, but are not shown here to save space. Robust standard errors are in parentheses. Because the estimated coefficients are very small, the coefficients and standard errors for *AGE*, *F_AGE_BIRTH*, and *M_AGE_BIRTH* are multiplied by 100 and *AGESQ* by 1000. In the case of linear regression, if the units of explanatory variables are changed, such as when dividing by 100, the estimated coefficients and standard errors are multiplied by 100 while the t-values remain the same. *** *p* < 0.01, ** *p* < 0.05, * *p* < 0.1

**Table 6:** The estimates of the reduced form: India

	*ACADEMIC*		*HEIGHT*	*EDUCATION*		*MARRIAGE*		*BMI*	*INCOME*		*HEALTH*		*HAPPINESS*	
	*OLS*	*Ordered logit*	*OLS*	*OLS*	*Ordered logit*	*OLS*	*Logit*	*OLS*	*OLS*	*Ordered logit*	*OLS*	*Ordered logit*	*OLS*	*Ordered logit*
	*Coef.*	*Odds Ratio*	*Coef.*	*Coef.*	*Odds Ratio*	*Coef.*	*Odds Ratio*	*Coef.*	*Coef.*	*Odds Ratio*	*Coef.*	*Odds Ratio*	*Coef.*	*Odds Ratio*
*LBW*	−0.142	0.754	−0.0172*	− 0.513***	0.569***	0.0889**	2.281*	−0.683	−3.428**	0.709	−0.200**	0.605**	−0.158	0.868
	(0.128)	(0.203)	(0.0101)	(0.153)	(0.104)	(0.0439)	(1.006)	(0.518)	(1.346)	(0.223)	(0.0990)	(0.146)	(0.148)	(0.197)
*LBW×OLD*	0.153	1.460	0.00789	0.399	1.604	−0.128*	0.325*	1.793**	0.181	0.743	−0.11	0.806	−0.0637	0.768
	(0.228)	(0.684)	(0.0160)	(0.294)	(0.555)	(0.0742)	(0.193)	(0.872)	(2.746)	(0.443)	(0.172)	(0.350)	(0.244)	(0.274)
*Q_HBW*	0.428	2.377*	0.0143	−0.204	0.788	0.0335	1.593	2.967**	0.306	1.311	0.688***	10.355***	0.423**	2.655*
	(0.270)	(1.075)	(0.0219)	(0.339)	(0.366)	(0.0800)	(1.171)	(1.219)	(2.966)	(0.949)	(0.155)	(7.630)	(0.213)	(1.335)
*Q_HBW×OLD*	−0.311	0.477	−0.0271	0.645	1.972	0.0577	1.0	−0.194	7.228**	4.504*	−0.0791	0.837	0.0463	1.132
	(0.467)	(0.441)	(0.0358)	(0.522)	(1.350)	(0.0854)	(omitted)	(1.629)	(3.446)	(3.515)	(0.294)	(1.223)	(0.240)	(0.602)
*DONTKNOW*	−0.246**	0.587***	−0.0123*	− 0.251*	0.780	− 0.00881	0.953	0.172	−2.189*	0.771	−0.155**	0.676**	−0.433***	0.524***
	(0.0958)	(0.114)	(0.00689)	(0.143)	(0.139)	(0.0369)	(0.281)	(0.414)	(1.156)	(0.182)	(0.0758)	(0.130)	(0.115)	(0.091)
*DONTKNOW×OLD*	0.178	1.386	0.0118	0.634***	2.241***	−0.04	0.733	1.493***	3.700**	2.031*	−0.364***	0.440***	−0.0676	0.925
	(0.142)	(0.390)	(0.00923)	(0.220)	(0.615)	(0.0512)	(0.279)	(0.563)	(1.716)	(0.825)	(0.109)	(0.121)	(0.170)	(0.249)
*OLD*	−0.181	0.717	0.00165	−0.416*	0.602*	−0.107**	0.281**	−1.047*	−1.872	0.7	0.122	1.362	−0.0909	0.729
	(0.154)	(0.222)	(0.0105)	(0.220)	(0.157)	(0.0518)	(0.150)	(0.550)	(1.996)	(0.278)	(0.116)	(0.409)	(0.172)	(0.199)
*MALE*	0.130**	1.294	0.0562***	0.684***	2.081***	0.00665	1.056	−0.163	4.942***	5.601***	0.0301	1.046	0.0354	1.089
	(0.0625)	(0.161)	(0.00461)	(0.0953)	(0.245)	(0.0228)	(0.188)	(0.259)	(0.973)	(1.693)	(0.0491)	(0.131)	(0.0742)	(0.129)
*AGE*	−3.092*	0.947*	−0.125	−4.460*	0.940**	7.138***	1.607***	35.51***	80.78***	1.231***	−2.291*	0.938*	−0.0867	0.993
	(1.640)	(0.031)	(0.118)	(2.468)	(0.028)	(0.678)	(0.077)	(6.918)	(19.47)	(0.060)	(1.350)	(0.033)	(2.124)	(0.032)
*AGESQ*	3.297*	1.001	0.123	2.191	1.000	−6.876***	0.996***	−34.94***	−82.07***	0.998***	1.505	1.000	0.0743	1.000
	(1.908)	(0.0004)	(0.129)	(2.917)	(0.0004)	(0.754)	(0.0005)	(7.722)	(23.84)	(0.001)	(1.565)	(0.0004)	(2.510)	(0.0004)
*F_EDUCATION*	0.0713***	1.134	0.00121	0.161***	1.245***	−0.00743	0.938	0.112	1.557***	1.280***	0.0647***	1.197***	0.0325	1.036
	(0.0257)	(0.058)	(0.00206)	(0.0363)	(0.055)	(0.00887)	(0.068)	(0.134)	(0.419)	(0.108)	(0.0223)	(0.071)	(0.0339)	(0.060)
*M_EDUCATION*	0.0022	1.012	−0.00052	0.0241	1.030	0.00245	1.024	0.0545	−0.0369	1.074	0.00588	0.996	0.0541	1.079
	(0.0275)	(0.056)	(0.00215)	(0.0386)	(0.048)	(0.00892)	(0.076)	(0.140)	(0.336)	(0.091)	(0.0226)	(0.059)	(0.0337)	(0.062)
*M_FULLTIME*	−0.266	0.460	0.00834	0.616	1.913	0.0625	1.751	0.711	−2.492	0.482	0.223	2.098	0.921	2.503
	(0.413)	(0.337)	(0.0160)	(0.493)	(1.142)	(0.124)	(1.379)	(0.922)	(3.881)	(0.326)	(0.302)	(1.760)	(0.576)	(1.793)
*M_PARTTIME*	−0.188	0.635	−0.00505	0.576	1.848	0.0715	1.563	0.149	−3.358	0.639	0.475	3.988	0.547	1.859
	(0.452)	(0.536)	(0.0185)	(0.505)	(1.133)	(0.136)	(1.305)	(1.012)	(3.937)	(0.475)	(0.318)	(3.547)	(0.616)	(1.446)
*M_HOUSEWIFE*	−0.403	0.374	0.0116	0.712	2.257	0.0941	2.016	−0.617	−2.979	0.455	0.623**	5.512**	1.021*	3.348*
	(0.380)	(0.247)	(0.0136)	(0.443)	(1.201)	(0.117)	(1.399)	(0.792)	(3.653)	(0.291)	(0.285)	(4.440)	(0.551)	(2.263)
*S_LIVING*	0.0379**	1.077	0.00254**	0.227***	1.305***	0.0123**	1.103**	−0.0435	0.266	1.087**	0.0206*	1.057*	0.0216	1.044
	(0.0164)	(0.036)	(0.00111)	(0.0230)	(0.038)	(0.00565)	(0.048)	(0.0641)	(0.167)	(0.040)	(0.0120)	(0.033)	(0.0180)	(0.029)
*ONLYCHILD*	−0.0539	0.950	0.00704	0.268	1.433	−0.0737	0.646	−0.581	0.351	1.402	0.0343	1.123	0.284**	1.407
	(0.133)	(0.256)	(0.0111)	(0.202)	(0.340)	(0.0490)	(0.207)	(0.561)	(1.427)	(0.412)	(0.111)	(0.312)	(0.135)	(0.338)
*RELIGION*	−0.105***	0.808	−0.00442*	−0.0351	0.957	0.00719	1.045	−0.164	−0.645	0.925	0.109***	1.331***	0.193***	1.379***
	(0.0316)	(0.052)	(0.00246)	(0.0472)	(0.054)	(0.0111)	(0.091)	(0.122)	(0.465)	(0.083)	(0.0251)	(0.086)	(0.0357)	(0.081)
Cons	4.608***		1.581***	3.329***		−0.951***		16.32***	−9.509*		2.990***		5.862***	
	(0.551)		(0.0311)	(0.743)		(0.201)		(1.891)	(5.585)		(0.431)		(0.748)	
Obs.	926	926	1037	1037	1037	1037	1037	1036	484	484	1037	1037	1037	1037
R^2^	0.175		0.182	0.247		0.185		0.093	0.271		0.294		0.392	

The coefficients are estimated by OLS and odds rations by orderd logit. The definition and unit of each variable are presented in Table 2. Regression coefficients are presented in the upper rows. The region dummies representing the prefecture where respondents lived at the age of 15 years are included in the estimation, but are not shown here to save space. Robust standard errors are in parentheses. Because the estimated coefficients are very small, the coefficients and standard errors for AGE are multiplied by 100 and AGESQ by 1000. In the case of linear regression, if the units of explanatory variables are changed, such as when dividing by 100, the estimated coefficients and standard errors are multiplied by 100 while the t-values remain the same. *** *p* < 0.01, ** *p* < 0.05, * *p* < 0.1

#### Japan

We present the estimates of the reduced form (Eq. (1)) for Japan in Table 4. *LBW* was associated with all the outcomes significantly at the 1% or 5% level. It was negative for *ACADEMIC*, *HEIGHT*, *INCOME*, *HEALTH*, and *HAPPINESS*, while positive for *MARRIAGE* and *BMI*. Though previous studies in Japan reported that low birthweight is negatively associated with adolescent academic performance and health in adulthood, our results indicate that low birthweight was associated with more outcomes. The results that *LBW* was associated with *BMI* positively is consistent with the results of [21]. Finally, the result that *LBW* was negatively associated with happiness is a novel finding of this study. These results support Hypothesis 1.

The interaction terms between *LBW* and old age dummy showed opposite signs to those on *LBW* dummies for all the outcomes except *HEIGHT*, indicating that the associations of *LBW* were weaker for older respondents. However, for *ACADEMIC*, *HEIGHT*, *EDUCATION*, and *HEALTH*, the coefficients of the interaction terms were much smaller than those of *LBW*, indicating that the associations for older and younger respondents were not largely different. On the other hand, for *MARRIAGE*, *BMI*, *INCOME*, and *HAPPINESS*, the absolute values of the coefficients of the interaction terms were comparable with those of *LBW*, suggesting that the association for older adults is small. Indeed, except for academic performance and height, the associations for the elderly were insignificant. These results were consistent with Hypothesis 2. However, health was the exception, on which low birthweight continued to have an association with older adults significantly, which is consistent with previous studies in Japan.

The coefficients of *HBW* were not significant except for *HEIGHT* and *BMI*, while the interaction terms were significantly negative only for *BMI*. These results imply that the associations with *HEIGHT* continued to have an association with older adults, whereas the association with *BMI* was recognized only for younger respondents.

The results are summarized as follows: (1) In Japan, *LBW* was associated with all the outcomes examined in this study for respondents under 50 years old, while the associations were mitigated for those over 50 years old; (2) In Japan, *HBW* had a positive association only with *HEIGHT* and *BMI*.^22^ These conclusions are confirmed with the odds ratio (OR) estimated with an ordered logit model, though there were three differences in the significance level.

In addition to the association based on significance of the coefficients, we also evaluated economic importance using standardized regression (Estimates are presented in Additional file 3: Supplemental material C; Table C-1). Age and gender exhibited the largest associations with many outcomes: the association with *LBW* was merely one tenth to one fourth. Though low birthweight has a smaller association with education than that of father’s education and living standards in childhood, it has a comparable association with personal income and health.

#### The U.S.

In Table 5, we present the estimates of Eq. (1) for the U.S. *LBW* was associated with no outcomes. The magnitudes of the coefficients of *LBW* for various outcomes were less than half of the corresponding estimates in Japan. In addition, the interaction term of *LBW* and old age dummy was not significant. In contrast, *HBW* had a significantly positive association with *HEIGHT* and *BMI*, and a significantly negative association with *MARRIAGE*. *V_HBW* had a significant and positive association with *HEIGHT* and *BMI*, while it had a significantly negative association with *HEALTH* and *HAPPINESS*. Further, as for the outcomes significantly associated with *HBW* and/or *V_HBW*, the interaction terms with old age dummy took the opposite signs, indicating that these associations are mitigated for older adults.

The above results are summarized as follows: (3) in the U.S., though *LBW* was not associated with any outcomes significantly, *HBW* had a negative association with *HEALTH* and *HAPPINESS*, and (4) the significant associations of *HBW* for those under 50 years old were reduced for older adults. Though these results were not consistent with Hypothesis 1, they support Hypotheses 2 and 3.^23^

These conclusions are confirmed with odds ratio estimated with an ordered logit model though there were two differences in the significance level.

According to the standardized regression, the magnitude of the coefficient of *V_HBW* in the equation for *HEALTH* and *HAPPINESS* was around − 0.06, which is tantamount to one sixth of that on age. It was comparable with that of *S_LIVING* for *HEALTH* and *HAPPINESS*, and *F_EDUCATION* and *M_AGE_BIRTH* for *HEALTH*, revealing that the association was fairly large. (Estimates are presented in Additional file 3: Supplemental material C; Table C-2).

#### India

The estimates for India are presented in Table 6, which are summarized as follows: (5) *LBW* is negatively associated with *HEIGHT*, *EDUCATION*, *INCOME*, and *HEALTH*, and positively associated with *MARRIAGE*. (6) As for these outcomes, the coefficients of interaction dummies with the old age dummy took the opposite signs to those on *LBW* except for *HEALTH*, indicating that the associations with these outcomes were mitigated for older adults. However, as for *HEALTH*, the negative association of *LBW* was augmented for those over 50 years old. (7) *Q_HBW* was significantly positive for *BMI*, *HEALTH*, and *HAPPINESS*.^24^

These conclusions are confirmed with the odds ratio estimated with an ordered logit model except for the negative association between *LBW* and *INCOME*, though there were three differences in the significance level.

The standardized regression analysis revealed that the magnitude of the coefficient of *LBW* for *EDUCATION*, *INCOME*, and *HEALTH* were about half to one-third of that of *F_EDUCATION* for these outcomes. The coefficient of *Q_HBW* for *HEALTH* was about two-thirds of that on *F_EDUCATION* and half of that on *M_HOUSEWIFE*. (Estimates are presented in Additional file 3: Supplemental material C; Table C-3).

Comparing the results of the reduced form estimations across the three countries, while *LBW* was associated with various outcomes negatively in Japan and India, it was not associated with any outcome in the U.S. However, in the U.S., *HBW* was negatively and significantly associated with *MARRIAGE*, and *V_HBW* was negatively and significantly associated with *HEALTH* and *HAPPINESS*. In contrast, in India, *Q_HBW* was positively and significantly associated with *HEALTH* and *HAPPINESS*. These different associations of *HBW* in the U.S. and India support Hypothesis 3.

### Estimation results of recursive and structural form

We estimated the recursive system consisting of consisting of *ACADEMIC*, *HEIGHT*, *EDUCATION*, and *MARRIAGE* using OLS, while we estimated the structural form using *BMI*, *INCOME*, *HEALTH*, and *HAPPINESS*, considering that these four variables are endogenous. Specifically, we adopted the averages of the endogenous outcomes across the samples of the same gender, age group, and residence as the instruments.^25^ Though we included all the control variables in all the estimations, they are not shown in the tables to save space.

#### Japan

The estimation results for Japan are presented in Table 7.^26^*LBW* had a significantly negative association with *HEIGHT* and *EDUCATION*, and a significantly positive association with *MARRIAGE* and *BMI*. Though all coefficients had the same signs as those of the reduced form, their absolute values were smaller, except for *HEIGHT* and *BMI*. These results suggest that the indirect associations have the same signs as direct associations, except *HEIGHT* and *BMI*. Meanwhile, *INCOME*, *HEALTH*, and *HAPPINESS*, whose coefficients were significantly negative in the reduced form estimations, became insignificant in the structural estimations, indicating that *LBW* was associated with these outcomes through indirect paths, but not directly.

**Table 7:** The estimates of the recursive and structural forms: Japan

	Recursive			Structural			
	*HEIGHT*	*EDUCATION*	*MARRIAGE*	*BMI*	*INCOME*	*HEALTH*	*HAPPINESS*
*LBW*	−0.0282***	− 0.324**	0.0685**	0.689*	−0.401*	− 0.0672	− 0.196
	(0.00563)	(0.149)	(0.0333)	(0.389)	(0.243)	(0.109)	(0.201)
*LBW×OLD*	0.000661	0.176	−0.114**	− 0.922*	0.978*	−0.0428	0.506*
	(0.00738)	(0.206)	(0.0487)	(0.534)	(0.515)	(0.158)	(0.281)
*HBW*	0.0390***	−0.142	0.0217	1.146**	−0.178	0.0354	0.084
	(0.0104)	(0.308)	(0.0642)	(0.511)	(0.470)	(0.141)	(0.281)
*HBW×OLD*	−0.0219	0.652	0.0458	−2.286***	−0.984	−0.348	0.496
	(0.0236)	(0.630)	(0.132)	(0.803)	(0.836)	(0.303)	(0.463)
*DONTKNOW*	−0.00418	−0.166	− 0.0932**	− 0.526*	0.158	− 0.0802	−0.228
	(0.00484)	(0.154)	(0.0395)	(0.282)	(0.268)	(0.0999)	(0.165)
*DONTKNOW×OLD*	−0.00369	0.0607	0.103**	0.28	−0.104	−0.0015	0.256
	(0.00542)	(0.174)	(0.0429)	(0.329)	(0.320)	(0.112)	(0.188)
*OLD*	−0.00617*	0.281***	−0.0953***	− 0.0372	0.271	− 0.0724	− 0.139
	(0.00333)	(0.0958)	(0.0223)	(0.227)	(0.214)	(0.0661)	(0.120)
*ACADEMIC*	0.00294***	0.643***	0.0168**	−0.0432	0.313***	0.0455*	0.0927**
	#########	(0.0271)	(0.00677)	(0.0828)	(0.0725)	(0.0235)	(0.0416)
*HEIGHT*		0.717	0.148	−1.78	2.260**	−0.221	0.631
		(0.493)	(0.118)	(1.182)	(1.055)	(0.346)	(0.600)
*EDUCATION*			−0.00133	0.0178	0.267***	0.00878	0.0135
			(0.00386)	(0.0451)	(0.0409)	(0.0140)	(0.0245)
*MARRIAGE*				0.124	0.234	0.146*	0.768***
				(0.287)	(0.235)	(0.0812)	(0.117)
*BMI*					0.107	−0.0001	0.000208
					(0.102)	(0.0370)	(0.0619)
*INCOME*				0.0612		−0.0372	0.126**
				(0.0953)		(0.0286)	(0.0497)
*HEALTH*				−0.0583	−0.761**		0.704***
				(0.488)	(0.368)		(0.223)
*HAPPINESS*				−0.14	0.359**	0.185***	
				(0.213)	(0.169)	(0.0623)	
Cons	1.496***	−2.222**	−1.358***	24.18***	−13.21***	3.431***	3.263
	(0.0173)	(0.897)	(0.213)	(3.347)	(3.447)	(1.160)	(2.188)
Obs.	3789	3771	3765	2587	2587	2587	2587
R^2^	0.641	0.376	0.167	0.121	0.278	0.163	0.229
Weak identification test			F(3, 2515)	18.569	17.691	21.232	17.261
*Kleibergen-Paap Wald rk F statistic*							
Underidentification test			χ^2^(3)	46.338***	44.916***	59.690***	44.982***
*Kleibergen-Paap rk LM statistic*							
*F*-value of the first stage			BMI	–	20.56***	19.97***	20.57***
			Income	41.30***	–	41.44***	40.77***
			Health	18.95***	18.94***	–	18.94***
			HAPPINESS	23.26***	22.60***	23.16***	–

The coefficients are estimated by OLS. The definition and unit of each variable are presented in Table 2. Regression coefficients are presented in the upper rows. All control variables are included in the estimation, but their estimates are not shown in the table to save space. The recursive system is estimated with OLS, while structural system is estimated with 2SLS using instrumental variables: the average value of each variable (BMI, income, health, and happiness) with the same three attributes of gender, age, and place of present residence. Robust standard errors are in parentheses. *** *p* < 0.01, ** *p* < 0.05, * *p* < 0.1

*HBW* was significantly positive for *HEIGHT* and *BMI*, but insignificant for other outcomes. In addition, the magnitudes of the significant coefficients were similar to the estimates of the reduced form, suggesting that the indirect associations of *HBW* were marginal. In sum, these results are consistent with those of the reduced form. The results concerning Hypothesis 2 (i.e., interaction terms of old age and birthweight dummies) by the recursive-structural form are consistent with those of the reduced form.^27^

#### The U.S.

We present the estimation results for the U.S. in Table 8. Estimates of *LBW* were not significant for any outcomes, as were in the reduced form. Coefficients of *HBW* were significantly positive for *HEIGHT* and significantly negative for *MARRIAGE*, as in the reduced form, whereas it was insignificant for *BMI*. In addition, the absolute values of the estimates became smaller than those of the reduced form representing the total associations, suggesting that indirect associations have the same signs as the direct associations. *V_HBW* had a significantly positive association with *HEIGHT* and *BMI*, and a significantly negative association with *HEALTH*.^28^

**Table 8:** The estimates of the recursive- and structural-forms: U.S.

	Recursive			Structural			
	*HEIGHT*	*EDUCATION*	*MARRIAGE*	*BMI*	*INCOME*	*HEALTH*	*HAPPINESS*
*LBW*	−0.0114	−0.0785	0.0018	−0.838	0.197	−0.0416	0.315
	(0.0117)	(0.197)	(0.0820)	(1.258)	(0.654)	(0.166)	(0.420)
*LBW×OLD*	0.0157	0.109	0.0373	1.041	−0.714	− 0.0277	− 0.431
	(0.0157)	(0.276)	(0.100)	(1.635)	(0.919)	(0.226)	(0.591)
*HBW*	0.0186***	−0.0367	− 0.0941**	1.252*	− 0.0962	0.0218	− 0.0371
	(0.00672)	(0.140)	(0.0419)	(0.759)	(0.451)	(0.0882)	(0.223)
*HBW×OLD*	−0.00088	−0.0315	0.0317	0.318	−1.184*	−0.133	0.205
	(0.0100)	(0.225)	(0.0642)	(1.205)	(0.664)	(0.153)	(0.368)
*V_HBW*	0.0674***	−0.315	0.0767	3.148*	− 0.951	−0.301	− 0.119
	(0.0190)	(0.318)	(0.0798)	(1.739)	(0.621)	(0.193)	(0.514)
*V_HBW×OLD*	−0.0501*	0.721	−0.227*	−1.14	0.793	0.328	0.311
	(0.0303)	(0.441)	(0.125)	(2.486)	(1.103)	(0.286)	(0.675)
*DONTKNOW*	−0.00775	0.187	−0.0383	− 0.375	0.682	− 0.105	− 0.239
	(0.00781)	(0.154)	(0.0474)	(0.849)	(0.523)	(0.106)	(0.252)
*DONTKNOW×OLD*	0.00474	−0.0604	−0.0204	0.751	−1.342**	0.099	0.347
	(0.00935)	(0.198)	(0.0602)	(1.159)	(0.670)	(0.145)	(0.340)
*OLD*	−0.0022	0.0923	−0.116***	− 0.805	0.0535	0.011	0.0177
	(0.00634)	(0.134)	(0.0401)	(0.639)	(0.433)	(0.0913)	(0.232)
*ACADEMIC*	−0.00382**	0.611***	−0.00065	0.155	0.197*	0.043	−0.0251
	(0.00167)	(0.0362)	(0.0112)	(0.200)	(0.112)	(0.0272)	(0.0667)
*HEIGHT*		0.646	0.138	−7.085**	1.928	0.158	0.398
		(0.518)	(0.152)	(3.466)	(1.875)	(0.417)	(1.077)
*EDUCATION*			0.01	−0.206	0.790***	0.0803*	− 0.0323
			(0.00686)	(0.306)	(0.0913)	(0.0411)	(0.106)
*MARRIAGE*				−0.832*	0.737***	0.137**	0.177
				(0.500)	(0.278)	(0.0681)	(0.185)
*BMI*					0.169*	0.00154	−0.0588
					(0.0968)	(0.0217)	(0.0481)
*INCOME*				0.0438		−0.0285	0.118
				(0.355)		(0.0495)	(0.112)
*HEALTH*				−0.918	−1.045*		0.745**
				(1.165)	(0.599)		(0.328)
*HAPPINESS*				−0.524	0.684**	0.185***	
				(0.548)	(0.319)	(0.0686)	
Cons	1.636***	−2.044**	−1.131***	40.37***	−18.12***	1.902	6.612**
	(0.0249)	(0.971)	(0.294)	(8.052)	(5.771)	(1.452)	(2.991)
Obs.	1885	1876	1860	1361	1361	1361	1361
R^2^	0.517	0.303	0.179	0.144	0.045	0.189	0.128
Weak identification test			F(3, 1298)	5.789	9.262	5.307	6.746
*Kleibergen-Paap Wald rk F statistic*							
Underidentification test			χ^2^(3)	17.652***	26.120***	16.429***	20.115***
*Kleibergen-Paap rk LM statistic*							
*F*-value of the first stage			BMI	–	14.32***	14.32***	13.72***
			Income	9.99***	–	10.66***	10.20***
			Health	18.89***	19.41***	–	19.50***
			Happiness	13.39***	13.67***	13.75***	–

The coefficients are estimated by OLS. The definition and unit of each variable are presented in Table 2. Regression coefficients are presented in the upper rows. All control variables are included in the estimation, but their estimates are not shown in the table to save space. The recursive system is estimated with OLS, while structural system is estimated with 2SLS using instrumental variables: the average value of each variable (BMI, income, health, and happiness) with the same three attributes of gender, age, and place of present residence. Robust standard errors are in parentheses. *** *p* < 0.01, ** *p* < 0.05, * *p* < 0.1

#### India

The results for India are presented in Table 9. The magnitude of the absolute values of the coefficients of *LBW* were similar to those in the reduced form, albeit slightly smaller. Consequently, the association of *LBW* with *HEALTH* became insignificant. The associations of *Q_HBW* became insignificant for *BMI* and *HAPPINESS*, and significant only for *HEALTH*.^29^^,^^30^

**Table 9:** The estimates of the recursive- and structural-forms: India

	Recursive			Structural			
	*HEIGHT*	*EDUCATION*	*MARRIAGE*	*BMI*	*INCOME*	*HEALTH*	*HAPPINESS*
*LBW*	−0.0135	−0.377***	0.0869**	−0.626	−2.392**	− 0.162	0.0237
	(0.00988)	(0.126)	(0.043)	(0.653)	(1.210)	(0.172)	(0.254)
*LBW×OLD*	0.00849	0.25	−0.161**	3.103**	−1.604	− 0.424	0.165
	(0.0161)	(0.222)	(0.0787)	(1.237)	(2.369)	(0.291)	(0.418)
*Q_HBW*	0.0133	−0.227	0.0574	3.129	−0.851	0.769***	−0.308
	(0.0222)	(0.324)	(0.0790)	(1.955)	(3.194)	(0.204)	(0.381)
*Q_HBW×OLD*	−0.0265	0.421	0.0489	−0.946	6.119*	−0.576	0.859*
	(0.0356)	(0.480)	(0.0832)	(2.213)	(3.669)	(0.400)	(0.518)
*DONTKNOW*	−0.0119*	0.041	−0.0372	− 0.502	−2.437**	0.077	− 0.393**
	(0.00711)	(0.119)	(0.0379)	(0.560)	(1.200)	(0.113)	(0.192)
*DONTKNOW×OLD*	0.0121	0.568***	−0.00128	1.382*	3.634*	−0.637***	0.36
	(0.0100)	(0.181)	(0.0536)	(0.811)	(1.965)	(0.157)	(0.343)
*OLD*	0.00354	−0.277	−0.132**	0.0772	0.214	0.294*	−0.394
	(0.0110)	(0.179)	(0.0539)	(0.761)	(1.991)	(0.158)	(0.274)
*ACADEMIC*	0.00488**	0.310***	−0.00912	− 0.408**	− 0.0977	− 0.0213	0.137**
	(0.00228)	(0.0392)	(0.0126)	(0.204)	(0.468)	(0.0442)	(0.0685)
*HEIGHT*		0.671	−0.213	−22.79***	−7.172	− 0.491	1.571
		(0.562)	(0.179)	(2.574)	(8.651)	(0.937)	(1.687)
*EDUCATION*			0.000795	0.274	2.102***	0.0206	0.113
			(0.0104)	(0.189)	(0.405)	(0.0454)	(0.0715)
*MARRIAGE*				1.108**	3.129***	0.0817	0.0995
				(0.505)	(1.107)	(0.122)	(0.210)
*BMI*					−0.116	0.0161	0.00237
					(0.300)	(0.0362)	(0.0683)
*INCOME*				−0.0473		0.0173	−0.0511*
				(0.0607)		(0.0140)	(0.0266)
*HEALTH*				−0.0672	2.395*		0.836***
				(0.669)	(1.239)		(0.202)
*HAPPINESS*				0.11	−1.126	0.192***	
				(0.320)	(0.729)	(0.0705)	
Cons	1.584***	2.514**	−0.436	53.64***	−1.99	3.016	0.311
	(0.0364)	(1.043)	(0.362)	(5.915)	(20.22)	(2.103)	(3.935)
Obs.	926	926	926	450	450	450	450
R^2^	0.179	0.245	0.208	0.295	0.325	0.36	0.349
Weak identification test			F(3, 420)	9.217	15.374	9.964	12.613
*Kleibergen-Paap Wald rk F statistic*							
Underidentification test			χ^2^(3)	17.790***	32.265***	21.420***	28.743***
*Kleibergen-Paap rk LM statistic*							
*F*-value of the first stage			BMI	–	16.80***	16.74***	16.22***
			Income	12.17***	–	12.16***	11.90***
			Health	28.79***	29.06***	–	27.15***
			Happiness	41.40***	39.83***	41.49***	–

The coefficients are estimated by OLS. The definition and unit of each variable are presented in Table 2. Regression coefficients are presented in the upper rows. All control variables are included in the estimation, but their estimates are not shown in the table to save space. The recursive system is estimated with OLS, while structural system is estimated with 2SLS using instrumental variables: the average value of each variable (BMI, income, health, and happiness) with the same three attributes of gender, age, and place of present residence. Robust standard errors are in parentheses. *** *p* < 0.01, ** *p* < 0.05, * *p* < 0.1

### Results by full information maximum likelihood (FIML) method

In addition to the recursive-structural form, the whole system of the eight outcomes associated with QOL was estimated with FIML. The estimates did not differ largely from those by recursive-structural estimations, both of which represent the direct association. They confirmed the conclusions deduced in the reduced form estimation. (Estimates of FIML are presented in Additional file 4: Supplemental material D; Tables D-1 to D-3.)

The SEM command of STATA reports the estimates of direct, indirect, and total associations based on the FIML estimation. (The estimates of direct, indirect, and total associations based on FIML are presented in Additional file 5: Supplemental material E; Tables E-1 to E-3.) Therefore, we can compare the estimates of the total associations by FIML with the coefficients estimates in the reduced form. These results were similar, indicating consistency of the estimates by the reduced form and structural form (Additional file 5: Supplemental material E; Tables E-4 to E-6).

## Discussions

The estimation results across the three countries were quite different: while *LBW* was negatively associated with QOL in Japan and India, it did not matter in the U.S. In contrast, *HBW* was a negatively associated with QOL in the U.S. but it was a positively associated with QOL in India.

We now explore whether these results are consistent with previous studies [refer to Additional file 1: Supplemental material A: Literature survey]. As confirmed in our study, in Japan, [4] reported a significantly negative association of low birthweight on academic performance, education, and personal income while using OLS [5]. found that low birthweight was adversely associated with academic performance, which is confirmed in our results. In addition, our finding that the adverse association among younger respondents was mitigated in older respondents is consistent with their finding. However, their conclusion that low birthweight was not associated with education and primary job status contradicts our results.^31^ In sum, using a representative sample of Japan, our study found significant associations with more life outcomes. Furthermore, the analysis of high birthweight has not been done before in Japan.

Since various contradicting results have been reported on the associations of birthweight in the U.S., it is not easy to compare them with ours. For example, though [22] found a positive relationship between birthweight and the variables educational attainment, height, and wage rates, [23] reported only a minor association with education, but no association with health [24]. reported that birthweight showed a significant positive impact on math and reading ability in childhood. Meanwhile, we found that *LBW* was not significant for any outcome, whereas *HBW* had a significant association with several outcomes, including having a negative association with *HEALTH* and *HAPPINESS*.

In addition, the comparison became more difficult because most of the previous studies in the U.S. investigated the linear relationship between birthweight and outcomes concerning QOL, while we examined the associations of birthweight dummies. The linear specification of birthweight precludes the possibility that both low and high birthweights are simultaneously worse than the standard birthweight, which possibly produces contradicting results from ours.

To ease the comparison, we estimated Eq. (1) substituting birthweight (*BWEIGHT*) for birthweight dummies such as *LBW*, *HBW*, and *V_HBW*. (Estimates are presented in Additional file 6: Supplemental material F; Table F-1). The estimates revealed that *BWEIGHT* had a significantly positive relationship with *HEIGHT* and *BMI*, whereas it was significantly and negatively correlated with *HEALTH* and *HAPPINESS*. These results are consistent with previous studies of the U.S. At the same time, these results correspond to the results of the estimates on *V_HBW* for these four outcomes, indicating that the insignificant estimates of *LBW* in the U.S. (Table 5) does not necessarily contradict the results of previous studies, which showed that birthweight is significantly associated with some outcomes concerning QOL.

In addition, [22] reported that the upper quartile (highest 25%) of birthweight negatively affected wage rate, whereas the bottom quartile (lowest 25%) of birthweight had a positive association, which is consistent with our result of negative associations of *V_HBW* with various outcomes.^32^

There have been no papers in India, which to our knowledge, investigated the associations between birthweight and QOL in the field of social science. This study found that *LBW* has a significant and negative association with *ACADEMIC*, *HEIGHT*, *EDUCATION*, and *HEALTH*, while it has significantly positive association with *MARRIAGE*. Meanwhile, *Q_HBW* has a significant and positive association with *BMI*, *INCOME*, and *HEALTH*: that is, higher birthweight has an opposite association compared to that in the U.S.

**Fig. 3:**
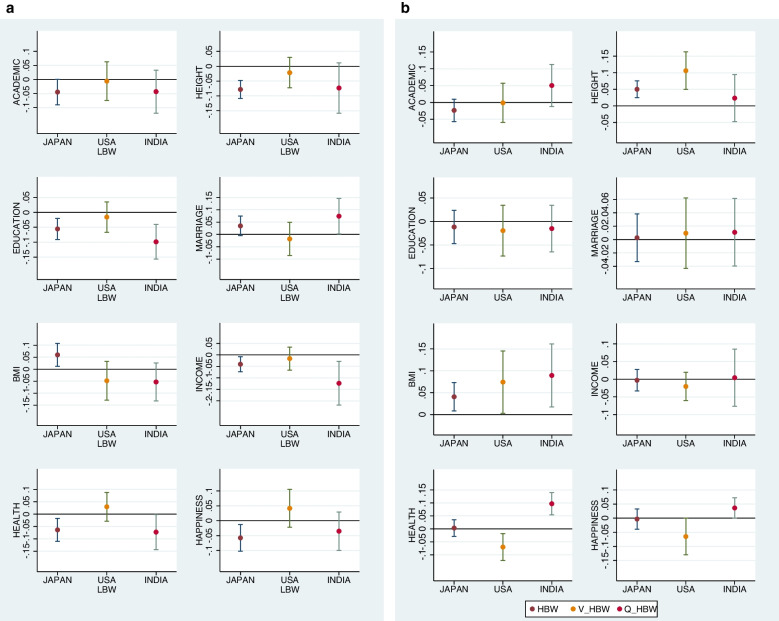
**A** Mean and 95% CI of the coefficient on *LBW* estimated by standardized regression. Notes: The graph is based on the estimates reported in tables C-1 to C-3 in Additional file 3: Supplemental material. In this figure, we present the estimates by standardized regression because it allows the comparison of the magnitudes of estimated coefficients among different regressions. **B** Mean and 95% CI of the coefficients on *HBW*, *V_HBW*, *Q_HBW* for Japan, the U.S., and India, respectively, estimated by standardized regression. Notes: The graph is based on the estimates reported in tables C-1 to C-3 in Additional file 3: Supplemental material. In this figure, we present the estimates by standardized regression because it allows the comparison of the magnitudes of estimated coefficients among different regressions

In sum, the associations of birthweight are widely diversified across the countries: in Japan, while low birthweight was negatively associated with many adult outcomes, the associations of high birthweight were limited. In India, while low birthweight was associated with many outcomes negatively as in Japan, quasi-high birthweight was associated with income, health, and happiness positively. In the U.S., while low birthweight did not have any significant associations, very high birthweight was negatively associated with health and happiness. These are visualized using the estimates by standardized regression (Fig. 3A and B).

Probably, the diverse results across the three countries come from, in part, the differences in BMI in adulthood. It is known that obesity due to affluent society is a problem in the U.S., while malnutrition due to poverty is a serious problem in India.^33^ In our data for the U.S., *BMI* is negatively correlated with *HEIGHT*, *EDUCATION*, *MARRIAGE*, *INCOME*, *HEALTH*, and *HAPPINESS* at the 1% level, whereas in India, it is positively correlated with *MARRIAGE*, *HEALTH*, and *HAPPINESS* at the 1% level (results not shown). Meanwhile, we found that *HBW* had a significantly positive association with *BMI* in the three countries (Tables 4, 5 and 6). These results suggest the possibility that high birthweight led to high BMI in adulthood, which in turn connected negatively to the outcomes in the U.S. and positively to those in India. Furthermore, the result that *HBW* was associated with the outcomes in adulthood, such as *MARRIAGE*, *BMI*, *HEALTH*, *INCOME*, and *HAPPINESS*, but was not associated with *ACADEMIC* and *EDUCATION* in adolescence, is opposite to the results of *LBW*, suggesting that the mechanism of the associations of low and high birthweight might be different.

## Conclusions

This study analyzed the long-term association between birthweight and QOL using the large-scale survey data conducted in Japan, the U.S., and India in 2011. This study is unique in the following ways: First, it investigated the eight outcomes concerning QOL, adolescent academic performance, height, education, marriage, BMI, income, health, and happiness; second, it analyzed the data for three countries, Japan, the U.S., and India, which are widely diversified with respect to birthweight. Third, it investigated whether the associations tend to be mitigated when older; Fourth, it investigated not only the association of low birthweight, but also the association of high birthweight; Fifth, it investigated using not only the estimation of reduced form, but also the recursive-structural forms, accounting for the transmission of the associations between outcomes.

The estimates of the reduced form, which represent the total association of birthweight dummies with outcomes, revealed that low birthweight had significant and negative associations with most of the outcomes in Japan, while high birthweight did not have an association with any outcomes. In contrast, in the U.S., whereas low birthweight did not have an association with any outcomes significantly, very high birthweight was significantly and negatively associated with some outcomes including happiness. In India, low birthweight was associated with most of the outcomes significantly and negatively, whereas quasi-high birthweight was associated with some outcomes including happiness significantly and positively. In addition, these associations were stronger for younger respondents than older ones. Further, the results of the recursive-structural form were consistent with those of the reduced form.

These findings have important implications. First, the finding that the association between low and high birthweight and later QOL are different between countries suggests that the association may partly depend on how society helps citizens facing difficulties from their birthweight. In particular, this study found that, whereas high birthweight has a negative association with *HEALTH* and *HAPPINESS* for younger people in the U.S., no such association was found for older people (Table 5), and that in India, whereas there was a positive association between high birthweight and *INCOME* for older people, no such association was found for younger people. These complex findings might have a root in diversified social conditions among countries.

In this study, we not only investigated the associations assuming them to be independent of each other, but also assuming that they constitute a system in which they heavily depend on each other. Such an approach can elucidate how the association between low- and high-birthweight and one outcome transfers to another outcome. Because the transmission can be prevented by appropriate treatment, such a finding might elucidate weaknesses in society to mitigate the association between low- and high-birthweight and later QOL. We hope that such a study will be made in the future.

This study has limitations. First, the birthweight data were self-reported. Therefore, the data may suffer from memory bias. How might this affect the findings in this study? Since older people probably recalled their birthweights less accurately, the use of recalled birthweights could partly explain the weaker associations between birthweight and outcome variables in the older age group. To be immune from this problem, however, we should collect longitudinal cohort data, which has not been done in Japan.^34^

Second, the lack of information regarding gestational period such as gestational age at birth is another serious limitation of this study, though this study controlled for confounders such as parents’ education and their ages at birth, living standards in childhood, mother’s working status in childhood, and the presence of brothers and sisters. To confirm the causality from birthweight to life outcomes, the information on gestational period is important. Analysis using twin fixed-effects model is an alternative method. Nonetheless, as twin fixed-effects model estimates the effect of the difference in birthweight that occurred in gestation period, the mechanism of how the difference emerges may not be identical between twins and singletons [refer to Additional file 1: Supplementary material A]. Therefore, whether results using twins have external validity to singletons is questionable, which calls for research using singletons, as in this study.

Third, to focus on the associations between low and high birthweight and QOL, we set the entire standard birthweight as the baseline. However, people’s fate may depend on their birthweight even when they are born within the standard birthweight, which constitutes an interesting future study.

## Supplementary Information

**Additional file 1: Supplemental material A.** Survey of the literature.

**Additional file 2: Supplemental material B.** The validity of the outcome variables.

**Additional file 3: Supplemental material C.** The estimates of the standardized regression.

**Additional file 4: Supplemental material D.** Estimates using full-information maximum likelihood (FIML) method.

**Additional file 5: Supplemental material E.** Direct, indirect, and total associations.

**Additional file 6: Supplemental material F.** The estimation results using birthweight instead of birthweight dummies: U.S.

## Data Availability

The datasets used and/or analyzed in this study are available on the website of the Institute of Social and Economic Research, Osaka (https://www.iser.osaka-u.ac.jp/survey_data/top_jp.html, viewed on December 18, 2020), and is obtained with written permission. The conclusions of this article and the associated files are included within the article as supplementary information files.

## Abbreviations

JHPS-CPS: Japan Household Panel Survey on Consumer Preferences and Satisfaction
BMI: Body Mass Index
JPY: Japanese Yen
USD: US Dollar
OLS: Ordinary Least Squares
2SLS: Two-Stage Least Squares
FIML: Full-Information Maximum Likelihood
SEM: Structural Equation Modeling
OECD: Organization for Economic Co-operation and Development
COE: Centers of Excellence

## Footnotes

1 In addition, estimation by recursive and structural forms report the dependency between outcomes.

2 For example, according to our survey data, the rate of low birthweight was 12% in India, while it was 6% in Japan and the U.S.

3 According to [6], the percentage of low-birthweight infants in India was one of the highest globally at 30% similar to Yemen and Sudan. It states that “India alone accounts for 40% of low birthweight births in the developing world and more than half of those in Asia. There are more than 1 million infants born with low birthweight in China and nearly 8 million in India.” Using a representative sample of 60 low- and middle-income countries, [7] investigated the ratio of people whose BMI is lower than 16, and reported that India was the highest at 6.2% in the world; Bangladesh was the second-highest. Though the ratio of Bangladesh has been declining by 0.52% per annum, the decline in India has been only around 0.11% per annum.

4 Schellong et al., [12] conducted a meta-analysis of 66 studies across 26 countries, which investigated the relationship between birthweight and subsequent overweight, and found that there exists a linear relationship between them—high (low) birthweight results in higher (lower) risk of overweight.

5 The questionnaires and the aggregated results are available on the website of the Institute of Social and Economic Research, Osaka University. (https://www.iser.osaka-u.ac.jp/survey_data/top_jp.html, viewed on December 18, 2020).

6 With regard to social class, the respondents were selected evenly across five strata as defined by the education level (illiterate to post-graduate) and occupation (unskilled labor to intermediate and senior civil officials/executives) of householders.

7 A lower number of observations were used in the analysis, primarily because many people did not answer the questions about their income and parent’s education.

8 It is possible and sometimes effective to use the continuous variable of birthweight instead of the low- and high-birthweight dummies. However, because this study focuses on the association between low- and high-birthweight and QOL, and six options were provided for birthweight, we used birthweight dummies rather than a continuous birth weight variable.

9 In the U.S., pounds (lb) are used instead of kg, and the six options are as follows: “less than 5.5 lb.,” “5.5 to 6.9 lb.,” “7.0 lb. to 8.4 lb.,” “8.5 lb. to 9.9 lb.,” “more than 10 lb.,” and “don’t know.” Hereafter, for simplicity, 2.5 kg represents 5.5 lb.; 4 kg, 8.5 lb.; and 4.5 kg, 10 lb. for the U.S. data.

10 According to the most recent available data from OECD, the percentage of low birthweight is 28.0% in India, 9.4% in Japan, and 8.3% in the U.S. (https://stats.oecd.org/Index.aspx?QueryId=81145). The main reason why the percentage of low birthweight in our survey is lower than these values is that many respondents answered “don’t know.” Assuming that respondents who answered “don’t know” has the same distribution over the birthweight of the respondents who remembered birthweight, the percentages of low birthweight become 8.3% in Japan, 7.9% in the U.S., and 20.6% in India, which is closer to the official data.

11 According to [13], “ideal birth weight” in terms of being less likely to develop diabetes in adulthood is lower by 1 kg in India compared with the U.S. Therefore, it would be worthwhile to examine the association of “quasi-high birth weight.”

12 Validity of the outcome variables, especially marriage and BMI, is discussed in Additional file 2: Supplemental material B.

13 In India, the options include “illiterate” and “literate, but not in school.”

14 To check the robustness, we also estimated the income equation deleting the respondents with zero income. The results were qualitatively same.

15 In India, however, the data of the parents’ age at birth were not available.

16 Since there is no data on the region of residence at the age of 15 years for India, we used the city of current residence.

17 Note that this study does not use twin data.

18 In the estimation by ordered logit, odds ratio, which is the exponential of the coefficient, can unambiguously test if the coefficient differs from zero with statistical significance.

19 Since appropriate instrument variables are often difficult to find, we regard the results of the recursive- and structural-form with instrumental variable method for a robustness check of the results of the reduced form.

20 As per academic performance, the survey question asked scores in school at the age of 15 years. Height is determined by around the age of 20 years, and final education is, for most people, between 15 and 22 years. Many people marry after completing school, and according to the results of the questionnaire surveys, divorce and remarriage are relatively rare (especially in Japan).

21 Angrist and Pischke [19], (p197) remarked, “In fact, we can think of OLS as a scheme for computing marginal effects—a scheme that has the virtue of simplicity, automation, and comparability across studies.” After explaining how to estimate the marginal effects of ordered probit, [20] noted “The upshot is that we must be very careful in interpreting the coefficients in this model. Indeed, without a fair amount of extra calculation, it is quite unclear how the coefficients in the ordered probit model should be interpreted.”

22 We investigate the estimates of control variables. Old dummy was positive for *EDUCATION*, whereas significantly negative for *MARRIAGE*, *HEALTH*, and *HAPPINESS*. *MALE* was significantly positive for *HEIGHT*, *EDUCATION*, *MARRIAGE*, *BMI*, and *INCOME*, whereas significantly negative for *HAPPINESS*. It was not significant for *ACADEMIC* and *HEALTH*. Whereas age did not have an association with *ACADEMIC* and *HAPPINESS*, the association of age was U-shaped for *HEALTH*, and inverted U-shaped for the other outcomes. The peaks were 42, 58, and 53 years old for *EDUCATION*, *MARRIAGE*, and *INCOME*, and the trough was 63 years old for *HEALTH*, respectively. As for the important confounders, *F_EDUCATION* was positively associated with *ACADEMIC*, *HEIGHT*, *EDUCATION*, *BMI*, *INCOME*, and *HEALTH*, whereas *M_EDUCATION* had a positive association with *ACADEMIC* and *EDUCATION*. *S_LIVING* also had an association with *ACADEMIC*, *HEIGHT*, *EDUCATION*, *INCOME*, *HEALTH*, and *HAPPINESS*. In addition, “Mothers’ employment status dummy”, especially *M_HOUSEWIFE* was positively associated with *ACADEMIC*, *HEIGHT*, and *EDUCATION*, and was negatively associated with *BMI*. Compared with them, the associations of *F_AGE_BIRTH*, *M_AGE_BIRTH*, and *ONLYCHILD* were limited.

23 We briefly mention the association with controls. *MALE* was positive for *HEIGHT* and *INCOME*, negative for *ACADEMIC*, and insignificant for *EDUCATION*. Age demonstrates an inverted U-shape with respect to *EDUCATION*, *MARRIAGE*, *BMI*, and *INCOME*, while it is U-shaped for *HEALTH* and *HAPPINESS*. *F_EDUCATION* was related to many outcomes as in Japan. In the U.S., *M_EDUCATION* also had similar associations with a couple of outcomes. *S_LIVING* had a positive association with *ACADEMIC*, *HEIGHT*, *EDUCATION*, *HEALTH*, and *HAPPINESS*, and a negative association with *BMI* as in Japan. *M_HOUSEWIFE* was positively related to *INCOME*. However, the effects of *F_AGE_BIRTH*, *M_AGE_BIRTH*, and *ONLYCHILD* were limited as in Japan, except for a positive association of mother’s age at birth with *EDUCATION* and *HEALTH*.

24 As for the associations of the controls, male dummy was significantly positive for *ACADEMIC*, *HEIGHT*, *EDUCATION*, *INCOME*, whereas it was insignificant for *MARRIAGE*, *HEALTH*, and *HAPPINESS*. *MARRIAGE*, *BMI*, and *INCOME* showed an inverted U-shaped association with age. Whereas *F_EDUCATION* was significantly and positively associated with *ACADEMIC*, *EDUCATION*, *INCOME*, and *HEALTH*, *M_EDUCATION* was only positively and significantly associated with *HAPPINESS*. *M_HOUSEWIFE* was associated with *HEALTH* and *HAPPINESS* positively. *S_LIIVNG* was positively and significantly associated with all the outcomes except for *BMI*, *INCOME*, and *HAPPINESS*. *ONLYCHILD* has a positive association only on happiness.

25 Because we adopted the same number of instruments as the number of endogenous variables, the system is just identified. Regarding weak identification, we showed the results of F-test of the excluded instruments and Kleibergen-Paap rk Wald statistic in Tables 5-1 to 5-3. These statistics were large for all the estimations, thereby rejecting the null of the weak identification.

26 As the equation for *ACADEMIC* is the same as the reduced form, it is not reported here.

27 As per the dependences between outcomes, *ACADEMIC* was significantly and positively associated with all outcomes except *BMI*. *EDUCATION* and *HEIGHT* were associated with *INCOME* significantly, while *MARRIAGE* was associated with *HAPPINESS* and *HEALTH* significantly. Regarding the outcomes estimated in the structural form, both *HEALTH* and *INCOME* each showed a mutual positive association with *HAPPINESS*. Further, *HEALTH* was associated with *INCOME* negatively. These results, except for the last, are consistent with our intuitions.

28 As for the associations between outcomes in the recursive system, *ACADEMIC* was associated with *HEIGHT*, *EDUCATION*, and *INCOME*; *EDUCATION* was associated with *INCOME* and *HEALTH*; and *MARRIAGE* was associated with *BMI*, *INCOME*, and *HEALTH*. Regarding the outcomes of the structural system, *HAPPINESS* had positive and mutual associations with *HEALTH* and *INCOME*, as was in Japan. In addition, *INCOME* and *HEALTH* were associated with negatively and mutually.

29 Note that the number of observations of *BMI*, *HEALTH*, and *HAPPINESS* equations in India became half of that of the reduced form estimations because *INCOME* should be included as the regressor for the structural estimation. The decline in significance of *BMI* and *HAPPINESS* may be due to smaller observations.

30 Regarding associations between outcomes of the recursive system, *ACADEMIC* was associated with *HEIGHT*, *EDUCATION*, and *HAPPINESS* significantly and positively. *HEIGHT* was associated with BMI significantly and negatively; *EDUCATION* was associated with *INCOME* significantly and positively; *MARRIAGE* was associated with BMI and *INCOME* significantly and positively. Regarding the four outcomes of the structural form, *HEALTH* affected *INCOME* and *HAPPINESS* significantly and positively. In addition, *HAPPINESS* and *INCOME* were mutually associated significantly and negatively.

31 Though [5] identified the association between birthweight and various life outcomes, their data, based on the Japanese Study of Age and Retirement (JSTAR), is biased as it is limited to a sample of older adults aged 54 and above, and is geographically limited to 10 cities. Furthermore, the sample size was about 270 for their estimation. In contrast, the data of Japan in this study is a representative sample of 3800 people with respect to sex, age, and region. The differences in their results might come from the differences in the data used.

32 In addition, among the medical studies in the U.S., [15] compared those who were born with a birthweight between 2.0–2.5 kg and over 5.0 kg, reporting that the association with the appearance of orthopedically impaired was larger for those over 5.0 kg.

33 According to [25], Southern Asia, including India, has very high stunting prevalence and the highest wasting prevalence.

34 Similar to our study, previous studies in Japan also used self-reported birthweight data.
