# Supplementary material for: Does birthweight matter to quality of life? A comparison between Japan, the U.S., and India
Source: Health Econ Rev. 2022 Sep 20;12:48. doi: 10.1186/s13561-022-00393-9 (PMC9487066; doi:10.1186/s13561-022-00393-9)
Supplement: Supplementary file 1 — Additional file 1: Supplemental material A. Survey of the literature. [file 13561_2022_393_MOESM1_ESM.docx]

# Supplemental material A: Survey of the literature

In this supplement, we survey previous studies on the associations of low birthweight, particularly focusing on three countries— Japan, the U.S., and India. In addition, we also review the literature on high birthweight.

Literature on Japan

Using data by prefecture from the National Survey on School Performance and Learning (Ministry of Education, Culture, Sports, Science and Technology; MEXT) in Japan, t

Kawaguchi and Noguchi (2014) examined the causes and effects of low birthweight using the Ministry of Health, Labor and Welfare’s Neonatal Panel Data, which tracked 47,000 babies born in 2001 nationwide.^^[[1]](#footnote-1)^^ They found that low-birthweight infants had developmental delays at 2.5 years old, but not at 6.5 years old. Their results suggest that low birthweight might not have long-term associations in Japan. However, this cohort study only followed children up to the age of 10, and as the authors admit, “it is premature to conclude from the results of this study that the socioeconomic costs of low birthweight are small”.

Nakamuro et al. (2013) conducted an original web-based survey in Japan through Rakuten Research and collected data on 4,720 twins aged 20–60 years old of their various attributes including birthweight, educational background, income, and academic achievement at age of 15 years. Using OLS, they found that birthweight had a significantly positive effect on education and income. These effects, however, were not significant with the twin fixed-effects model; however, academic achievement at 15 years old became significant. Their results suggest that (1) the fetal origins hypothesis may hold only for the childhood outcomes, and not for income in adulthood, and (2) OLS estimation may overestimate the effect of birthweight, suggesting the importance of controlling for confounders.

Matushima et al. (2018), using data from the Japanese Study of Age and Retirement (JSTAR), reported that low birthweight has adverse effects on academic achievement and health at a young age, whereas it does not affect final education and primary job status. Furthermore, their results show that low birthweight does not affect health in old age, except for an increase in the risk of hypertension and diabetes. Therefore, they concluded that the negative effects of low birthweight diminished in old age.

Literature on the U.S. and other developed countries

In the United States, Behrman and Rosenzweig (2004) used twin data from the Minnesota Twins Registry (MTR) to examine the effects of low birthweight. Monozygotic twins have identical genes, socio-demographics, and dates of birth, and differ only in their mother’s internal environment at birth. Therefore, it is a strict test of Baker’s fetal origins hypothesis. Since the confounding factors are the same, it is possible to ascertain the effect of birthweight on postnatal outcomes as a causal relationship, excluding differences in the environment between the twins after birth. Traditionally, economics has aimed to make societies more efficient through policy interventions, and more recently it focuses, among other things, on identifying causal relationships. While the use of twin data has the advantages of better identifying causal relationships, there remains the problem of “external validity of results”, i.e., whether results that can be validated for twins are also valid for singletons.

Behrman and Rosenzweig (2004) found that birthweight has a significant effect on educational attainment and post-growth height, as well as on wage rates, according to an estimation based on a twin fixed-effects model. The magnitude of the effects was larger than those of ordinary least squares (OLS) estimation, thereby implying that the results of previous analyses may underestimate the effects of birthweight. They also found that birthweight has no effect on body mass in adulthood. Furthermore, the results revealed that the effect of birthweight is linear for education and height, whereas a non-linear effect was found for wage rates—it was negative (positive) for the top (bottom) quartile of birthweight. Though this study has a merit in that it analyzed only monozygotic twins, the number of observations was relatively small at 804.

Royer (2009) used twin data from California State to show that birthweight influences years of education. Specifically, she analyzed data of women who were born as twins in California between 1960 and 1982, who after growing up, gave birth between 1989 and 2002, thereby collating birth data from both time periods to produce a database on 3,396 pairs of twin women. This study estimated a twin fixed-effects model and reported a minor effect on highest educational attainment (increase by 1 or 2 months in years of education for every 1 kg increase in birthweight), while it reported no effect on health (e.g., hypertension, diabetes, coronary artery disease) in adulthood.^^[[2]](#footnote-2)^^

In contrast to Behrman and Rosenzweig (2004), Royer (2009) showed that the twin fixed-effects model estimates were smaller (by 15–30%) than OLS estimates for years of education, suggesting that OLS may overestimate the effect of birthweight by ignoring the effect of unobservable confounders. In addition, Royer (2009) examined the possibility of the non-linearity of the effect of birthweight, and reported that low birthweight (less than 2.5 kg) has a greater effect on mortality within one year and on highest educational attainment than higher birthweight. Further, the author pointed out that many conflicting results had been reported in previous studies, and sometimes even within a single paper, and argued that the results of twin analyses were often unstable.

Using the data of 4,600 individuals from the Panel Study of Income Dynamics (PSID), Chatterji et al. (2014) examined the effect of birthweight on math and reading ability in childhood and found a significantly positive effect. However, its magnitude was such that a 10% weight gain caused only a 0.036 standard deviation (SD) increase in test scores. The effect was mostly observed for low birthweight, and even for low birthweight, an increase of 1 kg led to only a 0.33 SD increase in scores.

Using a representative sample of singleton and twin data from Norway, Black et al. (2007) showed that birthweight positively influences not only short-term outcomes such as neonatal mortality, but also height, IQ, household income, and education (percentage of high school graduates). The advantage of this study is its large sample size of 33,000 twins, which were obtained through collating birth records with subsequent data recorded in the Norwegian Registry, thus allowing them to retain between 5,000 and 10,000 observations per outcome of life (e.g., adult physical and IQ data were from military records, and earned income data were obtained from the tax registry). These, in turn, minimized errors in variables for both the outcome variables and birthweight. The results of their analyses can be summarized as follows: (1) birthweight impacts singleton and twins in the same direction; (2) using a twin sample, the absolute values of the estimates by twin fixed-effects can be larger (BMI, IQ, and education) or smaller (height and income) than those by OLS; and (3) considering the graph of the mean of outcomes for the stratified birthweight—less than 1.5 kg, 1.5–2.5 kg, etc.—there is no difference between the classes for IQ and earned income, whereas, infant mortality rate and IQ exhibited nonlinear association with birthweight: e.g., the infant mortality rate was low for birthweight below 2 kg, and IQ was low for birthweight below 1.5 kg.

As per Result (1) above, birthweight has a significantly positive effect both for singletons and twins on height, IQ, percentage of high school graduates, percentage of full-time workers, and earned income. However, the effect on BMI was different between singletons and twins—birthweight positively affected twins, whereas negatively singletons.^[[3]](#footnote-3)^ Result (1) can be interpreted as supporting the external validity of the results of the twin analysis, and at the same time, the validity of the investigation of singletons—both types of studies are not substitutes, but complements.

In summary, studies using twin data have the advantage of being able to fully control for confounding factors. They suggest that low birthweight may have long-term effects not only on health, but also on the social outcomes of life. However, there are many problems: e.g., the studies have been limited to developed countries, and the results are inconsistent across the literature.

Literature on India

To the best of our knowledge, there are no studies on India that have analyzed the effect of birthweight on long-term socioeconomic outcomes. However, there have been studies related to birthweight focusing on the results of young mothers’ childbirth, some of which used the data of the Consortium for Health Oriented Research in Transitioning Societies (COHORTS), which tracked children from birth to adulthood in India and four other countries (Brazil, Guatemala, Philippines, and South Africa). For example, Fall et al. (2015) found that compared with the births where mothers’ age is between 20 and 24 years old, births by younger mothers as well as late childbearing (mothers’ age is over 35 years old) resulted in lower birthweight, preterm births, undernutrition, and lower completion rates of secondary school. The results suggest that low birthweight may positively correlate with lower school education, and that this correlation is caused by young mothers’ childbirth (as well as late childbearing).^^[[4]](#footnote-4)^^

Literature on high birthweight

Avchen et al. (2001) combined the birth certificates and transcripts of 267,213 Florida-born individuals to investigate the effects of birthweight, stratified by 500 g increments, on school achievement for various disabilities. According to their analysis, birthweight had a significant effect on children with physical and mental disabilities. For children with physical disabilities, the risk was 27% for those whose birthweights were 1.5 kg or less, and then, rapidly declined to 11% for those between 1.5 and 2.0 kg, 3.1% between 2.0 kg and 2.5 kg, and continued to decline until 4.0 kg. However, from 4.0 kg, the risk increases with weight, and for those over 5.0 kg, the risk was 4.5%, which is greater than that of the 2.0–2.5 kg birthweight category. Nevertheless, birthweight had little effect on the risk of developing language and emotional disorders. In summary, the results of Avchen (2001) suggest that not only low birthweight, but high birthweight may as well lead to unfavorable outcomes.^[[5]](#footnote-5)^

References

Aurino, E., W. Schott, M. E. Penny, and J. R. Behrman, 2018. Birth Weight and Prepubertal Body Size Predict Menarcheal Age in India, Peru, and Vietnam. Ann. N.Y. Acad. Sci. 1416, 107–116. doi: 10.1111/nyas.13445

Avchen, R. N., K. G. Scott, and C. A. Mason, 2001. Birth Weight and School-age Disabilities: A Population-based Study. American Journal of Epidemiology 154 (10), 895-901.

Behrman, J. R., and M. R. Rosenzweig, 2004. Returns to Birthweight. Review of Economics and Statistics 86(2), 586-601.

Black, S.E., P. J. Devereux, and K. G. Salvanes, 2007. From the Cradle to the Labor Market? The Effect of Birth Weight on Adult Outcomes. Quarterly Journal of Economics 122(1), 409-439.

Chatterji, P., D. Kim, and K. Lahir, 2014. Birth Weight and Academic Achievement in Childhood. Health Econ 23, 1013-1035. DOI: 10.1002/hec.3074

Fall, C. H. D., H. S. Sachdev, C. Osmond, M. C. Restrepo-Mendez, C. Victora, R. Martorell, A. D Stein, S. Sinha, N. Tandon, L. Adair, I. Bas, S. Norris, L. M Richter, and the COHORTS investigators, 2015. Association between Maternal Age at Childbirth and Child and Adult Outcomes in The off Spring: A Prospective Study in Five Low-Income and Middle-Income Countries (COHORTS collaboration). Lancet Glob Health 3, e366–77.

Fergusson, D. M. and L. J. Woodward, 1999. Maternal Age and Educational and Psychosocial Outcomes in Early Adulthood. J. Child Psychol. Psychiat 43(3), 479-489.

Flensborg-Madsen T. and E. L. Mortensen, 2017. Birth Weight and Intelligence in Young Adulthood and Midlife. Pediatrics 139(6), e2016316.

Kawaguchi, D. and Noguchi, H., 2014. Teitaiju Shussei: Genin to Kiketsu (in Japanese; The Low Birth Weight: The Cause and The Consequence). Global COE Hi-Stat Discussion Paper Series 265.

Kohara, M. and Ohtake, F., 2009. Kodomo No Kyouiku Seika No Kettei Youin (in Japanese; The Determinants of Educational Outcomes of A Child). The Japanese Journal of Labour Studies 588, 67-84.

Kohara, M., M. Matsushima, and F. Ohtake, 2019. Effect of Unemployment on Infant Health. Journal of The Japanese and International Economies 52, 68–77.

DOI 10.1016/j.jjie.2019.03.002

Krishna, A., G. Fink, L. F. Berkman, S.V. Subramanian, 2016. Short-and Long-Run Associations between Birth Weight and Children’s Height. Economics and Human Biology 21. DOI: 10.1016/j.ehb.2016.02.004

Kuzawa, C. W., P. C. Hallal, L. Adair, S. K. Bhargava, C. H.D. Fall, N. Lee, S. A. Norris, C. Osmond, M. Ramirez-Zea, H. S. Sachdev, A. D. Stein, and C. G. Victora, for the COHORTS group, 2012. Birth Weight, Postnatal Weight Gain and Adult Body Composition in Five Low and Middle Income Countries. Am J Hum Biol. 24(1): 5–13. DOI:10.1002/ajhb.21227.

Matsushima, M., S. Shimizutani, H. Yamada, 2018. Life Course Consequences of Low Birth Weight: Evidence from Japan. Journal of the Japanese and International Economics 50, 37-47. DOI:10.1016/j.jjie.2018.07.001

Nakamuro, M., Y. Uzuki, and T. Inui, 2013. The Effects of Birth Weight: Does Fetal Origin Really Matter for Long-Run Outcomes? Economic Letters 121, 53-58.

Raj, A., N. Saggurti, M. Winter, A. Labonte, M. R. Decker, D. Balaiah, and J. G. Silverman, 2010. The Effect of Maternal Child Marriage on Morbidity and Mortality of Children under 5 in India: Cross Sectional Study of A Nationally Representative Sample. BMJ 340.

DOI:10.1136/bmj.b4258

Royer, H., 2009. Separated at Girth: US Twin Estimates of the Effects of Birth Weight. American Economic Journal: Applied Economics 1(1), 49-85.

Schellong, K, S. Schulz, T. Harde, A. Plagemann, 2012. Birth Weight and Long-Term Overweight Risk: Systematic Review and a Meta-Analysis Including 643,902 Persons from 66 Studies and 26 Countries Globally. PLoS ONE 7(10): e47776. DOI:10.1371/journal.pone.0047776

1. As for causes of low birthweight, Kawaguchi and Noguchi (2014) showed that maternal smoking and maternal employment status (whether or not the mother was working full-time six months before birth) had a significant impact. Kohara et al. (2019) examined the effects of unemployment and contingent employment on infant health, using the prefectural panel data from 1975 to 2010. They found that an increase in the unemployment rate of parenting age males led to an increase in the share of low-birthweight babies and a decrease in the average birthweight until 2000; however, this trend declined significantly thereafter. In contrast, since 1985, significant adverse effects of parental contingent employment have persisted over time. [↑](#footnote-ref-1)
2. Royer (2009) stated that there is no effect on income. However, “income” here is the average income in the regions where the subjects live. [↑](#footnote-ref-2)
3. The difference in effects on BMI might be because birthweight of twins is lower than that of singletons (2.598kg vs. 3.528kg). The effect of birthweight on being underweight (BMI of less than 25) or overweight (BMI of greater than 25) is the same for singletons and twins (negative for the former and positive for the latter). [↑](#footnote-ref-3)
4. Studies using the COHORTS data, such as those by Fergusson and Woodward (1999), Raj et al. (2010), and Kuzawa et al. (2012), focused on the effects of maternal age at childbirth on children, especially the possibility that giving birth at a young age (teenage years) may have undesirable consequences. Meanwhile, Aurino et al. (2018) used another cohort data (Young Lives younger cohort for 2001 Indian, Peruvian, and Vietnamese girls born in 2001–2002) to find that babies’ larger birthweight and mothers’ greater height and age at giving birth brought about a later menarche age in the child. Additionally, using data of 3,999 children in Ethiopia, India, Peru, and Vietnam, Krishna et al. (2016) investigated whether the relationship between low birthweight and height depends on the individual’s age and household wealth. They reported that at 6–18 months of age, low-birthweight infants were 0.53 SD shorter than standard-weight infants, though the difference was narrowed to 0.21 SD at 4–5 years old and 0.24 SD at 7–8 years old. However, at this point, the height of low-birthweight infants was lower than average. This result applies for well-off families as well. [↑](#footnote-ref-4)
5. Schellong et al. (2012) and Flensborg-Madsen and Mortensen (2017) pointed out that the *HBW* causes various health problems in adulthood. [↑](#footnote-ref-5)
