# Supplementary material for: Does birthweight matter to quality of life? A comparison between Japan, the U.S., and India
Source: Health Econ Rev. 2022 Sep 20;12:48. doi: 10.1186/s13561-022-00393-9 (PMC9487066; doi:10.1186/s13561-022-00393-9)
Supplement: Supplementary file 2 — Additional file 2: Supplemental material B. The validity of the outcome variables. [file 13561_2022_393_MOESM2_ESM.docx]

# Supplemental material B: The validity of the outcome variables

In this supplement, we consider the validity of the outcome variables, especially *MARRIAGE* and *BMI*, used in the analyses. Our study used eight variables, i.e., academic performance, height, education, marital status, BMI, income, health, and happiness. Though we believe that six variables, i.e., academic performance, height, education, income, health, and happiness, are eligible for the measure of quality of life,^[[1]](#footnote-1)^ we should be careful about how we position *MARRIAGE* and *BMI* for evaluating quality of life.

The role of marriage has changed historically and varies across societies. The same is true for BMI. The percentage of married respondents in this study is 80% in Japan and India, whereas only 60% in the US. Additionally, it has been reported that women have a lower status in India, suggesting that the role of marriage is different among countries. Therefore, whether marriage is a good outcome may depend on society. One solution to how to evaluate marriage in quality of life would be to measure the contribution of marriage to happiness, which is the ultimate goal of life. Many studies in developed countries have shown that marriage has a positive impact on happiness. However, little is known about the impact of marriage on happiness in India^^[[2]](#footnote-2)^^.

Therefore, let us show the results of the analysis of the relationship between happiness and marriage using the data of this study. Test of the difference between the means of happiness of married and unmarried people reveals that married people are significantly happier in the three countries: (mean of married people, mean of unmarried people, test of difference = p-value) was (6.59, 5.80, 0.00) in Japan, (7.50, 6.92, 0.00) in the United States, and (7.50, 6.96, 0.00) in India. Even if we focus only on those of low birthweight, married people were happier. These results suggest that marriage can be evaluated as a positive event in the quality of life evaluation for the three countries of our study.

It is also important to consider how BMI should be evaluated as an outcome measure^^[[3]](#footnote-3)^^. The average BMI of respondents was 22.6 (22.6) in Japan, 28.2 (28.8) in the US, and 23.1 (21.9) in India (country averages according to the WHO are in parentheses). Different societies may have different views on thinness and obesity. In developed countries, there is greater attention on the fact that obesity causes health problems, while in developing countries, being underweight is a health problem. According to the data of this study, the correlation coefficient between happiness and BMI were significantly negative in Japan and the U.S., and significantly positive in India.^[[4]](#footnote-4)^ This suggests that having a high BMI may be evaluated as a positive outcome for quality of life in India, while in the U.S., it may lead to a negative evaluation^^[[5]](#footnote-5)^^.

References

Belot, M., Fidrmuc, J., 2010. Anthropometry of Love: Height and Gender Asymmetries in Interethnic Marriages. Economics and Human Biology 8(3), 361-372.

Case, A., Paxson, C., 2008. Stature and Status: Height, Ability and Labor Market Outcomes. Journal of Political Economy 116(3), 499-532.

Case, A., Paxon, C., 2009. Early Life Health and Cognitive Function in Old Age. American Economic Review 99, 2, 104-109.

Diener, Ed, C. L. Gohm, E. Suh and S. Oishi, 2000. Similarity of the Relations between Marital Status and Subjective Well-Being across Cultures. Journal of Cross-Cultural Psychology 31, 419-436. DOI: 10.1177/0022022100031004001

Dinda S., Gangopadhyay P. K., Chattopadhyay, B.P., Saiyed H. N., Pal, M, Bharati, P., 2006. Height, Weight and Earnings among Coalminers in India. Economics & Human Biology 4, 342–350.

Fall, C. H. D., H. S. Sachdev, C. Osmond, M. C. Restrepo-Mendez, C. Victora, R. Martorell, A. D Stein, S. Sinha, N. Tandon, L. Adair, I. Bas, S. Norris, L. M Richter, and the COHORTS investigators, 2015. Association between Maternal Age at Childbirth and Child and Adult Outcomes in the off Spring: A Prospective Study in Five Low-Income and Middle-Income Countries (COHORTS collaboration). Lancet Glob Health 3, e366–77.

Frey, Bruno S., 2008. Happiness: A Revolution in Economics. MIT Press.

Fu, R. and H. Noguchi, 2016. Does Marriage Make Us Healthier? Inter-Country Comparative Evidence from China, Japan, and Korea. PLoS ONE 11(2), e0148990.

<https://doi.org/10.1371/journal.pone.0148990>

Gao, W., Smyth, R., 2010. Health Human Capital, Height and Wages in China. Journal of Development Studies 46(3), 466-484.

Ghosh, S., S. Lahiri, and N. Datta, 2017. Understanding Happiness and Psychological Wellbeing among Young Married Women in Rural India. Journal of Comparative Family Studies 48 (1),　113-131.

Graham, C., 2011. The Pursuit of Happiness. Brookings Institution.

Heineck, G., 2005. Up in the Skies? The Relationship between Height and Earnings in Germany. Labour 19(3), 469-489.

Lundborg, P., Nystedt, P., Rooth, D. O., 2014. The Height Premium in Earnings: The Role of Physical Capacity and Cognitive and Non-Cognitive Skills. Journal of Human Resources 149(1), 141-161.

Madathil, J. and J.M. Benshoff, 2008. Importance of Marital Characteristics and Marital Satisfaction: A Comparison of Asian Indians in Arranged Marriages and Americans in Marriages of Choice. The Family Journal: Counseling and Therapy for couples and families 16 (3), 222-230. DOI: 10.1177/1066480708317504

Sandhya, S., 2009. The Social Context of Marital Happiness in Urban Indian Couples: Interplay of Intimacy and Conflict. Journal of Marital and Family Therapy 35 (1), 74–96.

Schultz, T. P., 2002. Wage Gains Associated with Height as A Form of Health Human Capital. American Economic Review 92(2), 349-453.

Sohn, K. 2015a. The Height Premium in Indonesia. Economics and Human Biology 16, 1-15.

Sohn, K. 2015b. The Value of Male Height in the Marriage Market. Economics and Human Biology 18, 110-124.

Steckel, R., 1995. Stature and the standard of Living. Journal of Economic Literature 33, 4, 1903-1940.

Tao, LH., 2014. Height, Weight, and Entry Earnings of Female Graduates in Taiwan. Economics and Human Biology 13, C, 85-98.

1. People might doubt whether higher height is really a merit for life. However, it is widely acknowledged that height contributes to success in the labor market (e.g., Schultz 2002; Heineck 2005; Dinda et al. 2006; Case and Paxson 2008, 2009; Gao and Smyth 2010; Lundborg et al. 2014; Tao 2014; Sohn 2015a; Steckel 1995) and in the marriage market (e.g., Belot and Fidrmuc 2010; Sohn 2015b). [↑](#footnote-ref-1)
2. For global results, refer to Frey (2008); For U.S., refer to Diener et al. (2000); and for Japan, Fu and Noguchi (2016). However, there are not many studies of developing countries; Graham (2011) found no relationship between marriage and happiness in Russia, and reported that married people are more unhappy in Afghanistan. In India, various issues related to marriage such as child marriage and young childbearing have been identified (Ghosh et al., 2017; Fall et al., 2015). Also refer to Madathil and Benshoff (2008) and Sandhya (2009). [↑](#footnote-ref-2)
3. Extremely low and high BMIs are considered a health problem. WHO defines a BMI of between 18.5 and 25 as healthy; less than 18.5, underweight (thinness); 25 to 30, overweight; and 30 or more (25 or more in Japan), obese. In addition, “less than two standard deviations” is sometimes used as an evaluation instead of magnitude of BMI. Therefore, in addition to the analysis in the text, we also defined the dummy variables representing low BMI and high BMI and analyzed how low birthweight affects them. However, significant results were not obtained. [↑](#footnote-ref-3)
4. (correlation coefficient, p-value): (-0.045, 0.004) in Japan, (-0.116, 0.000) in the US, and (0.110, 0.000) in India. [↑](#footnote-ref-4)
5. Japan’s BMI is approximately the same as India’s, suggesting that obesity is not a serious problem in Japan as it is in the U.S. [↑](#footnote-ref-5)
