# Supplementary material for: Does birthweight matter to quality of life? A comparison between Japan, the U.S., and India
Source: Health Econ Rev. 2022 Sep 20;12:48. doi: 10.1186/s13561-022-00393-9 (PMC9487066; doi:10.1186/s13561-022-00393-9)
Supplement: Supplementary file 3 — Additional file 3: Supplemental material C. The estimates of the standardized regression. [file 13561_2022_393_MOESM3_ESM.docx]

# Supplemental material C: The estimates of the standardized regression

In this supplemental material, we present the estimation results of the standardized regressions of the reduced form to evaluate the associations of regressors based on the magnitudes of the coefficients instead of the significance. All variables were standardized by subtracting their sample mean and then dividing with their standard deviation. Due to the standardization, all variables had zero mean and unit standard deviation. Therefore, the standardized regression coefficient expresses by how many standard deviations the dependent variable increases, when an explanatory variable increases by one standard deviation.

Subsequently, we examined the magnitude of the estimated coefficients on *LBW* and *HBW* when they were significant compared with those of the other control variables. Specifically, we present the results of the comparison with parental attributes, such as *F_EDUCATION* and *M_AGE_BIRTH*, and with *S_LIVING*. We found that *LBW* and *HBW* have larger or comparable magnitudes with these three variables. However, their impacts were smaller than *AGE* and *MALE*, which had the largest influence of all the control variables for most of the outcome ones.

Japan

For many of the outcome variables, *AGE* and *MALE* exhibited the largest associations. For example, their standardized coefficients were 0.36 and 0.19 for *EDUCATION*, and 1.45 and 0.46 for *INCOME*. Meanwhile, the standardized coefficients on *LBW* were -0.06 for *EDUCATION* and -0.04 for *INCOME*, which were tantamount to one-thirty sixth and one-third of the magnitudes of the associations of *AGE* and *MALE*. However, a more interesting comparison is with *F_EDUCATION* and with *S_LIVING*: the coefficients on *F_EDUCATION* and *S_LIVING* were 0.25 and 0.12 for *EDUCATION* and 0.07 and 0.04 for *INCOME*, respectively. Therefore, though *LBW* had smaller associations with *EDUCATION* than *F_EDUCATION* and *S_LIVING*, it had a comparable association with *INCOME*. *LBW* also had a similar magnitude of association with *HEALTH* as *F_EDUCATION* and *S_LIVING.*

The U.S.

For the U.S., let us focus on the associations of *V_HBW* with *HEALTH* and *HAPPINESS*. The estimates of the standardized coefficients on *V_HBW* were -0.07 and -0.06 for *HEALTH* and *HAPPINESS*, respectively, while they were 0.09 and 0.04 on *F_EDUCATION* and 0.07 and 0.06 on *S_LIVING*, indicating the impact of *V_HBW* was comparable with the association of parents’ education and environment in childhood. However, the impact was smaller than *AGE*, which had the largest association with many outcome variables. For example, the coefficient of *AGE* for *HAPPINESS* was -0.32, which was five times larger than the impact of *V_HBW*.

India

Let us focus on *EDUCATION* and *HEALTH* for the evaluation of the impact of *LBW*. The estimates of the impact were -0.07 (*EDUCATION*) and -0.12 (*HEALTH*). Meanwhile, estimates on *F_EDUCATION* was 0.19 for *EDUCATION* and 0.15 for *HEALTH*, while estimates on *S_LIVING* was 0.27 for *EDUCATION*, and 0.047 for *HEALTH*. In sum, the association of *LBW* was comparable with the association of variables representing respondents’ parents’ education and environment in childhood.

The estimates on *Q_HBW* for *HEALTH* and *HAPPINESS* were 0.10 and 0.04, while those on *F_EDUCATION* were 0.15 and 0.04 and those on *S_LIVING* were 0.05 and 0.03, indicating that they were comparable magnitudes. These results indicate that both low and high birthweight have important implications for India.

Table C-1　The estimates of standardized regression: Japan

|  | *ACADEMIC* | *HEIGHT* | *EDUCATION* | *MARRIAGE* | *BMI* | *INCOME* | *HEALTH* | *HAPPINESS* |
| --- | --- | --- | --- | --- | --- | --- | --- | --- |
| *LBW* | -0.0448* | -0.0782*** | -0.0555*** | 0.0346* | 0.0596** | -0.0404** | -0.0637*** | -0.0576** |
|  | (0.0231) | (0.0154) | (0.0179) | (0.0203) | (0.0243) | (0.0166) | (0.0236) | (0.0227) |
| *LBW×OLD* | 0.00448 | -0.00111 | 0.0163 | -0.0495** | -0.0516** | 0.0548** | 0.0194 | 0.0498** |
|  | (0.0232) | (0.0150) | (0.0194) | (0.0218) | (0.0243) | (0.0256) | (0.0236) | (0.0233) |
| *HBW* | -0.0236 | 0.0502*** | -0.0118 | 0.00258 | 0.0406** | -0.00290 | 0.00306 | -0.00299 |
|  | (0.0170) | (0.0130) | (0.0181) | (0.0182) | (0.0165) | (0.0155) | (0.0163) | (0.0182) |
| *HBW×OLD* | 0.00516 | -0.0160 | 0.0131 | 0.0124 | -0.0341** | -0.0129 | -0.0180 | 0.00163 |
|  | (0.0216) | (0.0137) | (0.0191) | (0.0155) | (0.0166) | (0.0119) | (0.0158) | (0.0126) |
| *DONTKNOW* | -0.105*** | -0.0265 | -0.0716** | -0.100** | -0.0238 | -0.0214 | -0.0690* | -0.0971** |
|  | (0.0405) | (0.0231) | (0.0348) | (0.0406) | (0.0318) | (0.0341) | (0.0403) | (0.0383) |
| *DONTKNOW×OLD* | 0.0714* | -0.0132 | 0.0343 | 0.103** | 0.0103 | 0.0200 | 0.0297 | 0.0775* |
|  | (0.0418) | (0.0243) | (0.0364) | (0.0412) | (0.0343) | (0.0394) | (0.0418) | (0.0402) |
| *OLD* | 0.0129 | -0.0304 | 0.0733*** | -0.121*** | 0.0385 | 0.0407 | -0.0583* | -0.0772** |
|  | (0.0295) | (0.0190) | (0.0260) | (0.0274) | (0.0297) | (0.0328) | (0.0304) | (0.0305) |
| *MALE* | 0.0104 | 0.763*** | 0.188*** | 0.0248 | 0.246*** | 0.463*** | -0.0104 | -0.0517*** |
|  | (0.0158) | (0.0101) | (0.0145) | (0.0153) | (0.0160) | (0.0168) | (0.0161) | (0.0163) |
| *AGE* | 0.0840 | 0.419*** | 0.357*** | 2.277*** | 0.533*** | 1.446*** | -0.411*** | -0.0505 |
|  | (0.125) | (0.0815) | (0.110) | (0.118) | (0.123) | (0.121) | (0.129) | (0.124) |
| *AGESQ* | 0.138 | -0.572*** | -0.491*** | -1.936*** | -0.462*** | -1.446*** | 0.373*** | 0.143 |
|  | (0.121) | (0.0773) | (0.107) | (0.113) | (0.119) | (0.131) | (0.125) | (0.120) |
| *F_EDUCATION* | 0.115*** | 0.0236* | 0.247*** | -0.0129 | -0.0531*** | 0.0727*** | 0.0452** | 0.0192 |
|  | (0.0214) | (0.0127) | (0.0183) | (0.0199) | (0.0199) | (0.0232) | (0.0214) | (0.0219) |
| *M_EDUCATION* | 0.0965*** | 0.0193 | 0.146*** | 0.00199 | 0.0144 | 0.0393 | -0.00471 | 0.00449 |
|  | (0.0222) | (0.0136) | (0.0191) | (0.0210) | (0.0216) | (0.0243) | (0.0220) | (0.0216) |
| *F_AGE_BIRTH* | 0.0169 | 0.0102 | 0.0601*** | -0.0426* | 0.00112 | 0.00294 | 0.00388 | -0.0354 |
|  | (0.0254) | (0.0147) | (0.0214) | (0.0241) | (0.0251) | (0.0264) | (0.0247) | (0.0271) |
| *M_AGE_BIRTH* | -0.0297 | -0.0150 | -0.0288 | 0.00710 | -0.0157 | 0.00414 | -0.00490 | 0.0164 |
|  | (0.0249) | (0.0149) | (0.0212) | (0.0234) | (0.0253) | (0.0268) | (0.0247) | (0.0268) |
| *M_FULLTIME* | 0.177*** | 0.0672** | 0.238*** | 0.0187 | -0.0590 | 0.0917 | -0.0358 | 0.0834 |
|  | (0.0544) | (0.0300) | (0.0376) | (0.0519) | (0.0548) | (0.0567) | (0.0504) | (0.0583) |
| *M_PARTTIME* | 0.152*** | 0.0584** | 0.216*** | -0.0128 | -0.0886* | 0.0949* | -0.0436 | 0.0833 |
|  | (0.0493) | (0.0272) | (0.0344) | (0.0473) | (0.0496) | (0.0515) | (0.0457) | (0.0530) |
| *M_HOUSEWIFE* | 0.200*** | 0.0693** | 0.235*** | -0.0266 | -0.117** | 0.105* | -0.0283 | 0.0781 |
|  | (0.0526) | (0.0288) | (0.0364) | (0.0500) | (0.0525) | (0.0558) | (0.0484) | (0.0558) |
| *S_LIVING* | 0.0808*** | 0.0215** | 0.122*** | 0.0134 | -0.000848 | 0.0433** | 0.0686*** | 0.160*** |
|  | (0.0181) | (0.0109) | (0.0151) | (0.0166) | (0.0178) | (0.0203) | (0.0183) | (0.0190) |
| *ONLYCHILD* | -0.00297 | -0.0135 | 0.0211 | -0.0290* | 0.0273 | 0.000561 | 0.000221 | 0.0103 |
|  | (0.0156) | (0.0104) | (0.0148) | (0.0168) | (0.0173) | (0.0188) | (0.0168) | (0.0158) |
| *RELIGION* | -0.0429*** | -0.0144 | -0.0273* | 0.000702 | 0.0137 | -0.0355** | -0.0505*** | 0.0223 |
|  | (0.0162) | (0.0103) | (0.0144) | (0.0147) | (0.0156) | (0.0167) | (0.0174) | (0.0176) |
| Cons | 0.0105 | -0.00584 | 0.0112 | 0.00616 | -0.00690 | -0.0809*** | 0.0117 | 0.00475 |
|  | (0.0156) | (0.00975) | (0.0140) | (0.0148) | (0.0155) | (0.0175) | (0.0159) | (0.0160) |
| Obs. | 3,814 | 3,839 | 3,845 | 3,860 | 3,776 | 2,742 | 3,837 | 3,791 |
| R^2^ | 0.091 | 0.642 | 0.269 | 0.164 | 0.104 | 0.270 | 0.056 | 0.053 |

Note: The region dummies representing the prefecture where respondents lived at age 15 are included in the estimation, but not shown here to save space. Robust standard errors are in parentheses. *** p<0.01, ** p<0.05, * p<0.1

Table C-2　The estimates of standardized regression: USA

|  | *ACADEMIC* | *HEIGHT* | *EDUCATION* | *MARRIAGE* | *BMI* | *INCOME* | *HEALTH* | *HAPPINESS* |
| --- | --- | --- | --- | --- | --- | --- | --- | --- |
| *LBW* | -0.00604 | -0.0217 | -0.0159 | -0.0181 | -0.0482 | -0.0160 | 0.0298 | 0.0416 |
|  | (0.0350) | (0.0261) | (0.0261) | (0.0342) | (0.0413) | (0.0255) | (0.0298) | (0.0323) |
| *LBW×OLD* | -0.0268 | 0.0273 | 0.00876 | 0.0222 | 0.0534 | -0.0249 | -0.0539 | -0.0320 |
|  | (0.0348) | (0.0285) | (0.0298) | (0.0346) | (0.0416) | (0.0290) | (0.0334) | (0.0351) |
| *HBW* | 0.0130 | 0.0625*** | 0.00753 | -0.0732*** | 0.0631* | -0.0130 | -0.0222 | -0.0228 |
|  | (0.0297) | (0.0220) | (0.0266) | (0.0274) | (0.0354) | (0.0281) | (0.0281) | (0.0304) |
| *HBW×OLD* | -0.0341 | 0.000558 | -0.0189 | 0.0308 | -0.0108 | -0.0432 | 0.00666 | -0.0157 |
|  | (0.0315) | (0.0241) | (0.0302) | (0.0308) | (0.0363) | (0.0307) | (0.0307) | (0.0329) |
| *V_HBW* | -0.00124 | 0.106*** | -0.0195 | 0.00943 | 0.0741** | -0.0205 | -0.0701*** | -0.0649** |
|  | (0.0297) | (0.0289) | (0.0275) | (0.0269) | (0.0363) | (0.0203) | (0.0265) | (0.0331) |
| *V_HBW×OLD* | 0.0120 | -0.0609 | 0.0406 | -0.0363 | -0.0329 | 0.0413 | 0.0657* | 0.0656** |
|  | (0.0336) | (0.0371) | (0.0315) | (0.0323) | (0.0424) | (0.0316) | (0.0338) | (0.0324) |
| *DONTKNOW* | 0.0237 | -0.0241 | 0.0314 | -0.00910 | -0.0153 | 0.0645* | -0.0737** | -0.0308 |
|  | (0.0394) | (0.0295) | (0.0337) | (0.0356) | (0.0398) | (0.0388) | (0.0342) | (0.0349) |
| *DONTKNOW×OLD* | -0.0102 | 0.0105 | 0.00309 | -0.0384 | 0.0318 | -0.0863** | 0.0582 | 0.0168 |
|  | (0.0420) | (0.0305) | (0.0368) | (0.0387) | (0.0427) | (0.0428) | (0.0373) | (0.0379) |
| *OLD* | 0.0181 | -0.00655 | 0.0106 | -0.0977** | -0.00741 | -0.00451 | 0.00193 | -0.000501 |
|  | (0.0419) | (0.0303) | (0.0363) | (0.0379) | (0.0432) | (0.0408) | (0.0392) | (0.0425) |
| *MALE* | -0.0935*** | 0.675*** | 0.0264 | 0.0329 | 0.0259 | 0.223*** | 0.0248 | 0.0394* |
|  | (0.0220) | (0.0166) | (0.0203) | (0.0207) | (0.0227) | (0.0215) | (0.0210) | (0.0216) |
| *AGE* | -0.0390 | 0.0786 | 0.502*** | 1.986*** | 0.839*** | 1.552*** | -0.425*** | -0.323** |
|  | (0.143) | (0.107) | (0.123) | (0.124) | (0.152) | (0.114) | (0.132) | (0.134) |
| *AGESQ* | 0.0901 | -0.116 | -0.397*** | -1.814*** | -0.893*** | -1.485*** | 0.239* | 0.406*** |
|  | (0.133) | (0.0974) | (0.120) | (0.122) | (0.133) | (0.112) | (0.123) | (0.123) |
| *F_EDUCATION* | 0.0886*** | 0.0183 | 0.244*** | 0.0232 | -0.106*** | 0.151*** | 0.0879*** | 0.0362 |
|  | (0.0288) | (0.0220) | (0.0259) | (0.0266) | (0.0299) | (0.0315) | (0.0261) | (0.0298) |
| *M_EDUCATION* | 0.132*** | 0.0583*** | 0.172*** | -0.0667** | -0.0432 | 0.0144 | 0.0497* | -0.00811 |
|  | (0.0285) | (0.0214) | (0.0264) | (0.0263) | (0.0284) | (0.0282) | (0.0268) | (0.0282) |
| *F_AGE_BIRTH* | -0.0248 | -0.0181 | -0.00380 | -0.0631* | 0.0179 | 0.0210 | -0.0318 | 0.00807 |
|  | (0.0351) | (0.0266) | (0.0325) | (0.0345) | (0.0380) | (0.0332) | (0.0326) | (0.0338) |
| *M_AGE_BIRTH* | 0.0400 | 0.0114 | 0.0917*** | 0.0162 | -0.0317 | 0.0117 | 0.0667** | 0.0327 |
|  | (0.0354) | (0.0273) | (0.0322) | (0.0336) | (0.0380) | (0.0316) | (0.0332) | (0.0351) |
| *M_FULLTIME* | 0.0844 | -0.0758 | 0.0602 | 0.0795 | 0.00169 | 0.142*** | 0.0997 | -0.0742 |
|  | (0.0795) | (0.0545) | (0.0660) | (0.0677) | (0.0980) | (0.0510) | (0.0691) | (0.0795) |
| *M_PARTTIME* | 0.120* | -0.0261 | 0.106* | 0.0435 | -0.0432 | 0.154*** | 0.112* | -0.0363 |
|  | (0.0670) | (0.0461) | (0.0561) | (0.0573) | (0.0821) | (0.0451) | (0.0584) | (0.0673) |
| *M_HOUSEWIFE* | 0.110 | -0.0616 | 0.0854 | 0.0709 | -0.0250 | 0.132*** | 0.0969 | -0.0341 |
|  | (0.0727) | (0.0504) | (0.0608) | (0.0622) | (0.0905) | (0.0479) | (0.0636) | (0.0731) |
| *S_LIVING* | 0.0578** | 0.0343** | 0.0497** | 0.00250 | -0.0664** | 0.00990 | 0.0671*** | 0.0596** |
|  | (0.0239) | (0.0175) | (0.0211) | (0.0227) | (0.0259) | (0.0211) | (0.0236) | (0.0253) |
| *ONLYCHILD* | 0.0227 | 0.00325 | 0.0301 | -0.0411* | 0.0358 | -0.0170 | -0.0301 | -0.0321 |
|  | (0.0206) | (0.0156) | (0.0203) | (0.0216) | (0.0242) | (0.0220) | (0.0205) | (0.0230) |
| *RELIGION* | 0.0382* | 0.0263 | 0.0445** | 0.105*** | 0.00767 | -0.0210 | 0.0557*** | 0.115*** |
|  | (0.0231) | (0.0168) | (0.0207) | (0.0211) | (0.0240) | (0.0223) | (0.0215) | (0.0227) |
| Cons | 0.0262 | 0.0551*** | 0.0490** | -0.0124 | -0.00131 | -0.0556*** | 0.0129 | 0.0170 |
|  | (0.0211) | (0.0161) | (0.0199) | (0.0205) | (0.0227) | (0.0209) | (0.0205) | (0.0212) |
| Obs. | 2,109 | 1,938 | 2,156 | 2,144 | 1,892 | 1,676 | 2,166 | 2,090 |
| R^2^ | 0.075 | 0.513 | 0.176 | 0.170 | 0.078 | 0.198 | 0.117 | 0.055 |

Note: The region dummies representing the state where respondents lived at age 15 are included in the estimation, but not shown here to save space. Robust standard errors are in parentheses. *** p<0.01, ** p<0.05, * p<0.1

Table C-3　The estimates of standardized regression: India

|  | *ACADEMIC* | *HEIGHT* | *EDUCATION* | *MARRIAGE* | *BMI* | *INCOME* | *HEALTH* | *HAPPINESS* |
| --- | --- | --- | --- | --- | --- | --- | --- | --- |
| *LBW* | -0.0433 | -0.0735* | -0.0988*** | 0.0741** | -0.0533 | -0.123** | -0.0727** | -0.0351 |
|  | (0.0390) | (0.0431) | (0.0296) | (0.0366) | (0.0404) | (0.0484) | (0.0361) | (0.0329) |
| *LBW×OLD* | 0.0300 | 0.0217 | 0.0496 | -0.0687* | 0.0902** | 0.00421 | -0.0259 | -0.00911 |
|  | (0.0448) | (0.0440) | (0.0365) | (0.0399) | (0.0439) | (0.0638) | (0.0405) | (0.0348) |
| *Q_HBW* | 0.0503 | 0.0235 | -0.0152 | 0.0108 | 0.0893** | 0.00426 | 0.0968*** | 0.0362** |
|  | (0.0318) | (0.0360) | (0.0252) | (0.0257) | (0.0367) | (0.0412) | (0.0218) | (0.0182) |
| *Q_HBW×OLD* | -0.0199 | -0.0243 | 0.0262 | 0.0101 | -0.00318 | 0.0548** | -0.00607 | 0.00216 |
|  | (0.0300) | (0.0321) | (0.0212) | (0.0150) | (0.0268) | (0.0261) | (0.0225) | (0.0112) |
| *DONTKNOW* | -0.112** | -0.0781* | -0.0720* | -0.0110 | 0.0200 | -0.117* | -0.0842** | -0.143*** |
|  | (0.0435) | (0.0438) | (0.0411) | (0.0458) | (0.0482) | (0.0620) | (0.0412) | (0.0379) |
| *DONTKNOW×OLD* | 0.0600 | 0.0559 | 0.135*** | -0.0369 | 0.129*** | 0.147** | -0.147*** | -0.0166 |
|  | (0.0477) | (0.0436) | (0.0468) | (0.0472) | (0.0486) | (0.0683) | (0.0438) | (0.0417) |
| *OLD* | -0.0795 | 0.0101 | -0.115* | -0.128** | -0.118* | -0.0970 | 0.0639 | -0.0290 |
|  | (0.0673) | (0.0645) | (0.0610) | (0.0621) | (0.0618) | (0.103) | (0.0606) | (0.0550) |
| *MALE* | 0.0597** | 0.363*** | 0.199*** | 0.00838 | -0.0192 | 0.269*** | 0.0166 | 0.0119 |
|  | (0.0288) | (0.0297) | (0.0278) | (0.0287) | (0.0306) | (0.0530) | (0.0270) | (0.0249) |
| *AGE* | -0.394* | -0.223 | -0.360* | 2.490*** | 1.159*** | 1.217*** | -0.349* | -0.00804 |
|  | (0.209) | (0.210) | (0.199) | (0.237) | (0.226) | (0.293) | (0.206) | (0.197) |
| *AGESQ* | 0.395* | 0.207 | 0.166 | -2.253*** | -1.071*** | -1.161*** | 0.216 | 0.00648 |
|  | (0.228) | (0.216) | (0.221) | (0.247) | (0.237) | (0.337) | (0.224) | (0.219) |
| *F_EDUCATION* | 0.134*** | 0.0318 | 0.191*** | -0.0382 | 0.0539 | 0.346*** | 0.145*** | 0.0445 |
|  | (0.0483) | (0.0542) | (0.0431) | (0.0456) | (0.0645) | (0.0930) | (0.0502) | (0.0464) |
| *M_EDUCATION* | 0.00400 | -0.0131 | 0.0277 | 0.0122 | 0.0254 | -0.00793 | 0.0128 | 0.0715 |
|  | (0.0499) | (0.0548) | (0.0443) | (0.0444) | (0.0651) | (0.0722) | (0.0490) | (0.0446) |
| *M_FULLTIME* | -0.0594 | 0.0260 | 0.0868 | 0.0381 | 0.0406 | -0.0656 | 0.0594 | 0.149 |
|  | (0.0921) | (0.0499) | (0.0694) | (0.0759) | (0.0526) | (0.102) | (0.0804) | (0.0935) |
| *M_PARTTIME* | -0.0297 | -0.0112 | 0.0575 | 0.0309 | 0.00602 | -0.0626 | 0.0896 | 0.0628 |
|  | (0.0713) | (0.0409) | (0.0504) | (0.0589) | (0.0409) | (0.0734) | (0.0600) | (0.0708) |
| *M_HOUSEWIFE* | -0.113 | 0.0458 | 0.127 | 0.0725 | -0.0445 | -0.0992 | 0.210** | 0.209* |
|  | (0.107) | (0.0536) | (0.0790) | (0.0901) | (0.0571) | (0.122) | (0.0960) | (0.113) |
| *S_LIVING* | 0.0717** | 0.0674** | 0.271*** | 0.0637** | -0.0210 | 0.0595 | 0.0465* | 0.0298 |
|  | (0.0311) | (0.0294) | (0.0274) | (0.0292) | (0.0310) | (0.0373) | (0.0272) | (0.0248) |
| *ONLYCHILD* | -0.0120 | 0.0219 | 0.0377 | -0.0448 | -0.0330 | 0.00922 | 0.00910 | 0.0459** |
|  | (0.0295) | (0.0344) | (0.0285) | (0.0298) | (0.0319) | (0.0375) | (0.0296) | (0.0218) |
| *RELIGION* | -0.107*** | -0.0627* | -0.0225 | 0.0199 | -0.0425 | -0.0773 | 0.132*** | 0.143*** |
|  | (0.0321) | (0.0349) | (0.0302) | (0.0308) | (0.0318) | (0.0557) | (0.0305) | (0.0263) |
| Cons | -0.106** | 0.00376 | -0.0741 | -0.0753 | -0.0661 | -0.251*** | 0.0348 | -0.0168 |
|  | (0.0480) | (0.0473) | (0.0451) | (0.0472) | (0.0448) | (0.0844) | (0.0434) | (0.0375) |
| Obs. | 926 | 1,037 | 1,037 | 1,037 | 1,036 | 484 | 1,037 | 1,037 |
| R^2^ | 0.175 | 0.182 | 0.247 | 0.185 | 0.093 | 0.271 | 0.294 | 0.392 |

Note: The region dummies representing the city where respondents currently live are included in the estimation, but not shown here to save space. Robust standard errors are in parentheses. *** p<0.01, ** p<0.05, * p<0.1
