# Supplementary material for: Does birthweight matter to quality of life? A comparison between Japan, the U.S., and India
Source: Health Econ Rev. 2022 Sep 20;12:48. doi: 10.1186/s13561-022-00393-9 (PMC9487066; doi:10.1186/s13561-022-00393-9)
Supplement: Supplementary file 4 — Additional file 4: Supplemental material D. Estimates using full-information maximum likelihood (FIML) method. [file 13561_2022_393_MOESM4_ESM.docx]

# Supplemental material D: Estimates using full-information maximum likelihood (FIML) method

In this section, we present the estimates by full-information maximum likelihood (FIML) method for the robustness check of the results of recursive and structural estimations in the text. Specifically, we conducted FIML estimation of the whole system consists of eight outcomes, using the same instrumental variables, and the same structure as the recursive- and structural-forms in the text using SEM command of STATA. The estimation method is different from the text in that it allowed the correlation among disturbance terms of eight outcomes, and that it used the maximum likelihood method instead of least squares. Most of the estimates were found to be qualitatively same, revealing the robustness of the conclusions of the text.

Japan

The estimates by FIML represent direct associations like 2SLS estimation. The estimates of *LBW* and *HBW* were close to those by recursive and structural estimations except that the associations of *LBW* became insignificant for *ACADEMIC* and *INCOME*, whereas it became significant for *HAPPINESS*. The estimates on *HBW* were close to those by 2SLS in the text. The coefficients of the interaction terms of birthweight dummies and old dummy were qualitatively the same as those in the text, supporting Hypothesis 2.

The U.S.

The estimates of birthweight dummies by FIML were similar to those by 2SLS except that *V_HBW* became significantly negative to *HEALTH*. Further, the conclusion (Hypothesis 2) in the text that the significant associations with younger respondents were mitigated for older respondents was confirmed by the FIML estimation.

India

The estimates by FIML were generally consistent with those by 2SLS for four outcome variables, *BMI*, *INCOME*, *HEALTH* and *HAPPINESS.* Specifically, *LBW* was significant for *INCOME* and *HEALTH*, and *Q_HBW* was significant for *BMI* and *HEALTH*. However, as per the other outcome variables—*ACADEMIC*, *HEIGHT*, *EDUCATION*, and *MARRIAGE—LBW* became insignificant for *EDUCATION* and *MARRIAGE*, when estimated by FIML. This might be as the number of observations for FIML were about half (from 926 to 450) of that for the OLS estimations for *ACADEMIC*, *HEIGHT*, *EDUCATION*, and *MARRIAGE*.^[[1]](#footnote-1)^

Table D-1 The estimates of FIML: Japan

|  | *ACADEMIC* | *HEIGHT* | *EDUCATION* | *MARRIAGE* | *BMI* | *INCOME* | *HEALTH* | *HAPPINESS* |
| --- | --- | --- | --- | --- | --- | --- | --- | --- |
| *LBW* | -0.121 | -0.0287*** | -0.369** | 0.0843* | 0.792** | -0.280 | -0.115 | -0.326* |
|  | (0.119) | (0.00611) | (0.185) | (0.0434) | (0.343) | (0.305) | (0.105) | (0.192) |
| *LBW×OLD* | -0.0853 | 0.00541 | 0.0520 | -0.175*** | -1.117** | 1.000** | 0.0339 | 0.665** |
|  | (0.171) | (0.00879) | (0.266) | (0.0622) | (0.492) | (0.437) | (0.151) | (0.275) |
| *HBW* | -0.280 | 0.0386*** | -0.316 | 0.0703 | 1.283** | 0.234 | 0.0353 | 0.207 |
|  | (0.188) | (0.00969) | (0.294) | (0.0687) | (0.542) | (0.483) | (0.166) | (0.304) |
| *HBW×OLD* | -0.128 | -0.0270 | 0.248 | 0.114 | -2.053 | -0.996 | -0.238 | -0.156 |
|  | (0.465) | (0.0239) | (0.722) | (0.169) | (1.334) | (1.186) | (0.409) | (0.749) |
| *DONTKNOW* | -0.253** | -0.00885* | -0.138 | -0.0962** | -0.652** | 0.159 | -0.149 | -0.300* |
|  | (0.104) | (0.00535) | (0.162) | (0.0379) | (0.300) | (0.266) | (0.0917) | (0.168) |
| *DONTKNOW×OLD* | 0.187 | 0.00113 | 0.106 | 0.105** | 0.420 | -0.0985 | 0.0696 | 0.285 |
|  | (0.122) | (0.00629) | (0.190) | (0.0445) | (0.352) | (0.313) | (0.108) | (0.197) |
| *OLD* | -0.0166 | -0.00755** | 0.304*** | -0.0836*** | -0.128 | 0.123 | 0.0344 | -0.0302 |
|  | (0.0749) | (0.00385) | (0.117) | (0.0273) | (0.216) | (0.192) | (0.0692) | (0.122) |
| *ACADEMIC* |  | 0.00288*** | 0.677*** | 0.0135* | -0.0573 | 0.321*** | 0.0679*** | 0.185*** |
|  |  | (0.00101) | (0.0306) | (0.00781) | (0.0616) | (0.0548) | (0.0189) | (0.0345) |
| *HEIGHT* |  |  | 0.664 | 0.126 | -1.752 | 2.552*** | -0.114 | 0.827 |
|  |  |  | (0.594) | (0.139) | (1.097) | (0.976) | (0.336) | (0.615) |
| *EDUCATION* |  |  |  | -0.00272 | 0.0427 | 0.254*** | 0.00854 | 0.0560*** |
|  |  |  |  | (0.00460) | (0.0363) | (0.0324) | (0.0111) | (0.0203) |
| *MARRIAGE* |  |  |  |  | 0.0133 | 0.366*** | 0.299*** | 0.974*** |
|  |  |  |  |  | (0.155) | (0.138) | (0.0477) | (0.0873) |
| *IV_BMI* |  |  |  |  | 0.855*** |  |  |  |
|  |  |  |  |  | (0.102) |  |  |  |
| *IV_INCOME* |  |  |  |  |  | 0.913*** |  |  |
|  |  |  |  |  |  | (0.0739) |  |  |
| *IV_HEALTH* |  |  |  |  |  |  | 0.892*** |  |
|  |  |  |  |  |  |  | (0.120) |  |
| *IV_HAPPINESS* |  |  |  |  |  |  |  | 0.866*** |
|  |  |  |  |  |  |  |  | (0.102) |
| Cons | 2.093*** | 1.497*** | -2.585** | -1.558*** | 6.726** | -8.756*** | 0.808 | -0.990 |
|  | (0.397) | (0.0205) | (1.085) | (0.254) | (2.755) | (1.823) | (0.823) | (1.339) |
| Obs. | 2,587 | 2,587 | 2,587 | 2,587 | 2,587 | 2,587 | 2,587 | 2,587 |

Note: All control variables are included in the estimation, but not shown here to save space. Robust standard errors are in parentheses. *** p<0.01, ** p<0.05, * p<0.1

Table D-2 The estimates of FIML: USA

|  | *ACADEMIC* | *HEIGHT* | *EDUCATION* | *MARRIAGE* | *BMI* | *INCOME* | *HEALTH* | *HAPPINESS* |
| --- | --- | --- | --- | --- | --- | --- | --- | --- |
| *LBW* | -0.0502 | -0.00471 | -0.222 | 0.0119 | -1.268 | 0.235 | -0.0345 | 0.364 |
|  | (0.194) | (0.0134) | (0.282) | (0.0861) | (1.241) | (0.642) | (0.168) | (0.409) |
| *LBW×OLD* | -0.115 | 0.0166 | 0.259 | -0.0451 | 1.631 | -0.725 | -0.0812 | -0.729 |
|  | (0.257) | (0.0178) | (0.374) | (0.114) | (1.645) | (0.851) | (0.222) | (0.543) |
| *HBW* | 0.00853 | 0.0166** | -0.108 | -0.0962** | 1.210* | 0.0681 | 0.0217 | -0.138 |
|  | (0.106) | (0.00734) | (0.155) | (0.0472) | (0.681) | (0.352) | (0.0920) | (0.225) |
| *HBW×OLD* | -0.208 | -0.00622 | 0.0511 | 0.00133 | 0.235 | -0.945* | -0.121 | 0.0259 |
|  | (0.168) | (0.0117) | (0.245) | (0.0747) | (1.077) | (0.557) | (0.145) | (0.355) |
| *V_HBW* | -0.115 | 0.0576*** | -0.260 | 0.00393 | 3.741*** | -0.0853 | -0.415** | -0.649 |
|  | (0.209) | (0.0145) | (0.306) | (0.0933) | (1.343) | (0.696) | (0.181) | (0.443) |
| *V_HBW×OLD* | 0.319 | -0.0433** | 0.796* | -0.130 | -1.701 | 0.0955 | 0.433* | 0.802 |
|  | (0.299) | (0.0207) | (0.435) | (0.133) | (1.916) | (0.994) | (0.259) | (0.632) |
| *DONTKNOW* | 0.111 | -0.00594 | 0.146 | -0.0148 | -0.208 | 0.667* | -0.120 | -0.306 |
|  | (0.116) | (0.00801) | (0.168) | (0.0514) | (0.741) | (0.383) | (0.100) | (0.244) |
| *DONTKNOW×OLD* | 0.0135 | 0.00944 | -0.0774 | -0.0992 | 0.503 | -1.321*** | 0.128 | 0.360 |
|  | (0.153) | (0.0106) | (0.223) | (0.0681) | (0.982) | (0.508) | (0.133) | (0.324) |
| *OLD* | 0.0263 | -0.00569 | 0.0508 | -0.0523 | -0.616 | -0.0103 | -0.0365 | -0.0425 |
|  | (0.107) | (0.00738) | (0.155) | (0.0473) | (0.683) | (0.353) | (0.0926) | (0.226) |
| *ACADEMIC* |  | -0.00456** | 0.616*** | -0.00983 | 0.0949 | 0.194** | 0.0285 | 0.0169 |
|  |  | (0.00188) | (0.0395) | (0.0131) | (0.189) | (0.0975) | (0.0255) | (0.0622) |
| *HEIGHT* |  |  | 0.247 | 0.198 | -7.598*** | 1.096 | 0.348 | 1.545* |
|  |  |  | (0.570) | (0.174) | (2.506) | (1.296) | (0.338) | (0.828) |
| *EDUCATION* |  |  |  | 0.0206** | -0.338*** | 0.707*** | 0.0850*** | 0.139*** |
|  |  |  |  | (0.00827) | (0.119) | (0.0620) | (0.0161) | (0.0394) |
| *MARRIAGE* |  |  |  |  | -1.215*** | 0.594*** | 0.223*** | 0.452*** |
|  |  |  |  |  | (0.390) | (0.202) | (0.0528) | (0.129) |
| *IV_BMI* |  |  |  |  | 0.851*** |  |  |  |
|  |  |  |  |  | (0.121) |  |  |  |
| *IV_INCOME* |  |  |  |  |  | 0.551*** |  |  |
|  |  |  |  |  |  | (0.0937) |  |  |
| *IV_HEALTH* |  |  |  |  |  |  | 0.863*** |  |
|  |  |  |  |  |  |  | (0.119) |  |
| *IV_HAPPINESS* |  |  |  |  |  |  |  | 0.867*** |
|  |  |  |  |  |  |  |  | (0.132) |
| Cons | 2.654*** | 1.636*** | -0.934 | -1.346*** | 16.53*** | -9.257*** | -0.529 | -1.924 |
|  | (0.397) | (0.0279) | (1.101) | (0.336) | (5.454) | (2.562) | (0.847) | (1.985) |
| Obs. | 1,361 | 1,361 | 1,361 | 1,361 | 1,361 | 1,361 | 1,361 | 1,361 |

Note: All control variables are included in the estimation, but not shown here to save space. Robust standard errors are in parentheses. *** p<0.01, ** p<0.05, * p<0.1

Table D-3 The estimates of FIML: India

|  | *ACADEMIC* | *HEIGHT* | *EDUCATION* | *MARRIAGE* | *BMI* | *INCOME* | *HEALTH* | *HAPPINESS* |
| --- | --- | --- | --- | --- | --- | --- | --- | --- |
| *LBW* | -0.274 | -0.0120 | -0.338 | 0.0793 | -0.441 | -3.017** | -0.305** | -0.0280 |
|  | (0.171) | (0.0145) | (0.224) | (0.0669) | (0.600) | (1.351) | (0.130) | (0.198) |
| *LBW×OLD* | 0.389 | -0.0190 | 0.293 | -0.00877 | 2.695*** | -1.324 | -0.257 | -0.179 |
|  | (0.298) | (0.0253) | (0.389) | (0.116) | (1.042) | (2.353) | (0.225) | (0.344) |
| *Q_HBW* | 0.810** | 0.0209 | -0.171 | 0.0162 | 3.053** | 1.117 | 0.881*** | 0.522 |
|  | (0.373) | (0.0317) | (0.488) | (0.145) | (1.303) | (2.936) | (0.281) | (0.431) |
| *Q_HBW×OLD* | -0.267 | -0.0497 | 0.442 | 0.0993 | -1.114 | 4.790 | -0.375 | -0.241 |
|  | (0.591) | (0.0500) | (0.770) | (0.230) | (2.057) | (4.633) | (0.444) | (0.682) |
| *DONTKNOW* | -0.315** | -0.0139 | -0.0663 | -0.0273 | -0.256 | -1.527 | -0.113 | -0.308** |
|  | (0.132) | (0.0112) | (0.173) | (0.0516) | (0.463) | (1.044) | (0.100) | (0.153) |
| *DONTKNOW×OLD* | 0.234 | 0.0156 | 0.557** | 0.0572 | 0.483 | 2.256 | -0.185 | 0.0921 |
|  | (0.194) | (0.0165) | (0.254) | (0.0760) | (0.689) | (1.533) | (0.155) | (0.228) |
| *OLD* | -0.415** | -0.00662 | -0.550** | -0.219*** | -0.0242 | 0.913 | 0.144 | -0.00952 |
|  | (0.201) | (0.0171) | (0.263) | (0.0787) | (0.711) | (1.601) | (0.154) | (0.236) |
| *ACADEMIC* |  | 0.00557 | 0.320*** | -0.00635 | -0.274 | -0.161 | -0.00625 | 0.0948* |
|  |  | (0.00398) | (0.0615) | (0.0189) | (0.169) | (0.380) | (0.0365) | (0.0561) |
| *HEIGHT* |  |  | 0.456 | 0.0289 | -19.89*** | -8.936** | -0.676 | 0.910 |
|  |  |  | (0.726) | (0.216) | (1.961) | (4.368) | (0.419) | (0.642) |
| *EDUCATION* |  |  |  | -0.00376 | 0.187 | 1.980*** | 0.0624** | 0.0525 |
|  |  |  |  | (0.0140) | (0.126) | (0.285) | (0.0272) | (0.0416) |
| *MARRIAGE* |  |  |  |  | 0.997** | 3.016*** | 0.120 | 0.134 |
|  |  |  |  |  | (0.422) | (0.952) | (0.0914) | (0.140) |
| *IV_BMI* |  |  |  |  | 0.769*** |  |  |  |
|  |  |  |  |  | (0.0990) |  |  |  |
| *IV_INCOME* |  |  |  |  |  | 0.796*** |  |  |
|  |  |  |  |  |  | (0.112) |  |  |
| *IV_HEALTH* |  |  |  |  |  |  | 0.958*** |  |
|  |  |  |  |  |  |  | (0.108) |  |
| *IV_HAPPINESS* |  |  |  |  |  |  |  | 0.974*** |
|  |  |  |  |  |  |  |  | (0.0904) |
| Cons | 5.709*** | 1.606*** | 2.254 | -1.494*** | 37.35*** | 3.824 | 0.645 | -2.841* |
|  | (0.680) | (0.0618) | (1.505) | (0.449) | (4.598) | (9.214) | (0.992) | (1.510) |
| Obs. | 450 | 450 | 450 | 450 | 450 | 450 | 450 | 450 |

Note: All control variables are included in the estimation, but not shown here to save space. Robust standard errors are in parentheses. *** p<0.01, ** p<0.05, * p<0.1

1. This is because *INCOME* is included in all equations of FIML. [↑](#footnote-ref-1)
