# Supplementary material for: Does birthweight matter to quality of life? A comparison between Japan, the U.S., and India
Source: Health Econ Rev. 2022 Sep 20;12:48. doi: 10.1186/s13561-022-00393-9 (PMC9487066; doi:10.1186/s13561-022-00393-9)
Supplement: Supplementary file 5 — Additional file 5: Supplemental material E. Direct, indirect, and total associations. [file 13561_2022_393_MOESM5_ESM.docx]

# Supplemental material E: Direct, indirect, and total associations

In Supplemental material E, we present the FIML estimation using SEM command of STATA, which represent direct associations of the regressors. In addition to this, the command also calculates the indirect and the total associations of all exogenous variables. In this supplemental material, we present the estimates of the direct, indirect, and total associations of the key variables in Tables E-1 to E-3. The estimates of the coefficients and their standard errors are shown under the headings of “Coef.” and “Std. Err.”^[[1]](#footnote-1)^

Since the estimates of the reduced form represents the total associations, while the estimates of the recursive-structural form in the text represents the direct associations, we can check the robustness of these estimates, comparing with the estimates of the direct and total associations estimated by SEM command. Specifically, we examined if the estimates of the reduced form (by OLS) and of the recursive- (by OLS) and structural-forms (by 2SLS) were similar to the estimates by FIML. These comparisons are presented in Tables E-4 to E-6.

In the Tables F-4 to F-6, the estimates of birthweight dummies were copied from Tables E-1 to E-3 (FIML), from Tables 5-1 to 5-3 (recursive-structural form), and from Tables 4-1 to 4-3 (reduced form). Comparisons were made when the estimates of direct association by FIML were significant at the 10%. We calculated the ratio of the two direct associations by dividing estimates of FIML with the estimates of recursive-structural form, and the ratio of the two total associations by dividing estimates of FIML with the estimates of the reduced form. These ratios are colored in red and shown in the right-most two columns in Tables E-4 to E-6. All the ratios were not radically different from 1, suggesting that the estimations of the reduced and recursive-structural forms are robust for the three countries.

Table E-1　Direct, indirect, and total associations: Japan

|  | *HEIGHT* | | | | | |
| --- | --- | --- | --- | --- | --- | --- |
|  | Direct |  | Indirect |  | Total |  |
|  | Coef. | Std. Err. | Coef. | Std. Err. | Coef. | Std. Err. |
| *LBW* | -0.029*** | 0.006 | -0.0003 | 0.000 | -0.029*** | 0.006 |
| *LBW×OLD* | 0.005 | 0.009 | -0.0002 | 0.001 | 0.005 | 0.009 |
| *HBW* | 0.039*** | 0.010 | -0.0008 | 0.001 | 0.038*** | 0.010 |
| *HBW×OLD* | -0.027 | 0.024 | -0.0004 | 0.001 | -0.027 | 0.024 |
| *DONTKNOW* | -0.009* | 0.005 | -0.0007* | 0.000 | -0.010* | 0.005 |
| *DONTKNOW×OLD* | 0.001 | 0.006 | 0.0005 | 0.000 | 0.002 | 0.006 |
| *OLD* | -0.008** | 0.004 | -0.00005 | 0.000 | -0.008** | 0.004 |
| *ACADEMIC* | 0.003*** | 0.001 | - | (no path) | 0.003*** | 0.001 |
|  | *EDUCATION* | | | | | |
|  | Direct |  | Indirect |  | Total |  |
|  | Coef. | Std. Err. | Coef. | Std. Err. | Coef. | Std. Err. |
| *LBW* | -0.369** | 0.185 | -0.101 | 0.083 | -0.471** | 0.201 |
| *LBW×OLD* | 0.052 | 0.266 | -0.054 | 0.116 | -0.002 | 0.290 |
| *HBW* | -0.316 | 0.294 | -0.164 | 0.130 | -0.481 | 0.319 |
| *HBW×OLD* | 0.248 | 0.722 | -0.105 | 0.316 | 0.143 | 0.788 |
| *DONTKNOW* | -0.138 | 0.162 | -0.177** | 0.071 | -0.315* | 0.176 |
| *DONTKNOW×OLD* | 0.106 | 0.190 | 0.127 | 0.083 | 0.233 | 0.207 |
| *OLD* | 0.304*** | 0.117 | -0.016 | 0.051 | 0.288** | 0.127 |
| *ACADEMIC* | 0.677*** | 0.031 | 0.002 | 0.002 | 0.678*** | 0.031 |
| *HEIGHT* | 0.664 | 0.594 | - | (no path) | 0.664 | 0.594 |
|  | *MARRIAGE* | | | | | |
|  | Direct |  | Indirect |  | Total |  |
|  | Coef. | Std. Err. | Coef. | Std. Err. | Coef. | Std. Err. |
| *LBW* | 0.084* | 0.043 | -0.004 | 0.005 | 0.080* | 0.043 |
| *LBW×OLD* | -0.175*** | 0.062 | 0.000 | 0.003 | -0.176*** | 0.062 |
| *HBW* | 0.070 | 0.069 | 0.002 | 0.006 | 0.073 | 0.069 |
| *HBW×OLD* | 0.114 | 0.169 | -0.006 | 0.008 | 0.108 | 0.169 |
| *DONTKNOW* | -0.096** | 0.038 | -0.004 | 0.003 | -0.100*** | 0.038 |
| *DONTKNOW×OLD* | 0.105** | 0.045 | 0.002 | 0.002 | 0.107** | 0.045 |
| *OLD* | -0.084*** | 0.027 | -0.002 | 0.002 | -0.086*** | 0.027 |
| *ACADEMIC* | 0.013* | 0.008 | -0.001 | 0.003 | 0.012* | 0.007 |
| *HEIGHT* | 0.126 | 0.139 | -0.002 | 0.003 | 0.124 | 0.139 |
| *EDUCATION* | -0.003 | 0.005 | - | (no path) | -0.003 | 0.005 |
|  | *BMI* | | | | | |
|  | Direct |  | Indirect |  | Total |  |
|  | Coef. | Std. Err. | Coef. | Std. Err. | Coef. | Std. Err. |
| *LBW* | 0.792** | 0.343 | 0.039 | 0.040 | 0.831** | 0.341 |
| *LBW×OLD* | -1.117** | 0.492 | -0.007 | 0.035 | -1.124** | 0.491 |
| *HBW* | 1.283** | 0.542 | -0.070 | 0.053 | 1.213** | 0.541 |
| *HBW×OLD* | -2.053 | 1.334 | 0.063 | 0.065 | -1.990 | 1.334 |
| *DONTKNOW* | -0.652** | 0.300 | 0.016 | 0.026 | -0.635** | 0.299 |
| *DONTKNOW×OLD* | 0.420 | 0.352 | -0.002 | 0.024 | 0.418 | 0.352 |
| *OLD* | -0.128 | 0.216 | 0.025 | 0.021 | -0.103 | 0.216 |
| *ACADEMIC* | -0.057 | 0.062 | 0.024 | 0.025 | -0.033 | 0.056 |
| *HEIGHT* | -1.752 | 1.097 | 0.030 | 0.040 | -1.722 | 1.097 |
| *EDUCATION* | 0.043 | 0.036 | -0.00004 | 0.0004 | 0.043 | 0.036 |
| *MARRIAGE* | 0.013 | 0.155 | - | (no path) | 0.013 | 0.155 |
| *IV_BMI* | 0.855*** | 0.102 | - | (no path) | 0.855*** | 0.102 |
|  | *INCOME* | | | | | |
|  | Direct |  | Indirect |  | Total |  |
|  | Coef. | Std. Err. | Coef. | Std. Err. | Coef. | Std. Err. |
| *LBW* | -0.280 | 0.305 | -0.203** | 0.086 | -0.483 | 0.313 |
| *LBW×OLD* | 1.000** | 0.437 | -0.079 | 0.117 | 0.921** | 0.451 |
| *HBW* | 0.234 | 0.483 | -0.089 | 0.133 | 0.145 | 0.497 |
| *HBW×OLD* | -0.996 | 1.186 | -0.035 | 0.313 | -1.031 | 1.226 |
| *DONTKNOW* | 0.159 | 0.266 | -0.222*** | 0.073 | -0.064 | 0.274 |
| *DONTKNOW×OLD* | -0.098 | 0.313 | 0.163* | 0.084 | 0.064 | 0.323 |
| *OLD* | 0.123 | 0.192 | 0.017 | 0.053 | 0.140 | 0.198 |
| *ACADEMIC* | 0.321*** | 0.055 | 0.184*** | 0.024 | 0.505*** | 0.051 |
| *HEIGHT* | 2.552*** | 0.976 | 0.214 | 0.161 | 2.766*** | 0.988 |
| *EDUCATION* | 0.254*** | 0.032 | -0.001 | 0.002 | 0.253*** | 0.032 |
| *MARRIAGE* | 0.366*** | 0.138 | - | (no path) | 0.366*** | 0.138 |
| *IV_INCOME* | 0.913*** | 0.074 | - | (no path) | 0.913*** | 0.074 |
|  | *HEALTH* | | | | | |
|  | Direct |  | Indirect |  | Total |  |
|  | Coef. | Std. Err. | Coef. | Std. Err. | Coef. | Std. Err. |
| *LBW* | -0.115 | 0.105 | 0.015 | 0.020 | -0.100 | 0.106 |
| *LBW×OLD* | 0.034 | 0.151 | -0.059** | 0.024 | -0.025 | 0.152 |
| *HBW* | 0.035 | 0.166 | -0.006 | 0.029 | 0.030 | 0.168 |
| *HBW×OLD* | -0.238 | 0.409 | 0.028 | 0.063 | -0.210 | 0.413 |
| *DONTKNOW* | -0.149 | 0.092 | -0.049*** | 0.016 | -0.198** | 0.092 |
| *DONTKNOW×OLD* | 0.070 | 0.108 | 0.047*** | 0.017 | 0.116 | 0.109 |
| *OLD* | 0.034 | 0.069 | -0.023** | 0.012 | 0.011 | 0.070 |
| *ACADEMIC* | 0.068*** | 0.019 | 0.009 | 0.008 | 0.077*** | 0.017 |
| *HEIGHT* | -0.114 | 0.336 | 0.043 | 0.043 | -0.071 | 0.339 |
| *EDUCATION* | 0.009 | 0.011 | -0.001 | 0.001 | 0.008 | 0.011 |
| *MARRIAGE* | 0.299*** | 0.048 | - | (no path) | 0.299*** | 0.048 |
| *IV_HEALTH* | 0.892*** | 0.120 | - | (no path) | 0.892*** | 0.120 |
|  | *HAPPINESS* | | | | | |
|  | Direct |  | Indirect |  | Total |  |
|  | Coef. | Std. Err. | Coef. | Std. Err. | Coef. | Std. Err. |
| *LBW* | -0.326* | 0.192 | 0.005 | 0.056 | -0.320 | 0.198 |
| *LBW×OLD* | 0.665** | 0.275 | -0.183** | 0.076 | 0.482* | 0.285 |
| *HBW* | 0.207 | 0.304 | 0.023 | 0.087 | 0.231 | 0.314 |
| *HBW×OLD* | -0.156 | 0.749 | 0.067 | 0.204 | -0.089 | 0.775 |
| *DONTKNOW* | -0.300* | 0.168 | -0.170*** | 0.047 | -0.469*** | 0.173 |
| *DONTKNOW×OLD* | 0.285 | 0.197 | 0.153*** | 0.055 | 0.439** | 0.204 |
| *OLD* | -0.030 | 0.122 | -0.077** | 0.034 | -0.107 | 0.126 |
| *ACADEMIC* | 0.185*** | 0.035 | 0.052*** | 0.016 | 0.237*** | 0.032 |
| *HEIGHT* | 0.827 | 0.615 | 0.158 | 0.140 | 0.986 | 0.630 |
| *EDUCATION* | 0.056*** | 0.020 | -0.003 | 0.004 | 0.053*** | 0.021 |
| *MARRIAGE* | 0.974*** | 0.087 | - | (no path) | 0.974*** | 0.087 |
| *IV_HAPPINESS* | 0.866*** | 0.102 | - | (no path) | 0.866*** | 0.102 |

Note: *** p<0.01, ** p<0.05, * p<0.1

Table E-2　Direct, indirect, and total associations: USA

|  | *HEIGHT* | | | | | |
| --- | --- | --- | --- | --- | --- | --- |
|  | Direct | | Indirect | | Total | |
|  | Coef. | Std. Err. | Coef. | Std. Err. | Coef. | Std. Err. |
| *LBW* | -0.005 | 0.013 | 0.0002 | 0.001 | -0.004 | 0.013 |
| *LBW×OLD* | 0.017 | 0.018 | 0.0005 | 0.001 | 0.017 | 0.018 |
| *HBW* | 0.017** | 0.007 | -0.00004 | 0.0005 | 0.017** | 0.007 |
| *HBW×OLD* | -0.006 | 0.012 | 0.0009 | 0.001 | -0.005 | 0.012 |
| *V_HBW* | 0.058*** | 0.014 | 0.0005 | 0.001 | 0.058*** | 0.014 |
| *V_HBW×OLD* | -0.043** | 0.021 | -0.0015 | 0.001 | -0.045** | 0.021 |
| *DONTKNOW* | -0.006 | 0.008 | -0.0005 | 0.001 | -0.006 | 0.008 |
| *DONTKNOW×OLD* | 0.009 | 0.011 | -0.0001 | 0.001 | 0.009 | 0.011 |
| *OLD* | -0.006 | 0.007 | -0.0001 | 0.0005 | -0.006 | 0.007 |
| *ACADEMIC* | -0.005** | 0.002 | - | (no path) | -0.005** | 0.002 |
|  | *EDUCATION* | | | | | |
|  | Direct | | Indirect | | Total | |
|  | Coef. | Std. Err. | Coef. | Std. Err. | Coef. | Std. Err. |
| *LBW* | -0.222 | 0.282 | -0.032 | 0.119 | -0.254 | 0.306 |
| *LBW×OLD* | 0.259 | 0.374 | -0.067 | 0.158 | 0.192 | 0.406 |
| *HBW* | -0.108 | 0.155 | 0.009 | 0.066 | -0.098 | 0.168 |
| *HBW×OLD* | 0.051 | 0.245 | -0.129 | 0.104 | -0.078 | 0.266 |
| *V_HBW* | -0.260 | 0.306 | -0.057 | 0.133 | -0.317 | 0.330 |
| *V_HBW×OLD* | 0.796* | 0.435 | 0.186 | 0.186 | 0.981** | 0.471 |
| *DONTKNOW* | 0.146 | 0.168 | 0.067 | 0.071 | 0.212 | 0.183 |
| *DONTKNOW×OLD* | -0.077 | 0.223 | 0.011 | 0.094 | -0.067 | 0.242 |
| *OLD* | 0.051 | 0.155 | 0.015 | 0.066 | 0.066 | 0.168 |
| *ACADEMIC* | 0.616*** | 0.039 | -0.001 | 0.003 | 0.615*** | 0.039 |
| *HEIGHT* | 0.247 | 0.570 | - | (no path) | 0.247 | 0.570 |
|  | *MARRIAGE* | | | | | |
|  | Direct | | Indirect | | Total | |
|  | Coef. | Std. Err. | Coef. | Std. Err. | Coef. | Std. Err. |
| *LBW* | 0.012 | 0.086 | -0.006 | 0.007 | 0.006 | 0.086 |
| *LBW×OLD* | -0.045 | 0.114 | 0.008 | 0.009 | -0.037 | 0.114 |
| *HBW* | -0.096** | 0.047 | 0.001 | 0.005 | -0.095** | 0.047 |
| *HBW×OLD* | 0.001 | 0.075 | -0.001 | 0.006 | 0.001 | 0.075 |
| *V_HBW* | 0.004 | 0.093 | 0.006 | 0.012 | 0.010 | 0.093 |
| *V_HBW×OLD* | -0.130 | 0.133 | 0.008 | 0.015 | -0.121 | 0.133 |
| *DONTKNOW* | -0.015 | 0.051 | 0.002 | 0.004 | -0.013 | 0.052 |
| *DONTKNOW×OLD* | -0.099 | 0.068 | 0.0003 | 0.005 | -0.099 | 0.068 |
| *OLD* | -0.052 | 0.047 | -0.0001 | 0.004 | -0.052 | 0.047 |
| *ACADEMIC* | -0.010 | 0.013 | 0.012** | 0.005 | 0.002 | 0.012 |
| *HEIGHT* | 0.198 | 0.174 | 0.005 | 0.012 | 0.203 | 0.174 |
| *EDUCATION* | 0.021** | 0.008 | - | (no path) | 0.021** | 0.008 |
|  | *BMI* | | | | | |
|  | Direct | | Indirect | | Total | |
|  | Coef. | Std. Err. | Coef. | Std. Err. | Coef. | Std. Err. |
| *LBW* | -1.268 | 1.241 | 0.107 | 0.184 | -1.161 | 1.254 |
| *LBW×OLD* | 1.631 | 1.645 | -0.161 | 0.248 | 1.470 | 1.661 |
| *HBW* | 1.210* | 0.681 | 0.024 | 0.115 | 1.234* | 0.686 |
| *HBW×OLD* | 0.235 | 1.077 | 0.046 | 0.163 | 0.281 | 1.087 |
| *V_HBW* | 3.741*** | 1.343 | -0.358 | 0.246 | 3.383** | 1.349 |
| *V_HBW×OLD* | -1.701 | 1.916 | 0.186 | 0.323 | -1.515 | 1.929 |
| *DONTKNOW* | -0.208 | 0.741 | 0.003 | 0.113 | -0.205 | 0.748 |
| *DONTKNOW×OLD* | 0.503 | 0.982 | 0.073 | 0.151 | 0.576 | 0.991 |
| *OLD* | -0.616 | 0.683 | 0.088 | 0.103 | -0.528 | 0.690 |
| *ACADEMIC* | 0.095 | 0.189 | -0.176** | 0.079 | -0.081 | 0.175 |
| *HEIGHT* | -7.598*** | 2.506 | -0.330 | 0.307 | -7.927*** | 2.522 |
| *EDUCATION* | -0.338*** | 0.119 | -0.025* | 0.013 | -0.363*** | 0.120 |
| *MARRIAGE* | -1.215*** | 0.390 | - | (no path) | -1.215*** | 0.390 |
| *IV_BMI* | 0.851*** | 0.121 | - | (no path) | 0.851*** | 0.121 |
|  | *INCOME* | | | | | |
|  | Direct | | Indirect | | Total | |
|  | Coef. | Std. Err. | Coef. | Std. Err. | Coef. | Std. Err. |
| *LBW* | 0.235 | 0.642 | -0.190 | 0.243 | 0.045 | 0.686 |
| *LBW×OLD* | -0.725 | 0.851 | 0.111 | 0.323 | -0.614 | 0.909 |
| *HBW* | 0.068 | 0.352 | -0.106 | 0.136 | -0.038 | 0.375 |
| *HBW×OLD* | -0.945* | 0.557 | -0.101 | 0.211 | -1.046* | 0.595 |
| *V_HBW* | -0.085 | 0.696 | -0.177 | 0.272 | -0.262 | 0.740 |
| *V_HBW×OLD* | 0.095 | 0.994 | 0.635* | 0.383 | 0.730 | 1.059 |
| *DONTKNOW* | 0.667* | 0.383 | 0.157 | 0.146 | 0.824** | 0.409 |
| *DONTKNOW×OLD* | -1.321*** | 0.508 | -0.093 | 0.193 | -1.414*** | 0.542 |
| *OLD* | -0.010 | 0.353 | 0.014 | 0.134 | 0.004 | 0.377 |
| *ACADEMIC* | 0.194** | 0.098 | 0.431*** | 0.049 | 0.625*** | 0.094 |
| *HEIGHT* | 1.096 | 1.296 | 0.295 | 0.425 | 1.391 | 1.362 |
| *EDUCATION* | 0.707*** | 0.062 | 0.012* | 0.006 | 0.720*** | 0.062 |
| *MARRIAGE* | 0.594*** | 0.202 | - | (no path) | 0.594*** | 0.202 |
| *IV_INCOME* | 0.551*** | 0.094 | - | (no path) | 0.551*** | 0.094 |
|  | *HEALTH* | | | | | |
|  | Direct | | Indirect | | Total | |
|  | Coef. | Std. Err. | Coef. | Std. Err. | Coef. | Std. Err. |
| *LBW* | -0.035 | 0.168 | -0.023 | 0.036 | -0.058 | 0.172 |
| *LBW×OLD* | -0.081 | 0.222 | 0.011 | 0.048 | -0.070 | 0.227 |
| *HBW* | 0.022 | 0.092 | -0.024 | 0.021 | -0.002 | 0.094 |
| *HBW×OLD* | -0.121 | 0.145 | -0.014 | 0.031 | -0.136 | 0.149 |
| *V_HBW* | -0.415** | 0.181 | -0.008 | 0.043 | -0.423** | 0.184 |
| *V_HBW×OLD* | 0.433* | 0.259 | 0.050 | 0.059 | 0.483* | 0.264 |
| *DONTKNOW* | -0.120 | 0.100 | 0.016 | 0.022 | -0.104 | 0.102 |
| *DONTKNOW×OLD* | 0.128 | 0.133 | -0.024 | 0.029 | 0.104 | 0.136 |
| *OLD* | -0.036 | 0.093 | -0.007 | 0.020 | -0.044 | 0.095 |
| *ACADEMIC* | 0.028 | 0.026 | 0.051*** | 0.011 | 0.080*** | 0.024 |
| *HEIGHT* | 0.348 | 0.338 | 0.066 | 0.065 | 0.414 | 0.344 |
| *EDUCATION* | 0.085*** | 0.016 | 0.005** | 0.002 | 0.090*** | 0.016 |
| *MARRIAGE* | 0.223*** | 0.053 | - | (no path) | 0.223*** | 0.053 |
| *IV_HEALTH* | 0.863*** | 0.119 | - | (no path) | 0.863*** | 0.119 |
|  | *HAPPINESS* | | | | | |
|  | Direct | | Indirect | | Total | |
|  | Coef. | Std. Err. | Coef. | Std. Err. | Coef. | Std. Err. |
| *LBW* | 0.364 | 0.409 | -0.040 | 0.065 | 0.324 | 0.414 |
| *LBW×OLD* | -0.729 | 0.543 | 0.035 | 0.087 | -0.694 | 0.549 |
| *HBW* | -0.138 | 0.225 | -0.031 | 0.040 | -0.169 | 0.227 |
| *HBW×OLD* | 0.026 | 0.355 | -0.022 | 0.057 | 0.004 | 0.359 |
| *V_HBW* | -0.649 | 0.443 | 0.048 | 0.085 | -0.601 | 0.446 |
| *V_HBW×OLD* | 0.802 | 0.632 | 0.018 | 0.112 | 0.820 | 0.637 |
| *DONTKNOW* | -0.306 | 0.244 | 0.016 | 0.040 | -0.290 | 0.247 |
| *DONTKNOW×OLD* | 0.360 | 0.324 | -0.039 | 0.053 | 0.321 | 0.327 |
| *OLD* | -0.043 | 0.226 | -0.023 | 0.036 | -0.066 | 0.228 |
| *ACADEMIC* | 0.017 | 0.062 | 0.079*** | 0.026 | 0.096* | 0.058 |
| *HEIGHT* | 1.545* | 0.828 | 0.126 | 0.119 | 1.671** | 0.835 |
| *EDUCATION* | 0.139*** | 0.039 | 0.009** | 0.005 | 0.148*** | 0.039 |
| *MARRIAGE* | 0.452*** | 0.129 | - | (no path) | 0.452*** | 0.129 |
| *IV_HAPPINESS* | 0.867*** | 0.132 | - | (no path) | 0.867*** | 0.132 |

Note: *** p<0.01, ** p<0.05, * p<0.1

Table E-3　Direct, indirect, and total associations : India

|  | *HEIGHT* | | | | | |
| --- | --- | --- | --- | --- | --- | --- |
|  | Direct | | Indirect | | Total | |
|  | Coef. | Std. Err. | Coef. | Std. Err. | Coef. | Std. Err. |
| *LBW* | -0.012 | 0.015 | -0.002 | 0.001 | -0.013 | 0.015 |
| *LBW×OLD* | -0.019 | 0.025 | 0.002 | 0.002 | -0.017 | 0.025 |
| *Q_HBW* | 0.021 | 0.032 | 0.005 | 0.004 | 0.025 | 0.032 |
| *Q_HBW×OLD* | -0.050 | 0.050 | -0.001 | 0.003 | -0.051 | 0.050 |
| *DONTKNOW* | -0.014 | 0.011 | -0.002 | 0.001 | -0.016 | 0.011 |
| *DONTKNOW×OLD* | 0.016 | 0.016 | 0.001 | 0.001 | 0.017 | 0.016 |
| *OLD* | -0.007 | 0.017 | -0.002 | 0.002 | -0.009 | 0.017 |
| *MALE* | 0.052*** | 0.010 | 0.001 | 0.001 | 0.053*** | 0.010 |
| *ACADEMIC* | 0.006 | 0.004 | - | (no path) | 0.006 | 0.004 |
|  | *EDUCATION* | | | | | |
|  | Direct | | Indirect | | Total | |
|  | Coef. | Std. Err. | Coef. | Std. Err. | Coef. | Std. Err. |
| *LBW* | -0.338 | 0.224 | -0.094 | 0.059 | -0.431* | 0.230 |
| *LBW×OLD* | 0.293 | 0.389 | 0.117 | 0.101 | 0.409 | 0.400 |
| *Q_HBW* | -0.171 | 0.488 | 0.270** | 0.132 | 0.099 | 0.500 |
| *Q_HBW×OLD* | 0.442 | 0.770 | -0.109 | 0.196 | 0.334 | 0.793 |
| *DONTKNOW* | -0.066 | 0.173 | -0.108** | 0.048 | -0.174 | 0.177 |
| *DONTKNOW×OLD* | 0.557** | 0.254 | 0.083 | 0.066 | 0.640** | 0.261 |
| *OLD* | -0.550** | 0.263 | -0.137 | 0.070 | -0.687** | 0.270 |
| *ACADEMIC* | 0.320*** | 0.061 | 0.003 | 0.004 | 0.322*** | 0.061 |
| *HEIGHT* | 0.456 | 0.726 | - | (no path) | 0.456 | 0.726 |
|  | *MARRIAGE* | | | | | |
|  | Direct | | Indirect | | Total | |
|  | Coef. | Std. Err. | Coef. | Std. Err. | Coef. | Std. Err. |
| *LBW* | 0.079 | 0.067 | 0.003 | 0.008 | 0.082 | 0.066 |
| *LBW×OLD* | -0.009 | 0.116 | -0.004 | 0.010 | -0.013 | 0.116 |
| *Q_HBW* | 0.016 | 0.145 | -0.005 | 0.016 | 0.011 | 0.145 |
| *Q_HBW×OLD* | 0.099 | 0.230 | -0.001 | 0.014 | 0.098 | 0.229 |
| *DONTKNOW* | -0.027 | 0.052 | 0.002 | 0.007 | -0.025 | 0.051 |
| *DONTKNOW×OLD* | 0.057 | 0.076 | -0.003 | 0.010 | 0.054 | 0.075 |
| *OLD* | -0.219*** | 0.079 | 0.005 | 0.011 | -0.214*** | 0.078 |
| *ACADEMIC* | -0.006 | 0.019 | -0.001 | 0.005 | -0.007 | 0.018 |
| *HEIGHT* | 0.029 | 0.216 | -0.002 | 0.007 | 0.027 | 0.216 |
| *EDUCATION* | -0.004 | 0.014 | - | (no path) | -0.004 | 0.014 |
|  | *BMI* | | | | | |
|  | Direct | | Indirect | | Total | |
|  | Coef. | Std. Err. | Coef. | Std. Err. | Coef. | Std. Err. |
| *LBW* | -0.441 | 0.600 | 0.344 | 0.311 | -0.097 | 0.668 |
| *LBW×OLD* | 2.695*** | 1.042 | 0.292 | 0.533 | 2.987*** | 1.164 |
| *Q_HBW* | 3.053** | 1.303 | -0.697 | 0.673 | 2.356 | 1.453 |
| *Q_HBW×OLD* | -1.114 | 2.057 | 1.251 | 1.050 | 0.136 | 2.302 |
| *DONTKNOW* | -0.256 | 0.463 | 0.339 | 0.240 | 0.083 | 0.514 |
| *DONTKNOW×OLD* | 0.483 | 0.689 | -0.227 | 0.354 | 0.256 | 0.763 |
| *OLD* | -0.024 | 0.711 | -0.051 | 0.379 | -0.075 | 0.783 |
| *ACADEMIC* | -0.274 | 0.169 | -0.058 | 0.092 | -0.332* | 0.184 |
| *HEIGHT* | -19.891*** | 1.961 | 0.112 | 0.260 | -19.779*** | 1.976 |
| *EDUCATION* | 0.187 | 0.126 | -0.004 | 0.014 | 0.183 | 0.127 |
| *MARRIAGE* | 0.997** | 0.422 | - | (no path) | 0.997** | 0.422 |
| *IV_BMI* | 0.769*** | 0.099 | - | (no path) | 0.769*** | 0.099 |
|  | *INCOME* | | | | | |
|  | Direct | | Indirect | | Total | |
|  | Coef. | Std. Err. | Coef. | Std. Err. | Coef. | Std. Err. |
| *LBW* | -3.017** | 1.351 | -0.441 | 0.528 | -3.458** | 1.431 |
| *LBW×OLD* | -1.324 | 2.353 | 0.858 | 0.892 | -0.466 | 2.502 |
| *Q_HBW* | 1.117 | 2.936 | -0.127 | 1.135 | 0.991 | 3.115 |
| *Q_HBW×OLD* | 4.790 | 4.633 | 1.457 | 1.751 | 6.246 | 4.936 |
| *DONTKNOW* | -1.527 | 1.044 | -0.230 | 0.409 | -1.757 | 1.105 |
| *DONTKNOW×OLD* | 2.256 | 1.533 | 1.240** | 0.603 | 3.496** | 1.623 |
| *OLD* | 0.913 | 1.601 | -1.859*** | 0.662 | -0.946 | 1.678 |
| *ACADEMIC* | -0.161 | 0.380 | 0.566*** | 0.166 | 0.404 | 0.393 |
| *HEIGHT* | -8.936** | 4.368 | 0.985 | 1.576 | -7.952* | 4.640 |
| *EDUCATION* | 1.980*** | 0.285 | -0.011 | 0.043 | 1.968*** | 0.288 |
| *MARRIAGE* | 3.016*** | 0.952 | - | (no path) | 3.016*** | 0.952 |
| *IV_INCOME* | 0.796*** | 0.112 | - | (no path) | 0.796*** | 0.112 |
|  | *HEALTH* | | | | | |
|  | Direct | | Indirect | | Total | |
|  | Coef. | Std. Err. | Coef. | Std. Err. | Coef. | Std. Err. |
| *LBW* | -0.305** | 0.130 | -0.006 | 0.024 | -0.311** | 0.130 |
| *LBW×OLD* | -0.257 | 0.225 | 0.033 | 0.037 | -0.224 | 0.227 |
| *Q_HBW* | 0.881*** | 0.281 | -0.015 | 0.050 | 0.867*** | 0.283 |
| *Q_HBW×OLD* | -0.375 | 0.444 | 0.069 | 0.070 | -0.306 | 0.448 |
| *DONTKNOW* | -0.113 | 0.100 | -0.001 | 0.019 | -0.114 | 0.100 |
| *DONTKNOW×OLD* | -0.185 | 0.155 | 0.033 | 0.028 | -0.151 | 0.155 |
| *OLD* | 0.144 | 0.154 | -0.060* | 0.036 | 0.084 | 0.153 |
| *ACADEMIC* | -0.006 | 0.036 | 0.015 | 0.010 | 0.009 | 0.036 |
| *HEIGHT* | -0.676 | 0.419 | 0.032 | 0.053 | -0.644 | 0.422 |
| *EDUCATION* | 0.062** | 0.027 | -0.0005 | 0.002 | 0.062** | 0.027 |
| *MARRIAGE* | 0.120 | 0.091 | - | (no path) | 0.120 | 0.091 |
| *IV_HEALTH* | 0.958*** | 0.108 | - | (no path) | 0.958*** | 0.108 |
|  | *HAPPINESS* | | | | | |
|  | Direct | | Indirect | | Total | |
|  | Coef. | Std. Err. | Coef. | Std. Err. | Coef. | Std. Err. |
| *LBW* | -0.028 | 0.198 | -0.050 | 0.037 | -0.078 | 0.199 |
| *LBW×OLD* | -0.179 | 0.344 | 0.041 | 0.056 | -0.138 | 0.347 |
| *Q_HBW* | 0.522 | 0.431 | 0.107 | 0.077 | 0.628 | 0.433 |
| *Q_HBW×OLD* | -0.241 | 0.682 | -0.041 | 0.106 | -0.282 | 0.687 |
| *DONTKNOW* | -0.308** | 0.153 | -0.057* | 0.029 | -0.365** | 0.153 |
| *DONTKNOW×OLD* | 0.092 | 0.228 | 0.078* | 0.043 | 0.170 | 0.228 |
| *OLD* | -0.010 | 0.236 | -0.112** | 0.055 | -0.122 | 0.234 |
| *ACADEMIC* | 0.095* | 0.056 | 0.021 | 0.015 | 0.116** | 0.055 |
| *HEIGHT* | 0.910 | 0.642 | 0.028 | 0.051 | 0.938 | 0.644 |
| *EDUCATION* | 0.052 | 0.042 | -0.001 | 0.002 | 0.052 | 0.042 |
| *MARRIAGE* | 0.134 | 0.140 | - | (no path) | 0.134 | 0.140 |
| *IV_HAPPINESS* | 0.974*** | 0.090 | - | (no path) | 0.974*** | 0.090 |

Note: *** p<0.01, ** p<0.05, * p<0.1

Table E-4 Comparison of the estimates by FIML with those by recursive-structural form and by reduced form: Japan

|  | FIML | | | | | | Recursive-structural | | Reduced | | Comparison | |
| --- | --- | --- | --- | --- | --- | --- | --- | --- | --- | --- | --- | --- |
|  | Direct association | | Indirect association | | Total association | | R-S | | Reduced | | Direct | Total |
|  | Coef. | p-value | Coef. | p-value | Coef. | p-value | Coef. | p-value | Coef. | p-value | FIML/R-S | FIML/reduced |
| ***ACADEMIC*** | |  |  |  |  |  |  |  |  |  |  |  |
| *LBW* | -0.1211 | 0.3080 |  |  | -0.1211 | 0.3080 | -0.1974 | 0.0530 | -0.1974 | 0.0530 | 0.6134 | 0.6134 |
| *HBW* | -0.2800 | 0.1370 |  |  | -0.2800 | 0.1370 | -0.2264 | 0.1670 | -0.2264 | 0.1670 | 1.2369 | 1.2369 |
| ***HEIGHT*** |  |  |  |  |  |  |  |  |  |  |  |  |
| *LBW* | -0.0287 | 0.0000 | -0.0003 | 0.3370 | -0.0290 | 0.0000 | -0.0282 | 0.0000 | -0.0280 | 0.0000 | **1.0163** | **1.0358** |
| *HBW* | 0.0386 | 0.0000 | -0.0008 | 0.1870 | 0.0378 | 0.0000 | 0.0390 | 0.0000 | 0.0392 | 0.0000 | **0.9886** | **0.9635** |
| ***EDUCATION*** |  |  |  |  |  |  |  |  |  |  |  |  |
| *LBW* | -0.3694 | 0.0460 | -0.1012 | 0.2200 | -0.4706 | 0.0190 | -0.3237 | 0.0300 | -0.4564 | 0.0020 | **1.1413** | **1.0311** |
| *HBW* | -0.3164 | 0.2810 | -0.1644 | 0.2070 | -0.4807 | 0.1320 | -0.1419 | 0.6450 | -0.2114 | 0.5150 | 2.2294 | 2.2744 |
| ***MARRIAGE*** | |  |  |  |  |  |  |  |  |  |  |  |
| *LBW* | 0.0843 | 0.0520 | -0.0040 | 0.3970 | 0.0803 | 0.0630 | 0.0685 | 0.0400 | 0.0569 | 0.0880 | **1.2322** | **1.4108** |
| *HBW* | 0.0703 | 0.3060 | 0.0023 | 0.7220 | 0.0726 | 0.2890 | 0.0217 | 0.7350 | 0.0093 | 0.8870 | 3.2328 | 7.8342 |
| ***BMI*** |  |  |  |  |  |  |  |  |  |  |  |  |
| *LBW* | 0.7918 | 0.0210 | 0.0388 | 0.3290 | 0.8306 | 0.0150 | 0.6886 | 0.0770 | 0.7840 | 0.0140 | **1.1499** | **1.0594** |
| *HBW* | 1.2831 | 0.0180 | -0.0697 | 0.1850 | 1.2134 | 0.0250 | 1.1459 | 0.0250 | 1.1627 | 0.0140 | **1.1197** | **1.0435** |
| ***INCOME*** |  |  |  |  |  |  |  |  |  |  |  |  |
| *LBW* | -0.2801 | 0.3580 | -0.2033 | 0.0180 | -0.4834 | 0.1230 | -0.4013 | 0.0990 | -0.5316 | 0.0150 | 0.6980 | 0.9093 |
| *HBW* | 0.2340 | 0.6280 | -0.0891 | 0.5030 | 0.1449 | 0.7710 | -0.1780 | 0.7050 | -0.0830 | 0.8520 | -1.3143 | -1.7460 |
| ***HEALTH*** |  |  |  |  |  |  |  |  |  |  |  |  |
| *LBW* | -0.1149 | 0.2740 | 0.0150 | 0.4420 | -0.0998 | 0.3450 | -0.0672 | 0.5370 | -0.2512 | 0.0070 | 1.7103 | 0.3974 |
| *HBW* | 0.0353 | 0.8320 | -0.0057 | 0.8440 | 0.0296 | 0.8600 | 0.0354 | 0.8010 | 0.0263 | 0.8500 | 0.9984 | 1.1233 |
| ***HAPPINESS*** | |  |  |  |  |  |  |  |  |  |  |  |
| *LBW* | -0.3259 | 0.0900 | 0.0055 | 0.9220 | -0.3205 | 0.1050 | -0.1960 | 0.3300 | -0.4242 | 0.0110 | **1.6631** | **0.7554** |
| *HBW* | 0.2073 | 0.4960 | 0.0233 | 0.7880 | 0.2306 | 0.4630 | 0.0840 | 0.7650 | -0.0480 | 0.8690 | 2.4670 | -4.8010 |

Note: R-S stands for the estimates of recursive-structural form. FIML/R-S represents the ratio of (estimates of direct association by FIML / the estimates of recursive-structural-form). FIML/reduced represents the ratio of estimates of (total association by FIML / the estimates of reduced form). Figures in red represent the ratios when the estimates of direct association by FIML were significant at the 10%.

Table E-5 Comparison of the estimates by FIML with those by recursive-structural form and by reduced form: the U.S.

|  | FIML | | | | | | Recursive-structural | | Reduced | | Comparison | |
| --- | --- | --- | --- | --- | --- | --- | --- | --- | --- | --- | --- | --- |
|  | Direct association | | Indirect association | | Total association | | 2SLS | | Reduced | | Direct | Total |
|  | Coef. | p-value | Coef. | p-value | Coef. | p-value | Coef. | p-value | Coef. | p-value | FIML/2SLS | FIML/reduced |
| ***ACADEMIC*** |  |  |  |  |  |  |  |  |  |  |  |  |
| *LBW* | -0.0502 | 0.7960 |  |  | -0.0502 | 0.7960 | -0.0273 | 0.8630 | -0.0273 | 0.8630 | 1.8393 | 1.8393 |
| *HBW* | 0.0085 | 0.9360 |  |  | 0.0085 | 0.9360 | 0.0398 | 0.6620 | 0.0398 | 0.6620 | 0.2143 | 0.2143 |
| *V_HBW* | -0.1154 | 0.5810 |  |  | -0.1154 | 0.5810 | -0.0079 | 0.9670 | -0.0079 | 0.9670 | 14.5686 | 14.5686 |
| ***HEIGHT*** |  |  |  |  |  |  |  |  |  |  |  |  |
| *LBW* | -0.0047 | 0.7260 | 0.0002 | 0.7970 | -0.0045 | 0.7390 | -0.0114 | 0.3300 | -0.0096 | 0.4050 | 0.4119 | 0.4687 |
| *HBW* | 0.0166 | 0.0240 | 0.0000 | 0.9360 | 0.0165 | 0.0250 | 0.0186 | 0.0060 | 0.0187 | 0.0050 | 0.8918 | 0.8861 |
| *V_HBW* | 0.0576 | 0.0000 | 0.0005 | 0.5900 | 0.0581 | 0.0000 | 0.0674 | 0.0000 | 0.0658 | 0.0000 | 0.8543 | 0.8829 |
| ***EDUCATION*** |  |  |  |  |  |  |  |  |  |  |  |  |
| *LBW* | -0.2216 | 0.4320 | -0.0320 | 0.7880 | -0.2536 | 0.4080 | -0.0785 | 0.6910 | -0.1268 | 0.5430 | 2.8211 | 2.0001 |
| *HBW* | -0.1076 | 0.4860 | 0.0093 | 0.8870 | -0.0982 | 0.5580 | -0.0367 | 0.7930 | 0.0408 | 0.7770 | 2.9273 | -2.4066 |
| *V_HBW* | -0.2598 | 0.3950 | -0.0567 | 0.6690 | -0.3165 | 0.3370 | -0.3152 | 0.3220 | -0.2201 | 0.4770 | 0.8243 | 1.4379 |
| ***MARRIAGE*** |  |  |  |  |  |  |  |  |  |  |  |  |
| *LBW* | 0.0119 | 0.8900 | -0.0056 | 0.4060 | 0.0063 | 0.9420 | 0.0018 | 0.9820 | -0.0380 | 0.5970 | 6.6370 | -0.1663 |
| *HBW* | -0.0962 | 0.0410 | 0.0012 | 0.8030 | -0.0950 | 0.0440 | -0.0941 | 0.0250 | -0.1046 | 0.0080 | 1.0223 | 0.9085 |
| *V_HBW* | 0.0039 | 0.9660 | 0.0061 | 0.6240 | 0.0100 | 0.9140 | 0.0767 | 0.3360 | 0.0280 | 0.7260 | 0.0512 | 0.3582 |
| ***BMI*** |  |  |  |  |  |  |  |  |  |  |  |  |
| *LBW* | -1.2682 | 0.3070 | 0.1074 | 0.5600 | -1.1608 | 0.3540 | -0.8381 | 0.5050 | -1.4180 | 0.2430 | 1.5131 | 0.8186 |
| *HBW* | 1.2102 | 0.0750 | 0.0238 | 0.8350 | 1.2340 | 0.0720 | 1.2518 | 0.0990 | 1.2598 | 0.0750 | 0.9667 | 0.9795 |
| *V_HBW* | 3.7412 | 0.0050 | -0.3577 | 0.1460 | 3.3835 | 0.0120 | 3.1480 | 0.0700 | 3.0721 | 0.0420 | 1.1884 | 1.1014 |
| ***INCOME*** |  |  |  |  |  |  |  |  |  |  |  |  |
| *LBW* | 0.2353 | 0.7140 | -0.1902 | 0.4340 | 0.0450 | 0.9480 | 0.1966 | 0.7640 | -0.2987 | 0.5320 | 1.1964 | -0.1507 |
| *HBW* | 0.0681 | 0.8470 | -0.1061 | 0.4350 | -0.0380 | 0.9190 | -0.0962 | 0.8310 | -0.1650 | 0.6440 | -0.7081 | 0.2305 |
| *V_HBW* | -0.0853 | 0.9020 | -0.1765 | 0.5170 | -0.2619 | 0.7230 | -0.9506 | 0.1260 | -0.5404 | 0.3130 | 0.0898 | 0.4846 |
| ***HEALTH*** |  |  |  |  |  |  |  |  |  |  |  |  |
| *LBW* | -0.0345 | 0.8370 | -0.0231 | 0.5200 | -0.0577 | 0.7370 | -0.0416 | 0.8020 | 0.1263 | 0.3170 | 0.8307 | -0.4567 |
| *HBW* | 0.0217 | 0.8130 | -0.0235 | 0.2630 | -0.0018 | 0.9840 | 0.0218 | 0.8050 | -0.0638 | 0.4300 | 0.9977 | 0.0286 |
| *V_HBW* | -0.4152 | 0.0220 | -0.0078 | 0.8580 | -0.4230 | 0.0220 | -0.3009 | 0.1190 | -0.4192 | 0.0080 | 1.3799 | 1.0090 |
| ***HAPPINESS*** |  |  |  |  |  |  |  |  |  |  |  |  |
| *LBW* | 0.3644 | 0.3730 | -0.0402 | 0.5360 | 0.3242 | 0.4340 | 0.3149 | 0.4530 | 0.4062 | 0.1980 | 1.1570 | 0.7981 |
| *HBW* | -0.1381 | 0.5390 | -0.0309 | 0.4390 | -0.1690 | 0.4560 | -0.0371 | 0.8680 | -0.1512 | 0.4520 | 3.7205 | 1.1172 |
| *V_HBW* | -0.6492 | 0.1430 | 0.0484 | 0.5680 | -0.6009 | 0.1780 | -0.1192 | 0.8170 | -0.8936 | 0.0500 | 5.4459 | 0.6724 |

Note: R-S stands for the estimates of recursive-structural form. FIML/R-S represents the ratio of (estimates of direct association by FIML / the estimates of recursive-structural-form). FIML/reduced represents the ratio of estimates of (total association by FIML / the estimates of reduced form). Figures in red represent the ratios when the estimates of direct association by FIML were significant at the 10%.

Table E-6 Comparison of the estimates by FIML with those by recursive-structural form and by reduced form: India

|  | FIML | | | | | | Recursive-structural | | Reduced | | Comparison | |
| --- | --- | --- | --- | --- | --- | --- | --- | --- | --- | --- | --- | --- |
|  | Direct association | | Indirect association | | Total association | | 2SLS | | Reduced | | Direct | Total |
|  | Coef. | p-value | Coef. | p-value | Coef. | p-value | Coef. | p-value | Coef. | p-value | FIML/2SLS | FIML/reduced |
| ***ACADEMIC*** |  |  |  |  |  |  |  |  |  |  |  |  |
| *LBW* | -0.2736 | 0.1110 |  |  | -0.2736 | 0.1110 | -0.1423 | 0.2670 | -0.1423 | 0.2670 | 1.9224 | 1.9224 |
| *Q_HBW* | 0.8099 | 0.0300 |  |  | 0.8099 | 0.0300 | 0.4279 | 0.1140 | 0.4279 | 0.1140 | **1.8929** | **1.8929** |
| ***HEIGHT*** |  |  |  |  |  |  |  |  |  |  |  |  |
| *LBW* | -0.0120 | 0.4110 | -0.0015 | 0.2930 | -0.0135 | 0.3530 | -0.0135 | 0.1720 | -0.0172 | 0.0890 | 0.8860 | 0.7825 |
| *Q_HBW* | 0.0209 | 0.5100 | 0.0045 | 0.2400 | 0.0254 | 0.4220 | 0.0133 | 0.5490 | 0.0143 | 0.5150 | 1.5674 | 1.7812 |
| ***EDUCATION*** |  |  |  |  |  |  |  |  |  |  |  |  |
| *LBW* | -0.3376 | 0.1310 | -0.0936 | 0.1110 | -0.4312 | 0.0610 | -0.3767 | 0.0030 | -0.5127 | 0.0010 | 0.8963 | 0.8410 |
| *Q_HBW* | -0.1712 | 0.7260 | 0.2704 | 0.0400 | 0.0992 | 0.8430 | -0.2274 | 0.4830 | -0.2038 | 0.5480 | 0.7531 | -0.4869 |
| ***MARRIAGE*** |  |  |  |  |  |  |  |  |  |  |  |  |
| *LBW* | 0.0793 | 0.2350 | 0.0030 | 0.6930 | 0.0823 | 0.2160 | 0.0869 | 0.0440 | 0.0889 | 0.0430 | 0.9131 | 0.9254 |
| *Q_HBW* | 0.0162 | 0.9110 | -0.0048 | 0.7650 | 0.0114 | 0.9370 | 0.0574 | 0.4670 | 0.0335 | 0.6750 | 0.2814 | 0.3394 |
| ***BMI*** |  |  |  |  |  |  |  |  |  |  |  |  |
| *LBW* | -0.4411 | 0.6150 | 0.3443 | 0.2690 | -0.0969 | 0.8850 | -0.6264 | 0.3380 | -0.6831 | 0.1880 | 0.7042 | 0.1418 |
| *Q_HBW* | 3.0528 | 0.0230 | -0.6966 | 0.3010 | 2.3562 | 0.1050 | 3.1288 | 0.1100 | 2.9668 | 0.0150 | **0.9757** | **0.7942** |
| ***INCOME*** |  |  |  |  |  |  |  |  |  |  |  |  |
| *LBW* | -3.0169 | 0.0260 | -0.4408 | 0.4040 | -3.4577 | 0.0160 | -2.3917 | 0.0480 | -34.2848 | 0.0110 | **1.2614** | **0.1009** |
| *Q_HBW* | 1.1175 | 0.7030 | -0.1268 | 0.9110 | 0.9906 | 0.7510 | -0.8511 | 0.7900 | 3.0622 | 0.9180 | -1.3129 | 0.3235 |
| ***HEALTH*** |  |  |  |  |  |  |  |  |  |  |  |  |
| *LBW* | -0.3047 | 0.0190 | -0.0062 | 0.8010 | -0.3109 | 0.0170 | -0.1615 | 0.3470 | -0.1995 | 0.0440 | **1.8866** | **1.5580** |
| *Q_HBW* | 0.8815 | 0.0020 | -0.0147 | 0.7710 | 0.8668 | 0.0020 | 0.7690 | 0.0000 | 0.6885 | 0.0000 | **1.1462** | **1.2590** |
| ***HAPPINESS*** |  |  |  |  |  |  |  |  |  |  |  |  |
| *LBW* | -0.0280 | 0.8880 | -0.0498 | 0.1830 | -0.0778 | 0.6960 | 0.0237 | 0.9260 | -0.1581 | 0.2870 | -1.1818 | 0.4923 |
| *Q_HBW* | 0.5218 | 0.2260 | 0.1066 | 0.1660 | 0.6284 | 0.1470 | -0.3079 | 0.4190 | 0.4231 | 0.0470 | -1.6948 | 1.4852 |

Note: R-S stands for the estimates of recursive-structural form. FIML/R-S represents the ratio of (estimates of direct association by FIML / the estimates of recursive-structural form). FIML/reduced represents the ratio of estimates of (total association by FIML / the estimates of reduced form). Figures in red represent the ratios when the estimates of direct association by FIML were significant at the 10%.

1. The estimates of the direct effect are same as those in Tables D-1 to D-3. [↑](#footnote-ref-1)
