# Supplementary material for: Does birthweight matter to quality of life? A comparison between Japan, the U.S., and India
Source: Health Econ Rev. 2022 Sep 20;12:48. doi: 10.1186/s13561-022-00393-9 (PMC9487066; doi:10.1186/s13561-022-00393-9)
Supplement: Supplementary file 6 — Additional file 6: Supplemental material F. The estimation results using birthweight instead of birthweight dummies: U.S. [file 13561_2022_393_MOESM6_ESM.docx]

# Supplemental material F: The estimation results using birthweight instead of birthweight dummies: U.S.

In this section, we present the estimation results of eq. (1) for the U.S. substituting “birthweight” for *LBW*, *HBW*, and *V_HBW*. For the U.S., the response category in the original questionnaire asking birthweight ranged from 1 (less than 5.5 lb) to 5 (10 lb or more) and 6 (= do not know). Therefore, we defined “*BWEIGHT*” using 1 through 5 representing these categories, excluding the category 6.

Table F-1 presents the estimation results. It reveals that *BWEIGHT* is significantly and positively associated with *HEIGHT* and *BMI*, but is significantly and negatively associated with *HEALTH* and *HAPPINESS*. These results are consistent with the estimates shown in Table 4-2 in the text: i.e., *V_HBW* were significantly and positively associated with *HEIGHT* and *BMI*, but were significantly and negatively associated with *HEALTH* and *HAPPINESS*. Therefore, insignificant estimates on *LBW* in the U.S. does not contradict the results of previous studies, which showed that birthweight is significantly associated with some outcomes of life.

Table F-1 The estimates for birthweight: the U.S.

|  | *ACADEMIC* | *HEIGHT* | *EDUCATION* | *MARRIAGE* | *BMI* | *INCOME* | *HEALTH* | *HAPPINESS* |
| --- | --- | --- | --- | --- | --- | --- | --- | --- |
| *BWEIGHT* | 0.0412 | 0.0150*** | -0.0191 | 0.00372 | 0.927*** | -0.117 | -0.0814** | -0.164* |
|  | (0.0398) | (0.00317) | (0.0598) | (0.0172) | (0.308) | (0.128) | (0.0333) | (0.0866) |
| *BWEIGHT×OLD* | -0.00708 | -0.00757* | 0.0660 | -0.0243 | -0.561 | 0.210 | 0.108** | 0.172 |
|  | (0.0528) | (0.00455) | (0.0862) | (0.0239) | (0.407) | (0.199) | (0.0482) | (0.118) |
| *OLD* | 0.000389 | 0.0230* | -0.131 | -0.0212 | 1.438 | -0.741 | -0.282* | -0.451 |
|  | (0.166) | (0.0132) | (0.271) | (0.0759) | (1.191) | (0.615) | (0.152) | (0.378) |
| *MALE* | -0.224*** | 0.139*** | 0.0160 | 0.0182 | 0.408 | 1.928*** | 0.0548 | 0.207* |
|  | (0.0509) | (0.00378) | (0.0844) | (0.0225) | (0.345) | (0.206) | (0.0463) | (0.111) |
| *AGE* | -1.103 | 0.0464 | 5.898*** | 5.962*** | 34.50*** | 39.98*** | -2.697*** | -5.140*** |
|  | (0.944) | (0.0704) | (1.483) | (0.400) | (6.977) | (3.200) | (0.837) | (1.986) |
| *AGESQ* | 1.471* | -0.0727 | -4.482*** | -5.127*** | -34.25*** | -35.56*** | 1.468** | 5.739*** |
|  | (0.830) | (0.0610) | (1.386) | (0.375) | (5.805) | (2.997) | (0.741) | (1.738) |
| *F_EDUCATION* | 0.0351** | 0.000886 | 0.218*** | 0.00216 | -0.349*** | 0.271*** | 0.0520*** | 0.0365 |
|  | (0.0160) | (0.00122) | (0.0261) | (0.00688) | (0.109) | (0.0706) | (0.0139) | (0.0361) |
| *M_EDUCATION* | 0.0920*** | 0.00430*** | 0.186*** | -0.0207*** | -0.198* | 0.0432 | 0.0212 | 0.00478 |
|  | (0.0180) | (0.00136) | (0.0310) | (0.00793) | (0.120) | (0.0734) | (0.0164) | (0.0400) |
| *F_AGE_BIRTH* | 0.0675 | 0.00320 | 0.262 | -0.383 | -2.008 | 0.711 | -0.466 | 0.811 |
|  | (0.603) | (0.0426) | (0.996) | (0.272) | (4.004) | (2.306) | (0.521) | (1.277) |
| *M_AGE_BIRTH* | 0.358 | -0.00339 | 2.630** | -0.0508 | -2.212 | 1.927 | 1.300** | 0.541 |
|  | (0.686) | (0.0522) | (1.146) | (0.304) | (4.680) | (2.526) | (0.617) | (1.524) |
| *M_FULLTIME* | 0.169 | -0.0192 | 0.283 | 0.113 | 0.496 | 1.236*** | 0.219 | -0.247 |
|  | (0.192) | (0.0118) | (0.260) | (0.0726) | (1.252) | (0.460) | (0.145) | (0.397) |
| *M_PARTTIME* | 0.291 | -0.0103 | 0.507* | 0.113 | -0.325 | 1.385*** | 0.294** | -0.0668 |
|  | (0.194) | (0.0121) | (0.267) | (0.0739) | (1.273) | (0.487) | (0.148) | (0.405) |
| *M_HOUSEWIFE* | 0.249 | -0.0185 | 0.435 | 0.129* | 0.155 | 1.127** | 0.229 | -0.0230 |
|  | (0.193) | (0.0123) | (0.264) | (0.0737) | (1.283) | (0.483) | (0.148) | (0.403) |
| *S_LIVING* | 0.0244* | 0.00153 | 0.0442** | 0.00291 | -0.172* | 0.0389 | 0.0223* | 0.0608* |
|  | (0.0135) | (0.00100) | (0.0213) | (0.00610) | (0.0983) | (0.0497) | (0.0130) | (0.0321) |
| *ONLYCHILD* | 0.0772 | -0.000927 | 0.325** | -0.0697 | 0.741 | -0.0646 | -0.115 | -0.379* |
|  | (0.0884) | (0.00672) | (0.159) | (0.0452) | (0.701) | (0.446) | (0.0910) | (0.226) |
| *RELIGION* | 0.0381** | 0.00127 | 0.0487 | 0.0354*** | 0.0513 | -0.0677 | 0.0381** | 0.207*** |
|  | (0.0191) | (0.00139) | (0.0308) | (0.00825) | (0.136) | (0.0753) | (0.0170) | (0.0418) |
| Cons | 2.808*** | 1.587*** | 0.341 | -0.976*** | 21.84*** | -9.641*** | 3.780*** | 7.487*** |
|  | (0.389) | (0.0276) | (0.591) | (0.163) | (2.854) | (1.264) | (0.339) | (0.815) |
| Obs. | 1,722 | 1,584 | 1,761 | 1,749 | 1,548 | 1,381 | 1,767 | 1,708 |
| R^2^ | 0.086 | 0.521 | 0.178 | 0.193 | 0.089 | 0.195 | 0.128 | 0.060 |

Note: The region dummies representing the state where respondents lived at age 15 are included in the estimation, but not shown here to save space. Robust standard errors in parentheses *** p<0.01, ** p<0.05, * p<0
